# A novel pillar-layered MOF with urea linkers as a capable catalyst for synthesis of new 1,8-naphthyridines via the anomeric-based oxidation

**DOI:** 10.1038/s41598-024-66539-3

**Published:** 2024-11-12

**Authors:** Masoumeh Beiranvand, Davood Habibi, Hosein Khodakarami

**Affiliations:** Department of Organic Chemistry, Faculty of Chemistry and Petroleum Sciences, Bu-Ali Sina University, Hamedan, Iran

**Keywords:** Metal–organic framework, Heterogeneous, 1,8-Naphthyridines, Tandem, Anomeric, Chemistry, Materials science

## Abstract

Metal-based catalysts play an essential role in organic chemistry and the chemical industry. This research designed and successfully synthesized a pillar-layered metal–organic framework (MOF) with the urea linkers, namely Basu-HDI, as a novel and efficient heterogeneous catalyst. Various techniques such as FT-IR, EDX, elemental mapping, SEM, XRD, BET, and TGA/DTA studied its structure and morphology. Then, we investigated the synthesis of new 1,8-naphthyridines utilizing Basu-HDI in mild conditions via a one‐pot, three‐component tandem Knoevenagel/Michael/ cyclization/anomeric-based oxidation reaction. Final products were achieved by anomeric-based oxidation without employing an oxidation agent. Remarkably, this tandem process gave a good range of new 1,8-naphthyridines with high yields in a short reaction time. The pure products were confirmed by FT-IR, ^1^H NMR, ^13^C NMR, and mass spectrometry techniques. Moreover, the introduced catalyst showed good efficiency and stability and can be reused four times without significantly reducing efficiency.

## Introduction

Metal-based catalysts can produce attractive scaffolds through a single catalytic pathway by activating the starting materials and selectively controlling the reaction^1^. Inspired by these characteristics, the utilization of metal–organic frameworks (MOFs) as heterogeneous catalysts in multicomponent reactions (MCRs) has significantly increased in recent years because it can achieve different metal coordination environments in active solids^2^.

**Figure 1:**
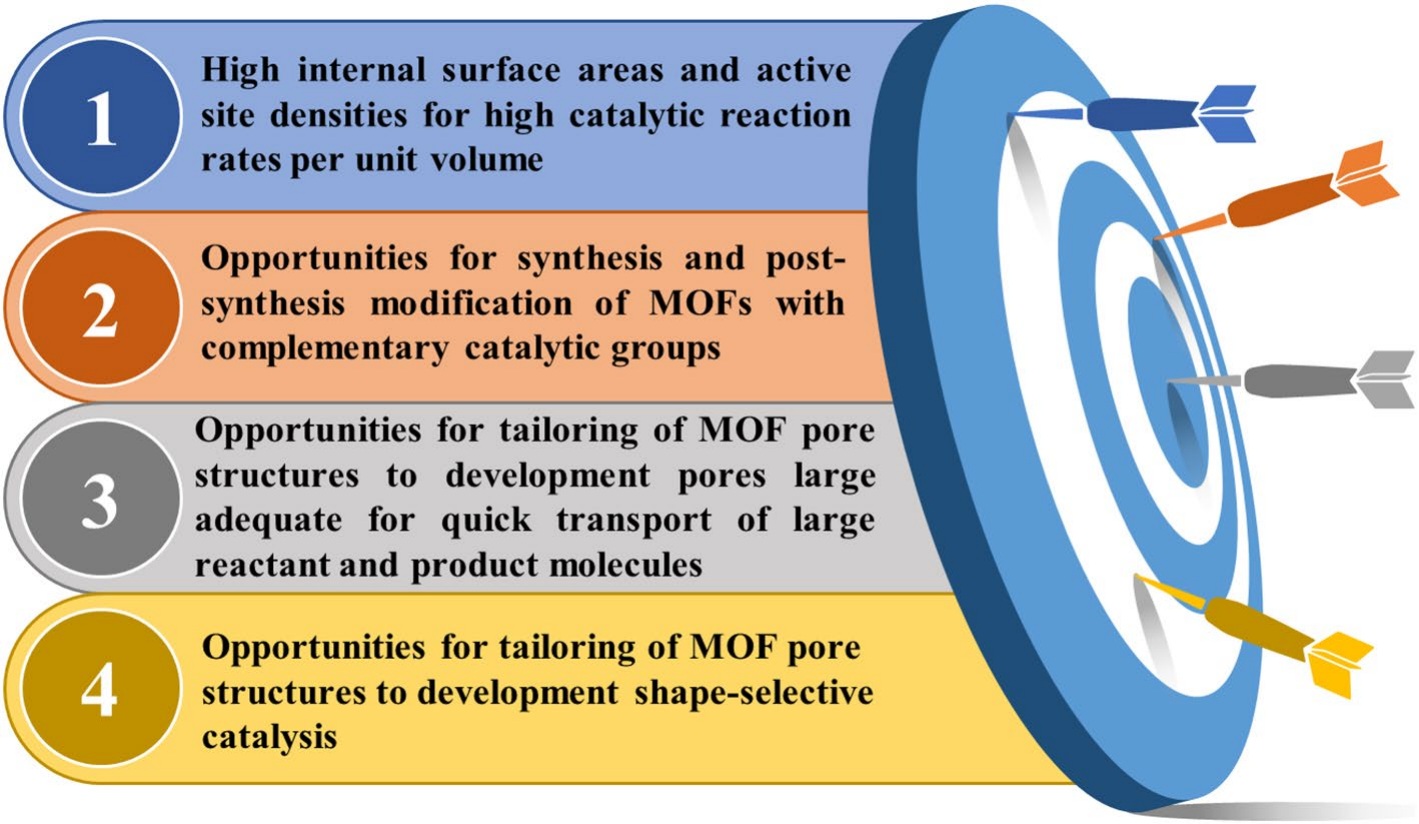
Extraordinary properties of MOFs.

The MOFs are two- or three-dimensional porous crystalline materials of hybrid organic/ inorganic nature fabricated by metal ions and multidentate organic ligands through reticular synthesis^3,4^. MOFs have received much attention in the last two decades because they have indicated terrific performances in gas storage, catalysis, drug delivery, separation, etc., due to their high surface areas and tunable pore sizes^5,6^. The Zr-MOFs possess higher chemical and thermal stability among reported MOFs due to the strong Zr-carboxylate coordination bond^7^. The catalysis success of MOFs can be attributed to their extraordinary properties (Fig. 1)^8^.

MCRs are one of the best strategies for synthesizing complex scaffolds in a single process in which three or more components react to generate a single product^9,10^. The main advantages of this strategy are high atom economy, high efficiency, reduction of waste and by-products, time-saving and easy work-up^11^.

**Figure 2:**
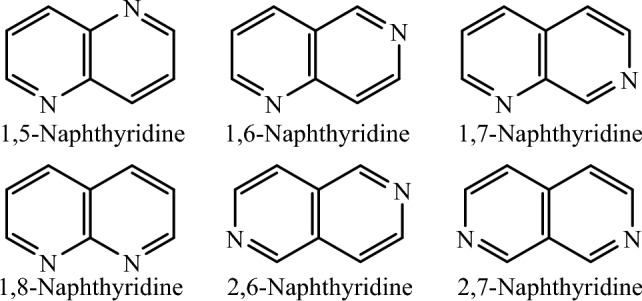
Types of naphthyridines.

Naphthyridines, also called diazanaphthalenes or pyridopyridines, are heterocycles with two fused pyridine rings in which the positions of nitrogens are different. Naphthyridines are categorized into six types based on the arrangements of nitrogen atoms in each ring (Fig. 2)^12,13^.

**Figure 3:**
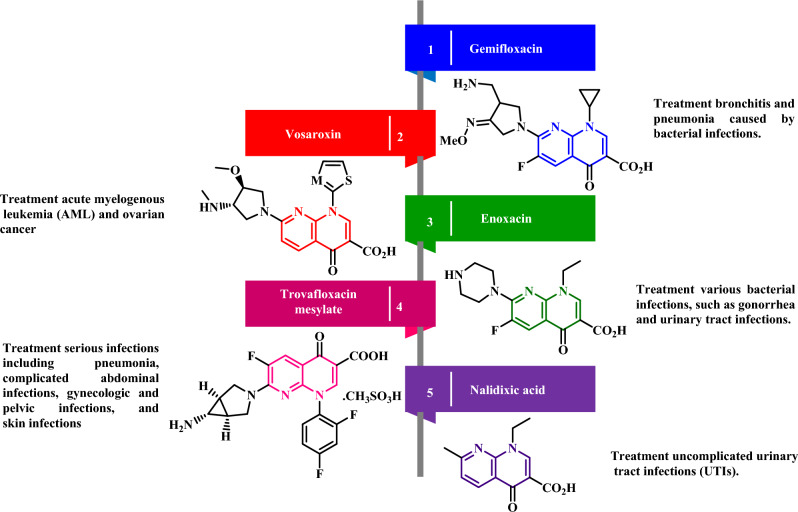
Some drugs contain a 1,8-naphthyridine nucleus.

In recent decades, the synthesis of naphthyridines has attracted significant interest due to their biological activities (such as antimicrobial, anticancer, anti-oxidant, DNA gyrase inhibitors, nemathocides, protein kinase inhibitors, etc.) as a privileged structural motif in organic chemistry^14–17^. Among them, the 1,8-naphthyridines nucleus constitutes the main core of diverse pharmaceutical drugs such as Gemifloxacin, Vosaroxin, Enoxacin, Trovafloxacin mesylate, Nalidixic acid, etc., as demonstrated in Fig. 3^18^.

Various literature has presented different types of modified methods for their synthesis under improved conditions using available starting material (Table 1)^19–24^.

**Table 1:** Reported literature for synthesis of 1,8-naphthyridines.

References	Condition	Time (min)	Yield (%)
^19^	Piperidine, microwave irradiation	555–675	74–84
^20^	AlCl_3_, ClCH_2_CH_2_Cl, MW irradiation (method A) TfOSiMe_3_, AcOEt (method B)	85 (method A) 270 (method B)	30–98
^21^	Thiamine hydrochloride, H_2_O, 90 °C	120–150	93–94
^22^	TBBDA or PBBS, MeCN, r.t	180–720	70–87
^23^	Er/IDA/CPTMS@CoFe2O4, H_2_O, 80 °C	90–150	91–94
^24^	PTBSA-SO_3_H, H_2_O:EtOH (1:1.2 mL), r.t.	30–90	82–96

**Figure 4:**
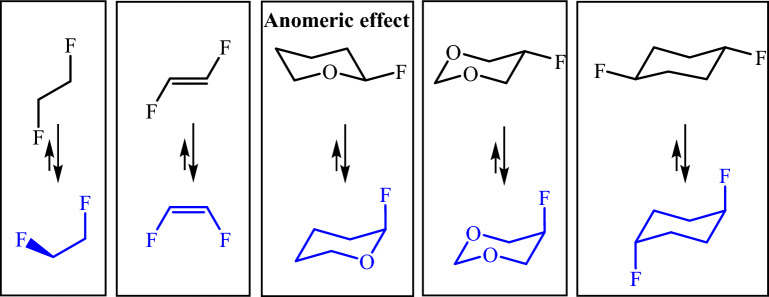
Examples of the influence of the stereo-electronic effect.

Control reactivity and selectivity is a continuous challenge in organic chemistry. Stereo-electronic control is one of the main concepts in this field that explains the mechanism of some organic reactions. Stereo-electronic effects are based on the quantum nature of molecular bonds, which represent a structure–reactivity relationship. The anomeric effect (AE) is a known stereo-electronic interaction in carbohydrate chemistry that Lemieux and Chu first introduced to explain the adoption of an axial configuration in glucose derivatives having an electronegative substitution at the anomeric carbon. Similar conformational preferences are observed for some six-membered heterocycle compounds. Figure 4 shows how stereo-electronic effects affect the stability of conformers and isomers^25–28^.

**Figure 5:**
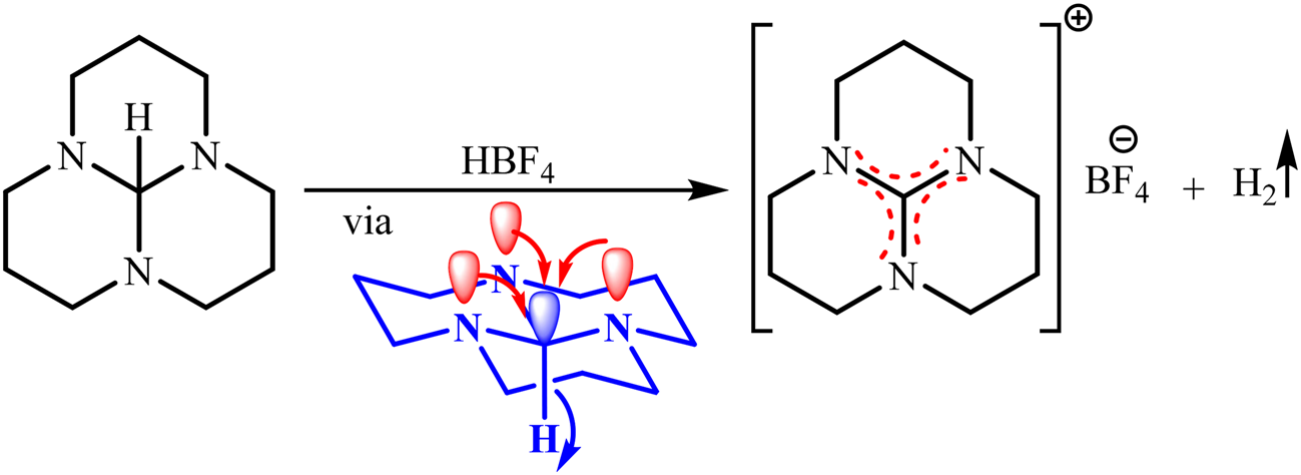
Reaction of tricyclic orthoamide with HBF_4_ under the influence of the anomeric effect.

The anomeric effect (negative hyperconjugation) can lead to the generation of final products through sharing non-bonded lone pairs electrons of heteroatom into the anti-bonding orbitals of the C-H bond without employing an oxidation agent^29,30^. The ability of the anomeric effect in the mechanical aspects of the reaction can be observed in the tricyclic orthoamide, which leads to the release of hydrogen by unusual hydride transfer (Fig. 5)^31^.

**Figure 6:**
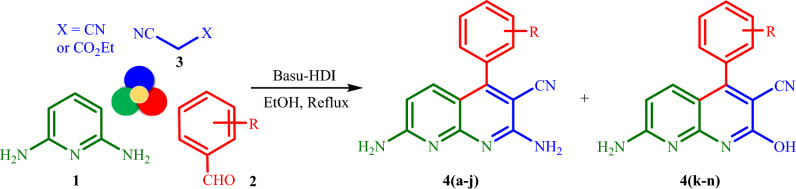
Synthesis of 4(a–n) catalyzed by Basu-HDI.

According to Table 1, previous reports have disadvantages such as long reaction time, relatively low efficiency, low thermal stability of the catalyst, and difficult recovery. Therefore, in continuation of our last study on the synthesis and applications of the urea-based catalysts (the Basu-framework)^32,33^, we decided to synthesize Basu-HDI as a novel heterogeneous catalyst. Then, it was applied to the synthesis of pharmacologically important 1,8-naphthyridines **4(a–n)** via a three-component reaction of 2,6-diaminopyridine **1**, aromatic aldehydes **2**, and malononitrile/ethyl cyanoacetate **3** under reflux condition (Fig. 6). The corresponding products **4(a–n)** were obtained in high yields following a one‐pot tandem Knoevenagel/Michael/cyclization/anomeric-based oxidation reaction.

## Experimental

All chemicals were purchased from the Merck or Aldrich chemical companies and employed without further purification. The reaction progression and purity of the synthesized compounds were checked by TLC (silica gel 60 F-254 plates). The FT‐IR spectra were taken on a Perkin Elmer Spectrum Version 10.02.00 employing KBr pellets. The ^1^H NMR (400 MHz) and ^13^C NMR (100 MHz) spectra were recorded on a Bruker spectrometer (δ in ppm) by DMSO‐*d*_*6*_ as a solvent with chemical shifts measured relative to TMS as the internal standard. The mass spectra were taken on the Agilent Mass Spectrometer (HP), Model: 5973 Network Mass Selective Detector, Ion source: Electron Impact (EI) 70 eV, Ion source temperature: 230 °C, with Quadrupole Analyzer. Melting points were measured with a BUCHI 510 melting point apparatus. Elemental analysis was carried out using an MIRA II analyser. A MIRA III analyzer took the FESEM images. The X-ray diffraction (XRD) measurements were performed with an XRD Philips PW1730. Thermo-gravimetric-differential thermal analysis (TGA-DTA) was accomplished using an SDT-Q600 device.

### The general strategy for the construction of the Basu-HDI framework

The synthesis would start with the initial preparation of the Basu framework using the hydro-solvothermal method based on the previously reported protocol^32^. Briefly, 4-pyridinecarb-aldehyde (20 mmol) and *p*-phenylenediamine (10 mmol) were initially reacted in the presence of piperidine (0.1 mL) as a catalyst under refluxing toluene, yielding BDA4BPy ligand in good yield. Then, ZrCl_4_ (1.2 mmol), BDA4BPy ligand (0.3 mmol), and 2-aminoterephthalic acid (BDC-NH_2_) (0.5 mmol) were dissolved in DMF (140 mL), dispersed, and the obtained mixture stirred for 15 min at room temperature, and then acetic acid (20 mL) was added. Next, the mixture was placed in a Teflon reactor and heated in an oven to 120 °C for 24 h. After cooling, the resulting mixture was centrifuged and washed with DMF and ethanol to provide the Basu framework.

**Figure 7:**
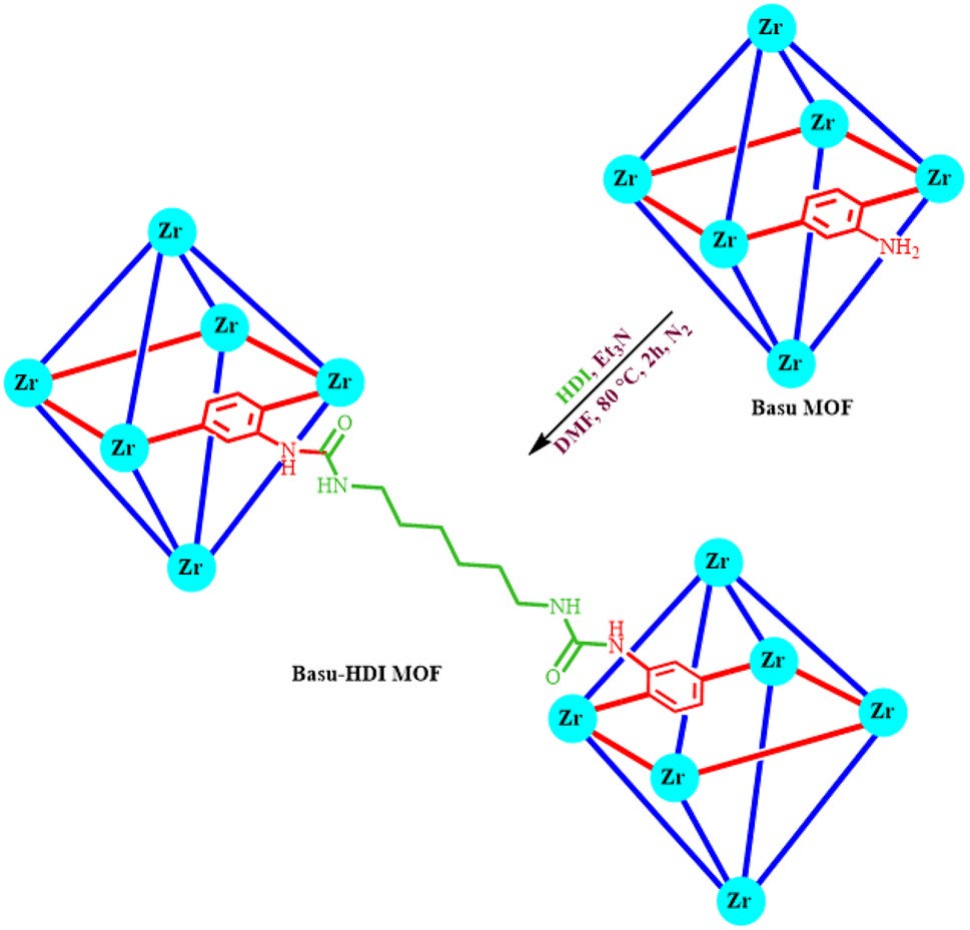
Synthesis of Basu-HDI framework.

Subsequently, a mixture of the prepared Basu framework (250.0 mg) and hexamethylene diisocyanate (HDI) (5.0 mL) was stirred in the presence of the triethylamine (8.7 mL) in DMF (25.0 mL) for 2 h under nitrogen atmosphere at 80 °C (Fig. 7). After cooling the reaction mixture, the resulting solid was washed with DMF (2 × 10) and ethanol (3 × 10) and dried in an oven at 80 °C^34,35^. FT-IR, EDX, elemental mapping analysis, XRD, SEM, BET, and TGA-DTA analysis evaluated the synthesized Basu-HDI MOF.

### General procedure for the synthesis of 4(a–n) catalyzed by Basu-HDI

2,6-Diaminopyridine (1.0 mmol), malononitrile/ethyl cyanoacetate (1.0 mmol), aromatic aldehyde (1.0 mmol), Basu-HDI (20.0 mg) as a heterogeneous catalyst and ethanol (5.0 mL) were mixed and stirred under reflux conditions for the appropriate time. After the reaction (TLC, n-hexane/ethyl acetate) was completed, the reaction mixture was diluted with DMF (6.0 mL) to dissolve the organic products. After separating the catalyst by centrifugation and evaporating the solvent, the **4(a–n)** products were purified by washing them with methanol and confirming their structure using FT‐IR, NMR, and mass spectrometry techniques.

### Spectral data

**2,7-Diamino-4-(3-hydroxyphenyl)-1,8-naphthyridine-3-carbonitrile (4a)** Cream solid, M.p. > 350 °C, IR (KBr, cm^–1^): ν = 3513, 3410, 3324, 3197, 2213, 1644 and 1619. ^1^H NMR (400 MHz, DMSO-*d*_*6*_) δ = 9.80 (s, 1H), 7.36 (t, *J* = 7.8 Hz, 1H), 7.26 (d, *J* = 8.9 Hz, 1H), 6.99 (s, 2H), 6.93 (dd, *J* = 8.1, 2.4 Hz, 1H), 6.80 (s, 2H), 6.78 (s, 1H), 6.75 (d, *J* = 2.1 Hz, 1H), 6.44 (d, *J* = 8.9 Hz, 1H). ^13^C NMR (100 MHz, DMSO-*d*_*6*_) δ = 162.3, 158.9, 158.5, 157.2, 155.0, 135.8, 135.6, 129.7, 119.5, 116.8, 116.0, 115.7, 109.3, 108.2, 87.3. ESI-mass: m/z = 277.

**2,7-Diamino-4-phenyl-1,8-naphthyridine-3-carbonitrile (4b)** Yellow pall solid, M.p. > 350 °C, IR (KBr, cm^–1^): ν = 3431, 3320, 3148, 2205, 1607 and 1561. ^1^H NMR (400 MHz, DMSO-*d*_*6*_) δ = 7.63–7.51 (m, 3H), 7.42 (dd, *J* = 7.3, 2.3 Hz, 2H), 7.21 (d, *J* = 8.9 Hz, 1H), 7.01 (s, 2H), 6.84 (s, 2H), 6.44 (d, *J* = 8.9 Hz, 1H). ^13^C NMR (100 MHz, DMSO-*d*_*6*_) δ = 162.3, 158.9, 158.6, 155.0, 135.5, 134.7, 129.1, 129.0, 128.5, 116.8, 109.4, 108.2, 87.5. ESI-mass: m/z = 261.

**2,7-Diamino-4-(p-tolyl)-1,8-naphthyridine-3-carbonitrile (4c)** Yellow solid, M.p. 331–33 °C, IR (KBr, cm^–1^): ν = 3495, 3419, 3389, 3287, 3166, 2207, 1639, 1604 and 1499. ^1^H NMR (400 MHz, DMSO-*d*_*6*_) δ = 7.38 (d, *J* = 7.8 Hz, 2H), 7.31 (d, *J* = 7.9 Hz, 2H), 7.24 (d, *J* = 9.0 Hz, 1H), 6.99 (s, 2H), 6.81 (s, 2H), 6.44 (d, *J* = 8.9 Hz, 1H), 2.42 (s, 3H). ^13^C NMR (100 MHz, DMSO-*d*_*6*_) δ = 162.2, 158.9, 158.6, 155.1, 138.7, 135.6, 131.7, 129.1, 129.0, 116.9, 109.3, 108.3, 87.5, 20.8. ESI-mass: m/z = 275.

**2,7-Diamino-4-(4-isopropylphenyl)-1,8-naphthyridine-3-carbonitrile (4d)** Yellow solid, M.p. > 350 °C, IR (KBr, cm^–1^): ν = 3492, 3429, 3385, 3281, 317, 2958, 2206, 1636, 1603, and 1574. ^1^H NMR (250 MHz, DMSO-*d*_*6*_) δ = 7.42 (d, *J* = 7.6 Hz, 2H), 7.34 (s, 2H), 7.22 (d, *J* = 9.0 Hz, 1H), 6.95 (s, 2H), 6.77 (s, 2H), 6.42 (d, *J* = 9.2 Hz, 1H), 3.05–2.90 (m, 1H), 1.26 (d, *J* = 6.2 Hz, 6H). ^13^C NMR (62.5 MHz, DMSO-*d*_*6*_) δ = 162.76, 159.44, 159.08, 155.47, 149.83, 136.10, 132.51, 129.59, 126.97, 117.48, 109.84, 108.81, 87.95, 33.68, 24.16. ESI-mass: m/z = 303.

**2,7-Diamino-4-(4-methoxyphenyl)-1,8-naphthyridine-3-carbonitrile (4e)** Yellow pall solid, M.p. 331–333 °C decompose, IR (KBr, cm^–1^): ν = 3500, 3426, 3387, 3273, 3162, 2207, 1605 and 1501. ^1^H NMR (400 MHz, DMSO-*d*_*6*_) δ = 7.37 (d, *J* = 8.7 Hz, 2H), 7.28 (d, *J* = 8.9 Hz, 1H), 7.16–7.10 (m, 2H), 6.99 (s, 2H), 6.80 (s, 2H), 6.44 (d, *J* = 8.9 Hz, 1H), 3.86 (s, 3H). ^13^C NMR (100 MHz, DMSO-*d*_*6*_) δ = 162.2, 159.8, 158.9, 158.6, 154.8, 135.6, 130.6, 126.6, 117.1, 113.9, 109.2, 108.4, 87.6, 55.2, 40.0. ESI-mass: m/z = 291.

**2,7-Diamino-4-(3,4-dimethoxyphenyl)-1,8-naphthyridine-3-carbonitrile (4f)** Yellow solid, M.p. 303 °C, IR (KBr, cm^–1^): ν = 3499, 3413, 3387, 3278, 3185, 2829, 2207, 1605, and 1565. ^1^H NMR (400 MHz, DMSO-*d*_*6*_) δ = 7.35 (d, *J* = 8.9 Hz, 1H), 7.14 (d, *J* = 8.3 Hz, 1H), 7.03 (d, *J* = 2.0 Hz, 1H), 7.01–6.92 (m, 3H), 6.79 (s, 2H), 6.45 (d, *J* = 8.9 Hz, 1H), 3.85 (s, 3H), 3.78 (s, 3H). ^13^C NMR (100 MHz, DMSO-*d*_*6*_) δ = 162.24, 158.95, 158.62, 154.98, 149.37, 148.43, 135.85, 126.83, 121.76, 117.13, 112.84, 111.54, 109.27, 108.48, 87.70, 55.64, 55.51. ESI-mass: m/z = 321.

**2,7-Diamino-4-(4-bromophenyl)-1,8-naphthyridine-3-carbonitrile (4g)** Yellow solid, M.p. > 350 °C, IR (KBr, cm^–1^): ν = 3437, 3342, 3203, 2208, 1668, 1634 and 1613. ^1^H NMR (400 MHz, DMSO-*d*_*6*_) δ = 7.78 (d, *J* = 8.2 Hz, 2H), 7.40 (d, *J* = 8.2 Hz, 2H), 7.20 (d, *J* = 8.9 Hz, 1H), 7.04 (s, 2H), 6.88 (s, 2H), 6.45 (d, *J* = 9.0 Hz, 1H). ^13^C NMR (100 MHz, DMSO-*d*_*6*_) δ = 162.3, 158.8, 158.6, 153.7, 135.4, 133.9, 131.6, 131.2, 122.7, 116.7, 109.6, 108.1, 87.3. ESI-mass: m/z = 339.

**2,7-Diamino-4-(4-chlorophenyl)-1,8-naphthyridine-3-carbonitrile (4h)** Yellow solid, M.p. 338 °C decompose, IR (KBr, cm^–1^): ν = 3436, 3341, 3239, 3202, 2209, 1642, 1612 and 1515. ^1^H NMR (400 MHz, DMSO-*d*_*6*_) δ = 7.65 (d, *J* = 8.5 Hz, 2H), 7.47 (d, *J* = 8.4 Hz, 2H), 7.20 (d, *J* = 8.9 Hz, 1H), 7.04 (s, 2H), 6.88 (s, 2H), 6.45 (d, *J* = 9.0 Hz, 1H). ^13^C NMR (100 MHz, DMSO-*d*_*6*_) δ = 162.3, 158.8, 158.6, 153.7, 135.4, 134.0, 133.5, 131.0, 128.7, 116.7, 109.6, 108.1, 87.4. ESI-mass: m/z = 295.

**2,7-Diamino-4-(2-fluorophenyl)-1,8-naphthyridine-3-carbonitrile (4i)** Pail yellow solid, M.p. 318–321 °C, IR (KBr, cm^–1^): ν = 3434, 3347, 3239, 3139, 2808, 2214, 1645, 1565. ^1^H NMR (400 MHz, DMSO-*d*_*6*_) δ = 7.53–7.48 (m, 2H), 7.42 (t, *J* = 8.9 Hz, 2H), 7.22 (d, *J* = 8.9 Hz, 1H), 7.05 (s, 2H), 6.89 (s, 2H), 6.47 (d, *J* = 9.0 Hz, 1H). ^13^C NMR (100 MHz, DMSO-*d*_*6*_) δ = 163.7, 162.4, 161.3, 158.9, 158.6, 154.0, 135.5, 131.5, 131.4, 131.0, 131.0, 116.8, 115.7, 115.5, 109.5, 108.4, 87.7, 48.6.

**2,7-Diamino-4-(2,4-dichlorophenyl)-1,8-naphthyridine-3-carbonitrile (4j)** Yellow solid, M.p. > 350 °C, IR (KBr, cm^–1^): ν = 3441, 3350, 3182, 2214, 1639, 1610 and 1570. ^1^H NMR (400 MHz, DMSO-*d*_*6*_) δ = 7.92 (d, *J* = 2.1 Hz, 1H), 7.65 (dd, *J* = 8.2, 2.1 Hz, 1H), 7.52 (d, J = 8.2 Hz, 1H), 7.16–6.91 (m, 5H), 6.45 (d, *J* = 8.9 Hz, 1H). ^13^C NMR (100 MHz, DMSO-*d*_*6*_) δ = 162.5, 158.7, 158.6, 151.0, 134.9, 134.8, 132.9, 132.7, 132.2, 129.3, 127.9, 116.1, 109.9, 108.1, 87.7. ESI-mass: m/z = 329.

**7-Amino-2-hydroxy-4-(3-nitrophenyl)-1,8-naphthyridine-3-carbonitrile (4k)** Ceram solid, M.p. > 350 °C, IR (KBr, cm^–1^): ν = 3397, 3174, 3086, 2938, 2227, 1615, 1536 and 1375. ^1^H NMR (400 MHz, DMSO-*d*_*6*_) δ = 12.37 (s, 1H), 8.43 (d, *J* = 8.6 Hz, 1H), 8.34 (s, 1H), 7.96 (d, *J* = 8.1 Hz, 1H), 7.90 (t, *J* = 7.9 Hz, 1H), 7.48 (s, 2H), 7.15 (d, *J* = 9.0 Hz, 1H), 6.38 (d, *J* = 9.0 Hz, 1H). ^13^C NMR (100 MHz, DMSO-*d*_*6*_) δ = 162.1, 160.3, 156.2, 152.1, 147.8, 136.4, 135.5, 135.2, 130.5, 124.4, 123.5, 116.2, 107.5, 104.4, 97.8. ESI-mass: m/z = 307.

**7-Amino-2-hydroxy-4-(3-hydroxyphenyl)-1,8-naphthyridine-3-carbonitrile (4l)** Ceram solid, M.p. > 350 °C, IR (KBr, cm^–1^): ν = 3404, 3188, 2945, 2233, 1618, 1481, 1379 and 1116. ^1^H NMR (400 MHz, DMSO-*d*_*6*_) δ = 12.23 (s, 1H), 9.84 (s, 1H), 7.37 (t, *J* = 8.9 Hz, 3H), 7.18 (d, *J* = 8.9 Hz, 1H), 6.94 (dd, *J* = 8.2, 1.5 Hz, 1H), 6.84–6.76 (m, 2H), 6.38 (d, *J* = 9.0 Hz, 1H). ^13^C NMR (100 MHz, DMSO-*d*_*6*_) δ = 162.0, 160.5, 158.8, 157.3, 152.0, 136.6, 135.1, 129.9, 118.9, 116.4, 116.3, 115.1, 107.2, 104.3, 97.3. ESI-mass: m/z = 278.

**7-Amino-4-(3-ethoxy-4-hydroxyphenyl)-2-hydroxy-3,4-dihydro-1,8-naphthyridine-3-carbonitrile (4m)** Ceram solid, M.p. > 350 °C, IR (KBr, cm^–1^): ν = 3469, 3351, 3236, 2976, 2256, 2219, 1684, 1629, 1520, 1473, 1355 and 1042. ^1^H NMR (400 MHz, DMSO-*d*_*6*_) δ = 10.69 (d, *J* = 9.2 Hz, 1H), 8.98 (d, *J* = 15.2 Hz, 1H), 6.96–6.91 (m, 1H), 6.83–6.76 (m, 1H), 6.74–6.69 (m, 1H), 6.59–6.48 (m, 1H), 6.12–5.93 (m, 3H), 4.81 (d, *J* = 12.9 Hz, 1H), 4.37–4.29 (m, 1H), 4.02–3.92 (m, 2H), 1.31 (t, *J* = 7.0 Hz, 3H). ^13^C NMR (100 MHz, DMSO-*d*_*6*_) δ = 163.6, 158.3, 148.0, 146.9, 146.3, 137.9, 137.1, 130.3, 128.9, 121.2, 119.5, 117.0, 116.6, 115.6, 115.5, 113.5, 106.9, 106.4, 102.8, 102.4, 63.7, 42.9, 41.8, 14.6. ESI-mass: m/z = 324.

**7-Amino-4-(4-chlorophenyl)-2-hydroxy-3,4-dihydro-1,8-naphthyridine-3-carbonitrile (4n)** Yellow solid, M.p. > 350 °C, IR (KBr, cm^–1^): ν = 3395, 3344, 3163, 2223, 1623, 1477, 1378 and 1092. ^1^H NMR (250 MHz, DMSO-*d*_*6*_) δ = 10.72 (s, 1H), 7.32 (t, *J* = 39.2 Hz, 5H), 6.42 (s, 1H), 5.98 (s, 2H), 4.90 (d, *J* = 37.3 Hz, 1H), 4.47 (s, 1H). ^13^C NMR (62.5 MHz, DMSO-*d*_*6*_) δ = 163.7, 163.4, 159.2, 158.9, 148.6, 139.0, 138.5, 138.0, 137.4, 132.9, 131.0, 129.8, 129.4, 117.2, 116.8, 106.4, 103.5, 102.9, 43.0, 41.8. ESI-mass: m/z = 298.

### Reusability of the Basu-HDI framework

The recyclability and the reusability of the Basu-HDI catalyst was tested in the synthesis of 2,7-diamino-4-(3-hydroxyphenyl)-1,8-naphthyridine-3-carbonitrile (**4a**) under the mentioned optimal conditions in successive catalytic runs. At the end of each cycle, the catalyst was separated from the reaction mixture, washed several times with ethanol and acetone, air-dried, and used again in the next run.

## Results and discussion

### Characterization of Basu-HDI framework

After characterization of the Basu-MOF with FT‐IR, ^13^C NMR, EDX, elemental mapping, XRD, FESEM, TGA/DTA, and BET analysis^32^, we decided to introduce a new heterogeneous catalyst, namely Basu-HDI, containing the urea groups as an efficient, recyclable, and stable catalyst. Then, the prepared catalyst was characterized by the abovementioned techniques and its catalytic activity was studied in synthesizing diverse **4(a–n)** under reflux conditions.

### Characterization by FT‐IR

**Figure 8:**
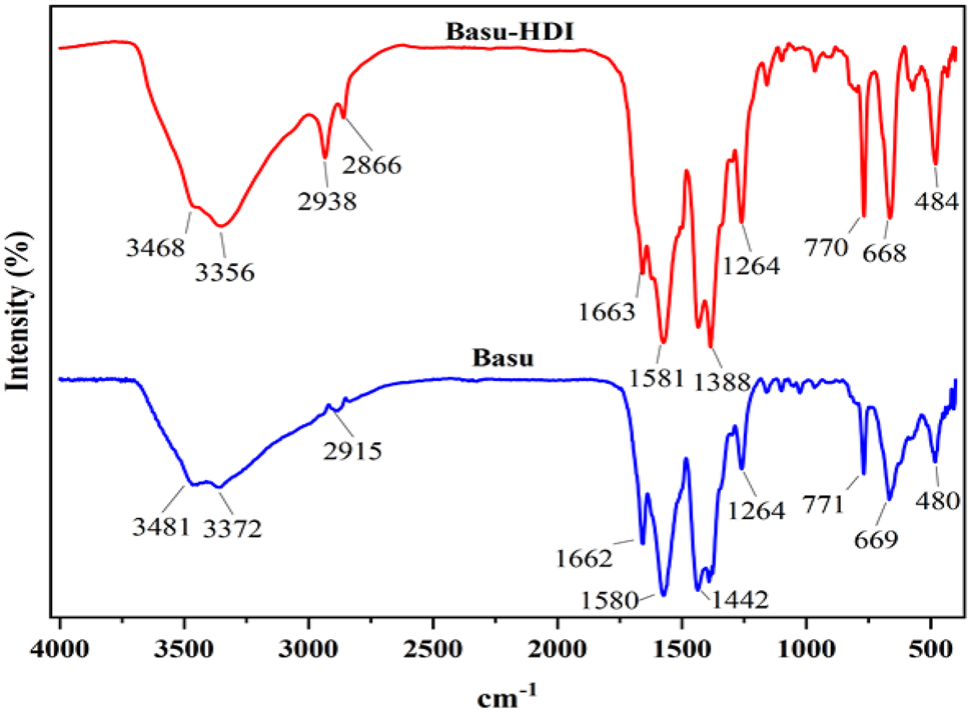
The comparison of FT-IR spectra of Basu and Basu-HDI frameworks.

Comparing the FT-IR spectra of Basu and Basu-HDI shows the successful synthesis of Basu-HDI, as seen in Fig. 8. In the FT-IR spectrum of Basu, absorption bands appeared at 3481, 3372, 1662, 1580, 1442, 771, and 669 cm^−1^ belong to symmetric and asymmetric vibrations of amine groups, stretching vibrations of C=O, C–C aromatic, C–N aromatic, Zr–O–Zr and Zr–O bonds respectively. Basu-HDI absorption bands are similar to Basu with a slight shift. Peaks 2938 and 2866 cm^−1^ are related to aromatic and aliphatic C–H. In addition, the absence of the isocyanate group peak at 2274 cm^−1^ represents that isocyanate groups have successfully reacted with amine groups.

### Characterization by EDX and elemental mapping analysis

**Figure 9:**
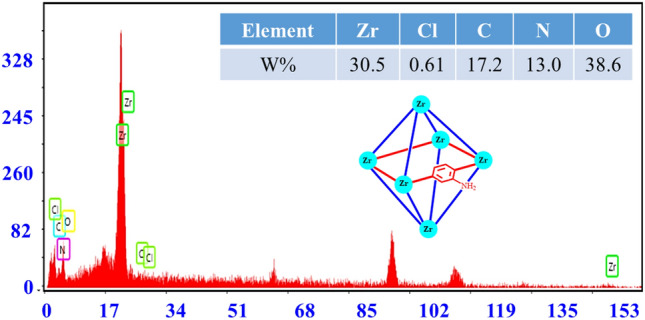
EDX analysis of the Basu framework.

**Figure 10:**
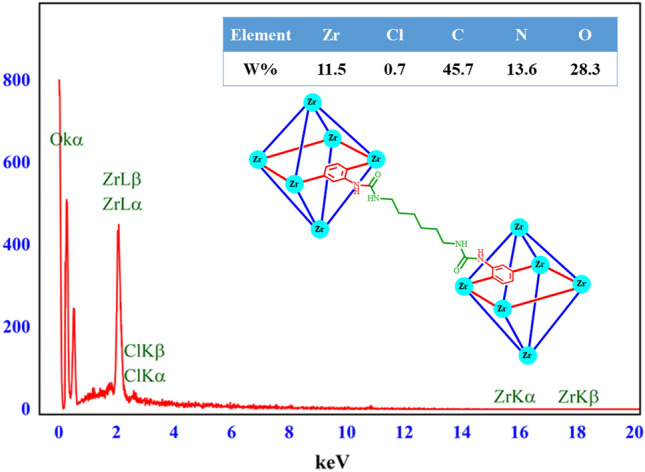
EDX analysis of the Basu-HDI framework.

The EDX technique investigated the chemical structure of Basu and Basu-HDI frameworks. According to the results, the presence of zirconium, chloride, carbon, nitrogen, and oxygen elements confirm the successful synthesis Basu and Basu-HDI frameworks (Figs. 9 and 10).

**Figure 11:**
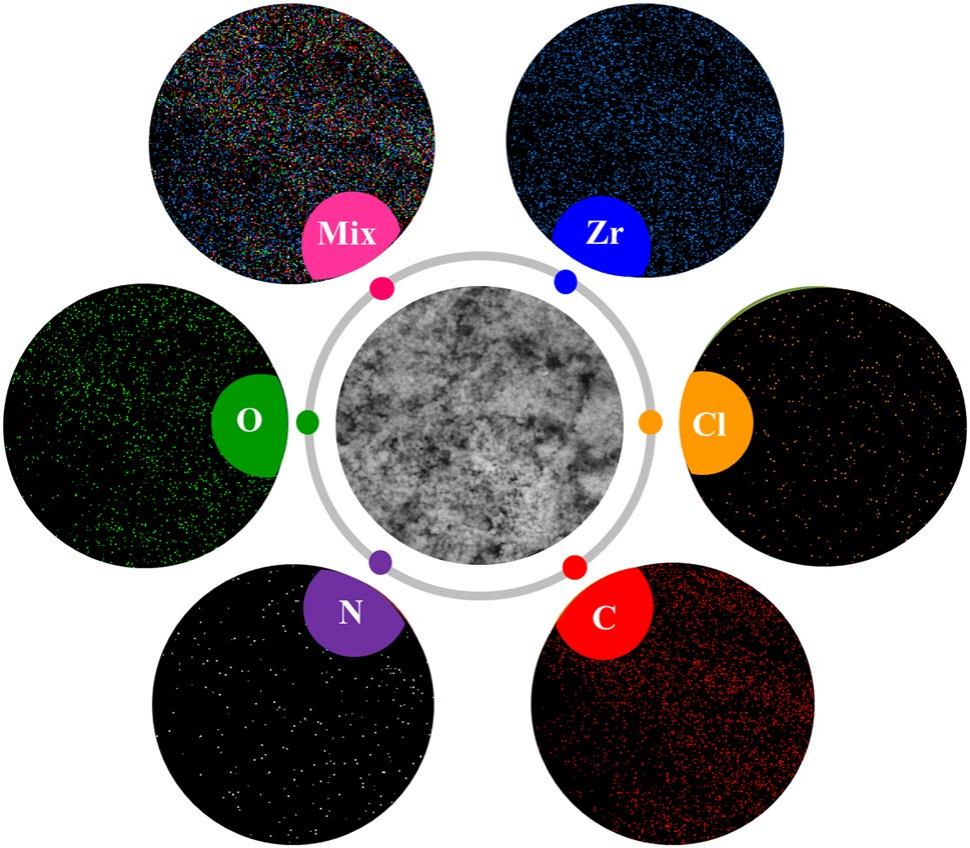
Elemental X-ray mapping analysis of the Basu-HDI framework.

In addition, the elemental X-ray mapping was performed to confirm the results obtained from the EDX analysis of Basu-HDI and study the homogeneous distribution of elements. Figure 11 shows the uniform dispersion of the indicated elements (Zr, Cl, C, N, and O) in the Basu-HDI framework.

### Characterization by the FESEM analysis

**Figure 12:**
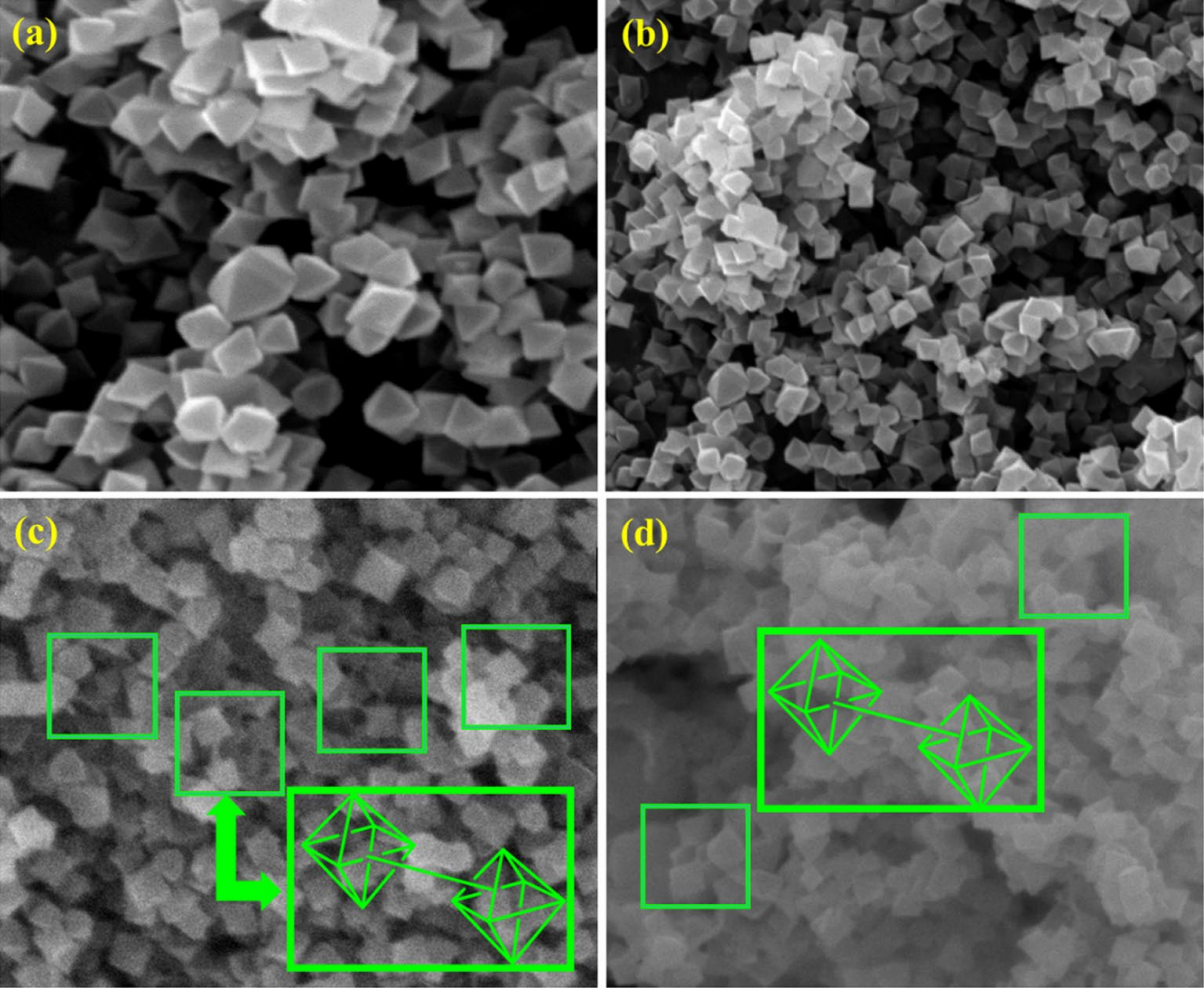
The SEM images of the Basu and Basu-HDI.

Moreover, we used field-emission scanning electron microscopy (FESEM) to examine the morphology of the modified framework. The primary framework possesses an octahedron structure, uniform size, and homogeneous particle distribution (Fig. 12a, b). The FESEM images Basu-HDI show that the Basu morphology was well preserved after modification with hexa-methylene diisocyanate (Fig. 12c, d). As seen, the octahedral structures were connected through a chain, which shows that the functionalization was well done.

### Characterization by PXRD

**Figure 13:**
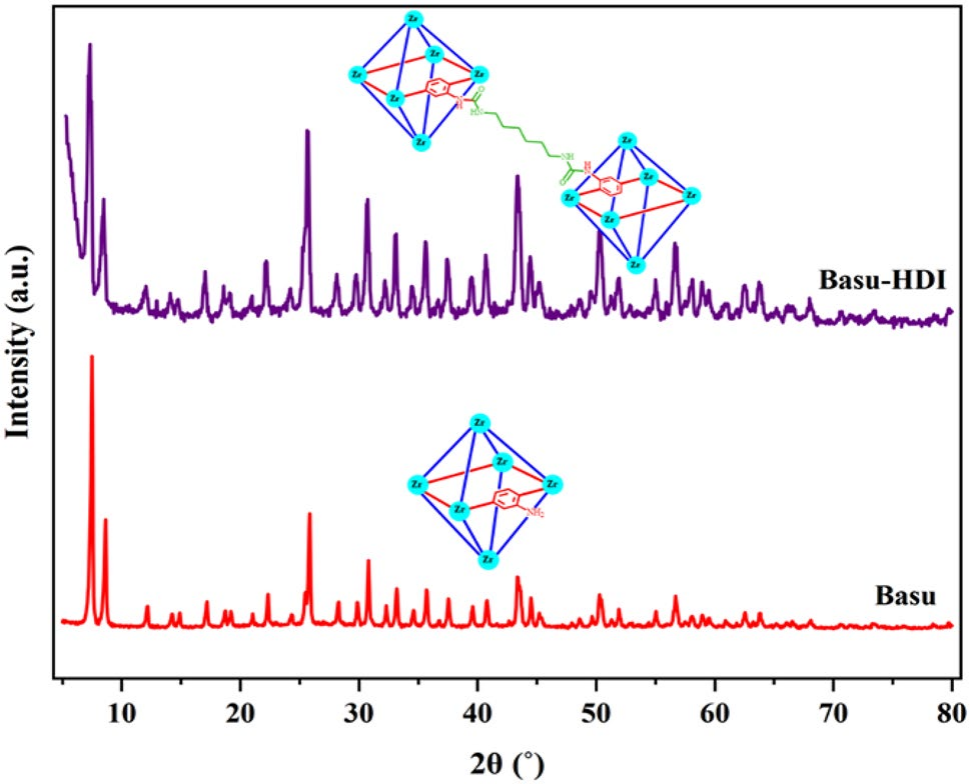
The XRD patterns of Basu and Basu-HDI.

A comparative study applied the PXRD analysis to evaluate the crystalline nature of Basu and Basu-HDI frameworks. As observed in Fig. 13, the Basu-HDI crystal pattern is similar to Basu, with the difference that its index peaks have shifted to a lower degree. Basu and Basu-HDI index peaks have appeared at 2θ = 7.5, 8.6, 25.8, 30.8, and 43.4 and 2θ = 7.3, 8.4, 25.6, 30.6, and 43.3, respectively. The average crystallite size of Basu-HDI is about 16.6 nm based on the Scherrer equation (D = Kλ/(β.cos θ).

### Characterization by BET

**Figure 14:**
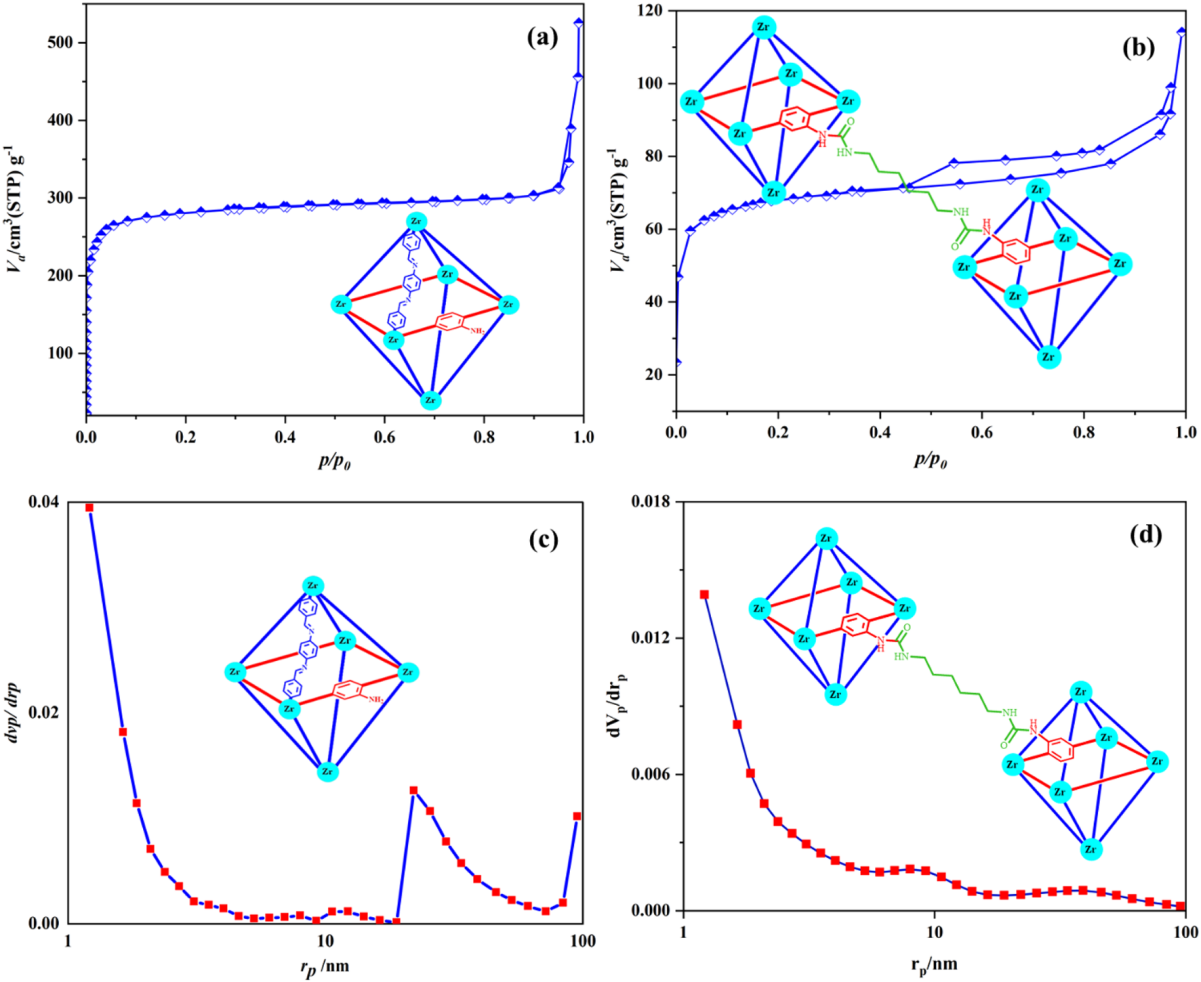
The N_2_ adsorption–desorption curve (BET) of (**a**) Basu and (**b**) Basu-HDI and the BJH adsorption curve of (**c**) Basu and (**d**) Basu-HDI.

To study the porous nature and surface area of Basu-HDI, the N_2_ adsorption–desorption analysis was applied, and its results were compared with the Basu data. As seen in Fig. 14a, b, the Basu isotherm is type I(b), but the Basu-HDI isotherm is type IV based on the IUPAC classification of adsorption isotherms^36^.

In addition, the pore size distribution of Basu and Basu-HDI is 2.9 and 2.7 nm, respectively, according to the BJH adsorption curve (Fig. 14c, d).

According to Table 2, the Basu framework’s surface area and total pore volume are 1107 m^2^ g^−1^ and 0.8 cm^3^ g^−1^, whereas BET’s surface area and total pore volume of Basu-HDI are 256.2 and 0.1, respectively. The reduction of the surface area and pore volumes can be assigned to the decreased free space available in the pores due to modification with hexamethylene diisocyanate.

**Table 2:** Results from the BET analysis of the Basu and Basu-HDI.

Parameter	Basu	Basu-HDI
a_s_ (m^2^ g^−1^)	1107.1	256.2
V_m_ (cm^3^ g^−1^)	254.3	58.8
Total pore volume (cm^3^ g^−1^)	0.8	0.1
Mean pore diameter (nm)	2.9	2.7

### Characterization by TGA-DTA

**Figure 15:**
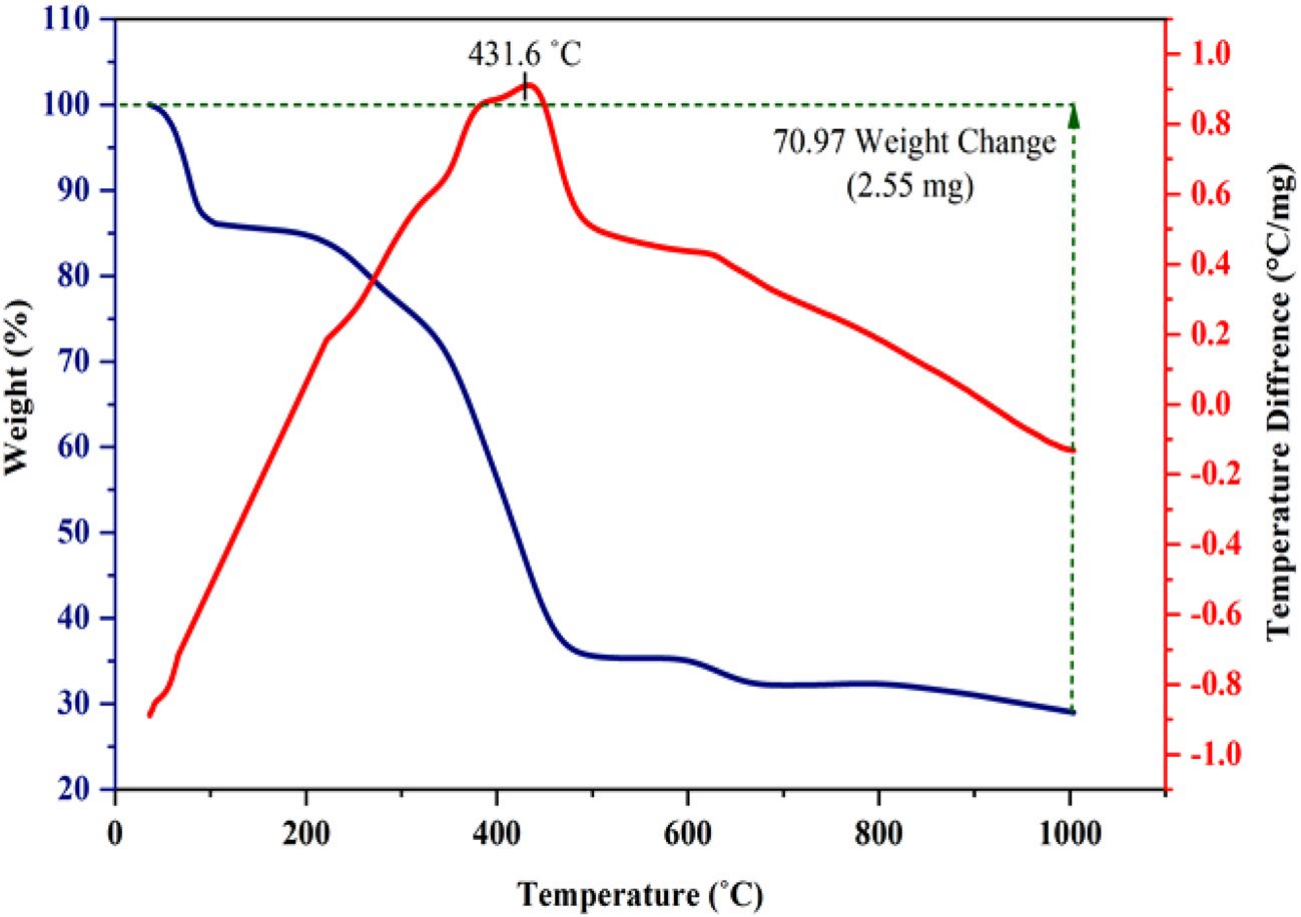
The TGA-DTA diagram of Basu-HDI.

In another research, we used the thermogravimetric–differential thermal (TGA-DTA) technique to measure the stability of the presented catalyst. The results of this study can be seen in Fig. 15. According to the TGA-DTA diagram, Basu-HDI displays a three-step weight loss. The weight loss (21%) observed at 0–270 °C is due to the removal of solvent molecules in the pores and molecules that have been physically absorbed. The following weight loss (44%) at 270–495 °C can be attributed to the separation of HDI from the structure of the catalyst. The third weight loss (6%) at the temperature of 495–1000 °C is probably related to the destruction of the framework [29]. The prepared catalyst is stable up to a temperature of 431 °C. In addition, 29% of the initial mass remains at 1000 °C.

### Optimization of the reaction conditions

After studying the structure and physical properties of the presented catalyst, the catalytic activity of Basu-HDI in the synthesis of pharmacologically valuable **4(a–n)** was perused via a new multicomponent tandem reaction. Initially, the three‐component reaction between 2,6-diamino-pyridine, 3-hydroxybenzaldehydes, and malononitrile was picked out as the model reaction. Then, the effect of different reaction parameters, such as solvent, temperature, and catalyst amount, was evaluated to find the best conditions. It was found (Table 3) that the polar protic solvents (ethanol and methanol) gave higher yields. Although methanol has more polarity than ethanol, its yield is lower due to the lower reflux temperature. An increase in temperature causes an increase in the movement of molecules and, thus, the speed of the reaction. Non-polar solvents such as toluene, CH_2_Cl_2,_ and THF could not perform the reaction due to their inability to dissolve the reagents. Water is less effective than ethanol and methanol due to its low solubility. The presence of polar solvents causes better mixing of reagents; hence, the reaction yield is low under solvent-free conditions due to the lower mixing of reagents.

**Table 3:** Optimization of the reaction conditions for the synthesis of compound **4a.**


Entry	Condition	Catal. Amount (mg)	Time (h)	Yield (%)
**1**	**EtOH, reflux**	**20**	**2**	**90**
2	EtOH:H_2_O (1:1), 80 °C	20	2	64
3	H_2_O, reflux	20	2	40
4	MeOH, reflux	20	20	75
5	CH_3_CN, reflux	20	2	68
6	CO(CH_3_)_2_, reflux	20	2	68
7	Solvent-free, 110 °C	20	2	30
8	Toluene, reflux	20	2	N.R
9	CH_2_Cl_2_, reflux	20	2	N.R
10	THF, reflux	20	2	N.R
11	EtOH, reflux	40	2	82
12	EtOH, reflux	60	2	79
13	EtOH, reflux	–	2	43

Significant values are in [bold].

In addition, increasing the amount of catalyst leads to a decrease in efficiency, possibly due to an increase in the reverse reaction rate. The best result was achieved when the reaction was carried out in the presence of 20 mg of the Basu-HDI under the refluxed ethanol.

To investigate the capability of the proposed catalyst, the model reaction was also performed employing several homogeneous and heterogeneous catalysts under optimal conditions to show that Basu-HDI was more effective than other catalysts (Table 4). Basu-HDI has higher efficiency than previously reported catalyst (Basu-DPU), probably due to having more active sites. Basu-HDI has urea moieties whose NH groups can catalyze the desired reaction through hydrogen bonding.

**Table 4:** Screening the model reaction in the presence of several catalysts.

Entry	Catalyst	Catal. Amount (mg)	Time (h)	Yield (%)
1	Basu-DPU^32^	20	2	75
2	MIL(Cr)/NHEtN(CH_2_PO_3_H_2_)_2_^37^	20	2	68
3	MIL-125(Ti)-N(CH_2_PO_3_H_2_)_2_^38^	20	2	75
4	*p*-TSA	20	2	65
5	L-Proline	20	2	60
6	SSA	20	2	75
7	FSiPSS	20	2	68
8	ATSPTC-Mn^39^	20	2	70

### Synthesis of diverse 4(a–n)

**Figure 16:**
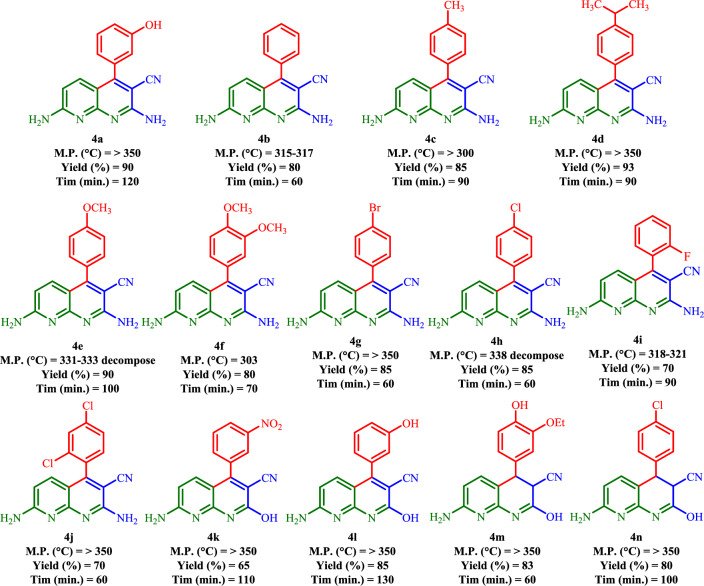
Synthesis of new **4(a–n)** using Basu-HDI. Reaction conditions: 2,6-diaminopyridine (1.0 mmol), malononitrile/ethyl cyanoacetate (1.0 mmol), aromatic aldehyde (1.0 mmol), Basu-HDI (20.0 mg) and ethanol (5.0 mL) under reflux.

To further expand the scope of this synthetic process, the reaction of a wide range of aromatic /heteroaromatic aldehydes and ethyl cyanoacetate was studied under optimal conditions affording **4(b–n)** with high yields (Fig. 16). The electron-rich aldehydes and malononitrile provided the new corresponding **4(b–n)** in high yields, and the obtained products were purified by washing them with ethanol and methanol.

### Proposed mechanism for the synthesis of diverse 4(a–n)

**Figure 17:**
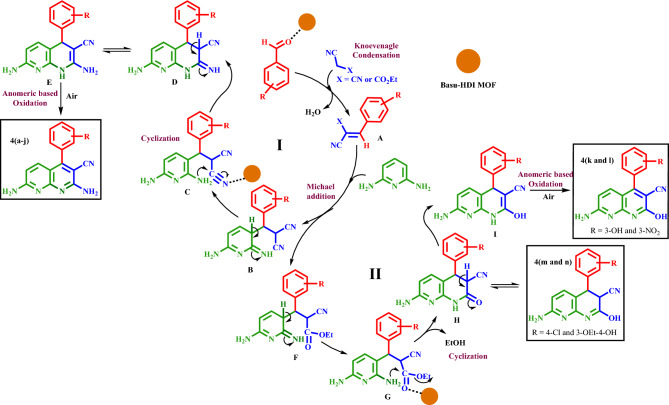
Proposed mechanism for the synthesis of new **4(a–n)** using Basu-HDI.

Figure 17 indicates the suggested mechanism for explaining this transformation. The reaction can proceed in two ways depending on the active methylene used. The proposed mechanism generally starts with activating the aldehyde by the Basu-HDI catalyst, followed by Michael acceptor **A**’s formation via the Knoevenagel aldehyde condensation with the malononitrile. Then, **A** undergoes the nucleophilic attack of 2,6-diaminopyridine, producing intermediate **B** or **F** depending on the active methylene used. If malononitrile is used, intermediate **B** is obtained. **B** can undergo spontaneous cyclization, providing cascade route **I** for synthesizing **4(a–j)** through cyclization/ tautomerization/oxidation sequences. The last step occurs without using an oxidant based on the anomeric effect.

On the other hand, if ethyl cyanoacetate is used, intermediate **F** is generated (route **II**). Then, **F** is converted to the intermediate **G** through intermolecular cyclization with EtOH delivery. **G** readily undergoes tautomerization, providing intermediate **I** and 1,8-naphthyridines **4m** and** 4n**. Finally, without an oxidant and based on the anomeric effect, the new intermediate **I** would undergo oxidation to generate the 1,8-naphthyridines **4k** and** 4l**.

### Reusability of the Basu-HDI framework

**Figure 18:**
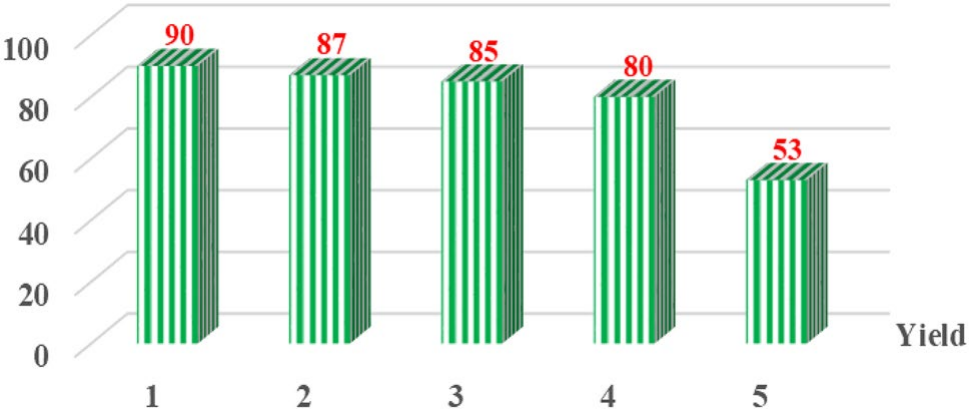
Recyclability of the Basu-HDI catalyst in the synthesis of **4a**.

The use of heterogeneous catalysts is of great economic importance due to the ability to recycle and reuse them consecutively^40,41^. The Reliability of the Basu-HDI framework was studied under optimal conditions. The results illustrated that the proposed Basu-HDI catalyst could be recycled and reused five times without significant change in its catalytic performance (Fig. 18).

**Figure 19:**
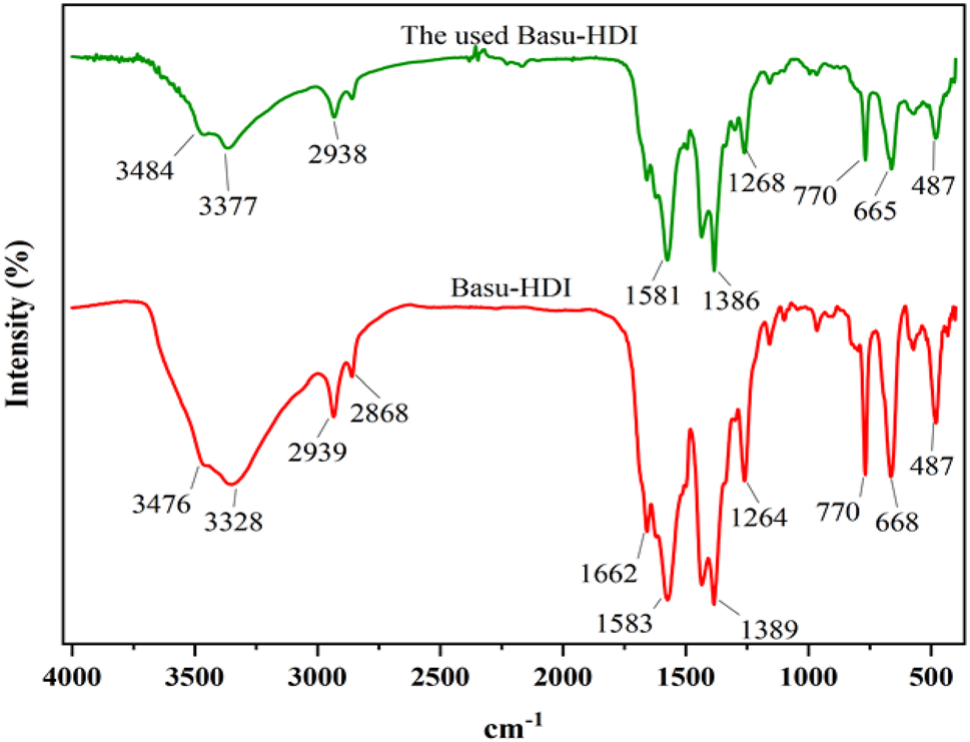
FT-IR spectrum of the reused Basu-HDI in comparison with the fresh catalyst.

In another study, the structure of the used catalyst was characterized by the FT-IR technique after five runs. The FT-IR spectrum of the used Basu-HDI catalyst is similar to the fresh catalyst, indicating the catalyst stability (Fig. 19).

## Conclusion

In conclusion, we report the successful design and synthesis of a new pillar-layered MOF with the urea linkers, Basu-HDI, through modification with hexamethylene diisocyanate. Different analyses confirmed the structure and morphology of the presented catalyst. The FESEM images Basu-HDI show that the octahedral structures were connected through a chain, and the functionalization was nicely performed. Moreover, Basu-HDI was applied as a heterogeneous catalyst in synthesizing a new valuable **4(a–n)** through the Knoevenagel/Michael/cyclization/ oxidation sequence. Some advantages of the Basu-HDI catalyst are low catalyst loading, recovery and reusability, short reaction time, high yields, compatibility with different functional groups, and simple purification. Furthermore, the recycled catalyst’s FT-IR spectrum shows that the catalyst’s structure remains intact.

## Supplementary Information

Supplementary Information.

## Data Availability

All data generated or analysed during this study are included in this published article (and its Supplementary Information files).
