# Supplementary material for: A novel pillar-layered MOF with urea linkers as a capable catalyst for synthesis of new 1,8-naphthyridines via the anomeric-based oxidation
Source: Sci Rep. 2024 Nov 12;14:27727. doi: 10.1038/s41598-024-66539-3 (PMC11557916; doi:10.1038/s41598-024-66539-3)
Supplement: Supplementary file 1 — Supplementary Information. [file 41598_2024_66539_MOESM1_ESM.docx]

**Supporting Information**

**(Scientific Reports)**

**A novel pillar-layered MOF with urea linkers as a capable catalyst for synthesis of new** **1,8-naphthyridines via the anomeric-based oxidation**

**Masoumeh Beiranvand, Davood Habibi*, Hosein Khodakarami**

Department of Organic Chemistry, Faculty of Chemistry and Petroleum Sciences, Bu-Ali Sina University, Hamedan, Iran

*Corresponding author email: [davood.habibi@gmail.com](mailto:davood.habibi@gmail.com) (& dhabibi@basu.ac.ir), Tel: 98 81 38380922; Fax: 98 81 31408025

**Contents Page**

[FT-IR Spectrum of **4a** 1](#_Toc166961286)

[^1^H NMR Spectrum of **4a** 1](#_Toc166961287)

[^13^C NMR Spectrum of **4a** 2](#_Toc166961288)

[MS Spectrum of **4a** 3](#_Toc166961289)

[FT-IR Spectrum of **4b** 4](#_Toc166961290)

[^1^H NMR Spectrum of **4b** 4](#_Toc166961291)

[^13^C NMR Spectrum of **4b** 5](#_Toc166961292)

[MS Spectrum of **4b** 6](#_Toc166961293)

[FT-IR Spectrum of **4c** 6](#_Toc166961294)

[^1^H NMR Spectrum of **4c** 7](#_Toc166961295)

[^13^C NMR Spectrum of **4c** 7](#_Toc166961296)

[MS Spectrum of **4c** 8](#_Toc166961297)

[FT-IR Spectrum of **4d** 8](#_Toc166961298)

[^1^H NMR Spectrum of **4d** 9](#_Toc166961299)

[^13^C NMR Spectrum of **4d** 9](#_Toc166961300)

[MS Spectrum of **4d** 10](#_Toc166961301)

[FT-IR Spectrum of **4e** 10](#_Toc166961302)

[^1^H NMR Spectrum of **4e** 11](#_Toc166961303)

[^13^C NMR Spectrum of **4e** 11](#_Toc166961304)

[MS Spectrum of **4e** 12](#_Toc166961305)

[FT-IR Spectrum of **4f** 12](#_Toc166961306)

[^1^H NMR Spectrum of **4f** 13](#_Toc166961307)

[^13^C NMR Spectrum of **4f** 13](#_Toc166961308)

[MS Spectrum of **4f** 14](#_Toc166961309)

[FT-IR Spectrum of **4g** 14](#_Toc166961310)

[^1^H NMR Spectrum of **4g** 15](#_Toc166961311)

[^13^C NMR Spectrum of **4g** 15](#_Toc166961312)

[MS Spectrum of **4g** 16](#_Toc166961313)

[FT-IR Spectrum of **4h** 16](#_Toc166961314)

[^1^H NMR Spectrum of **4h** 17](#_Toc166961315)

[^13^C NMR Spectrum of **4h** 17](#_Toc166961316)

[MS Spectrum of **4h** 18](#_Toc166961317)

[FT-IR Spectrum of **4i** 18](#_Toc166961318)

[^1^H NMR Spectrum of **4i** 19](#_Toc166961319)

[^13^C NMR Spectrum of **4i** 19](#_Toc166961320)

[FT-IR Spectrum of **4j** 20](#_Toc166961321)

[^1^H NMR Spectrum of **4j** 20](#_Toc166961322)

[^13^C NMR Spectrum of **4j** 21](#_Toc166961323)

[MS Spectrum of **4j** 22](#_Toc166961324)

[FT-IR Spectrum of **4k** 22](#_Toc166961325)

[^1^H NMR Spectrum of **4k** 23](#_Toc166961326)

[^13^C NMR Spectrum of **4k** 23](#_Toc166961327)

[MS Spectrum of **4k** 24](#_Toc166961328)

[FT-IR Spectrum of **4l** 24](#_Toc166961329)

[^1^H NMR Spectrum of **4l** 25](#_Toc166961330)

[^13^C NMR Spectrum of **4l** 25](#_Toc166961331)

[MS Spectrum of **4l** 26](#_Toc166961332)

[FT-IR Spectrum of **4m** 26](#_Toc166961333)

[^1^H NMR Spectrum of **4m** 27](#_Toc166961334)

[^13^C NMR Spectrum of **4m** 27](#_Toc166961335)

[MS Spectrum of **4m** 28](#_Toc166961336)

[FT-IR Spectrum of **4n** 28](#_Toc166961337)

[^1^H NMR Spectrum of **4n** 29](#_Toc166961338)

[^13^C NMR Spectrum of **4n** 29](#_Toc166961339)

[MS Spectrum of **4n** 30](#_Toc166961340)


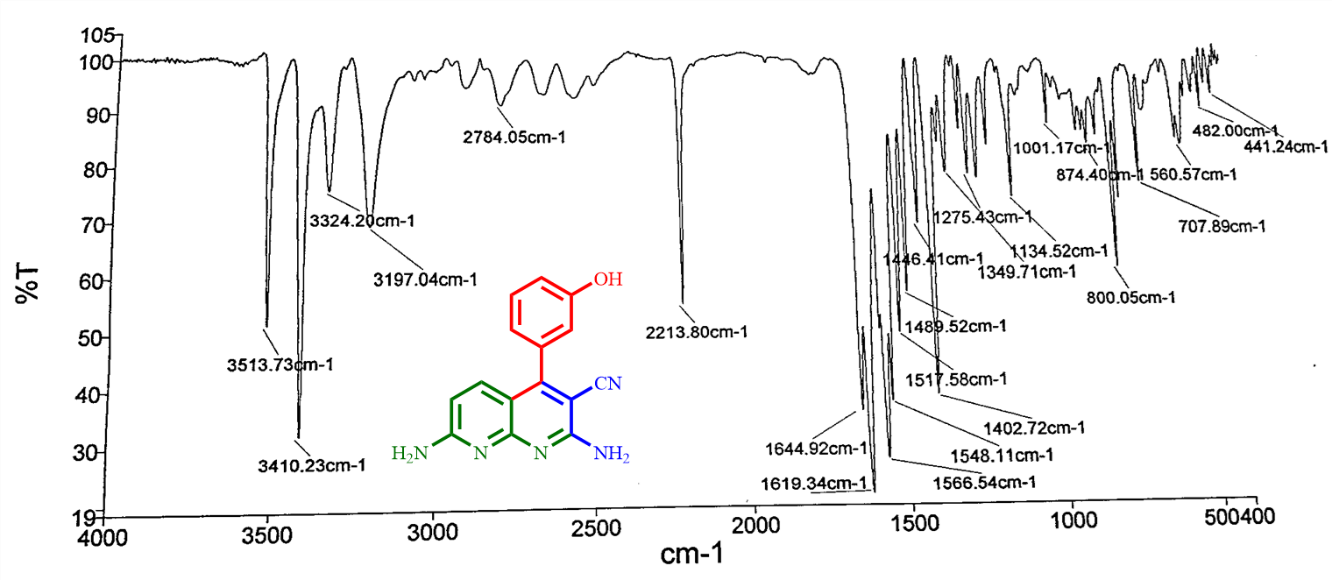


# FT-IR Spectrum of **4a**


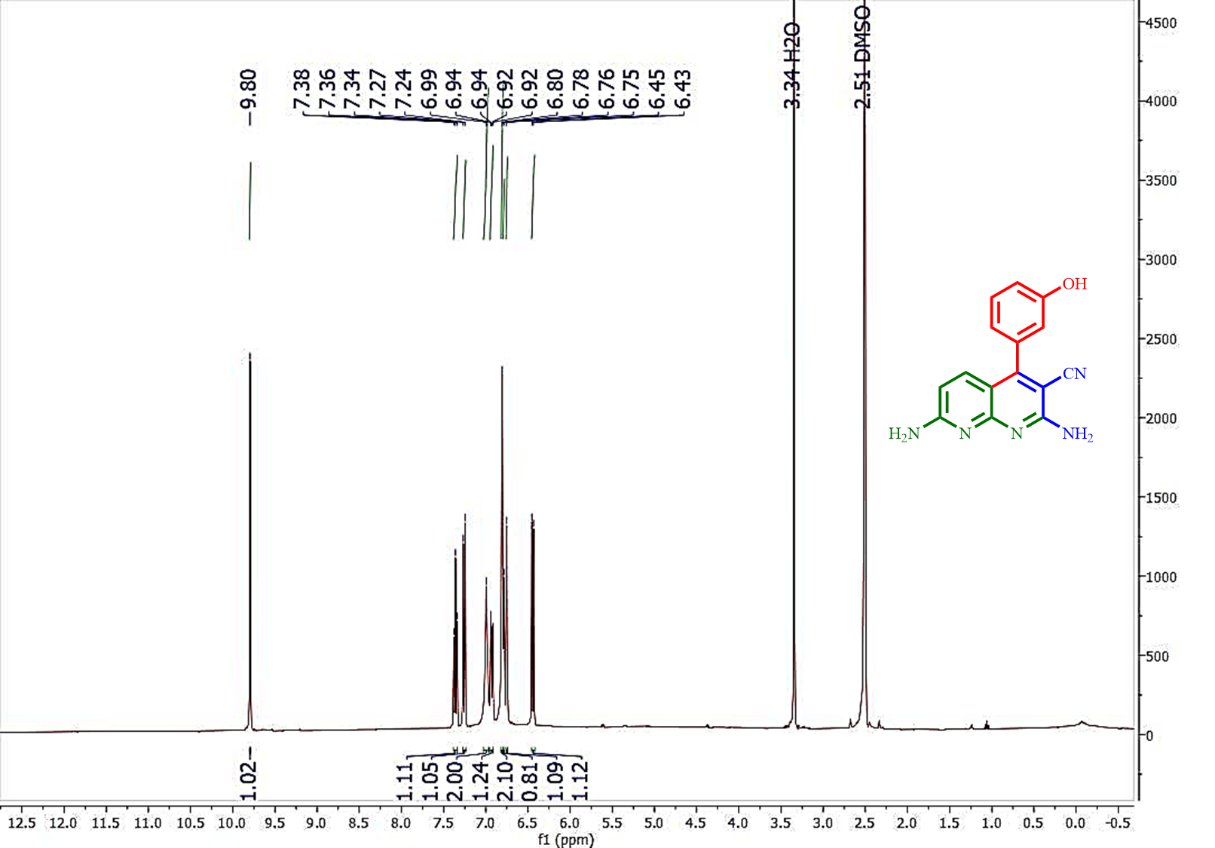


# ^1^H NMR Spectrum of **4a**


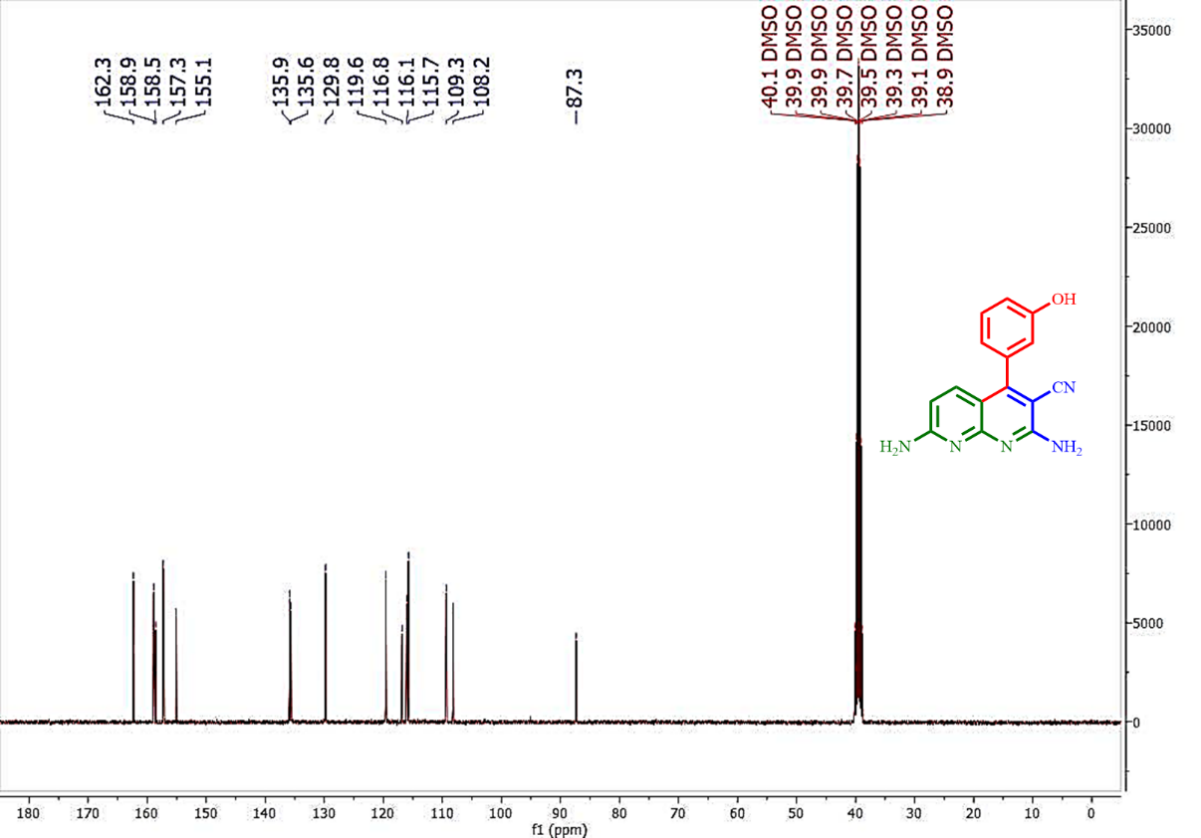


# ^13^C NMR Spectrum of **4a**


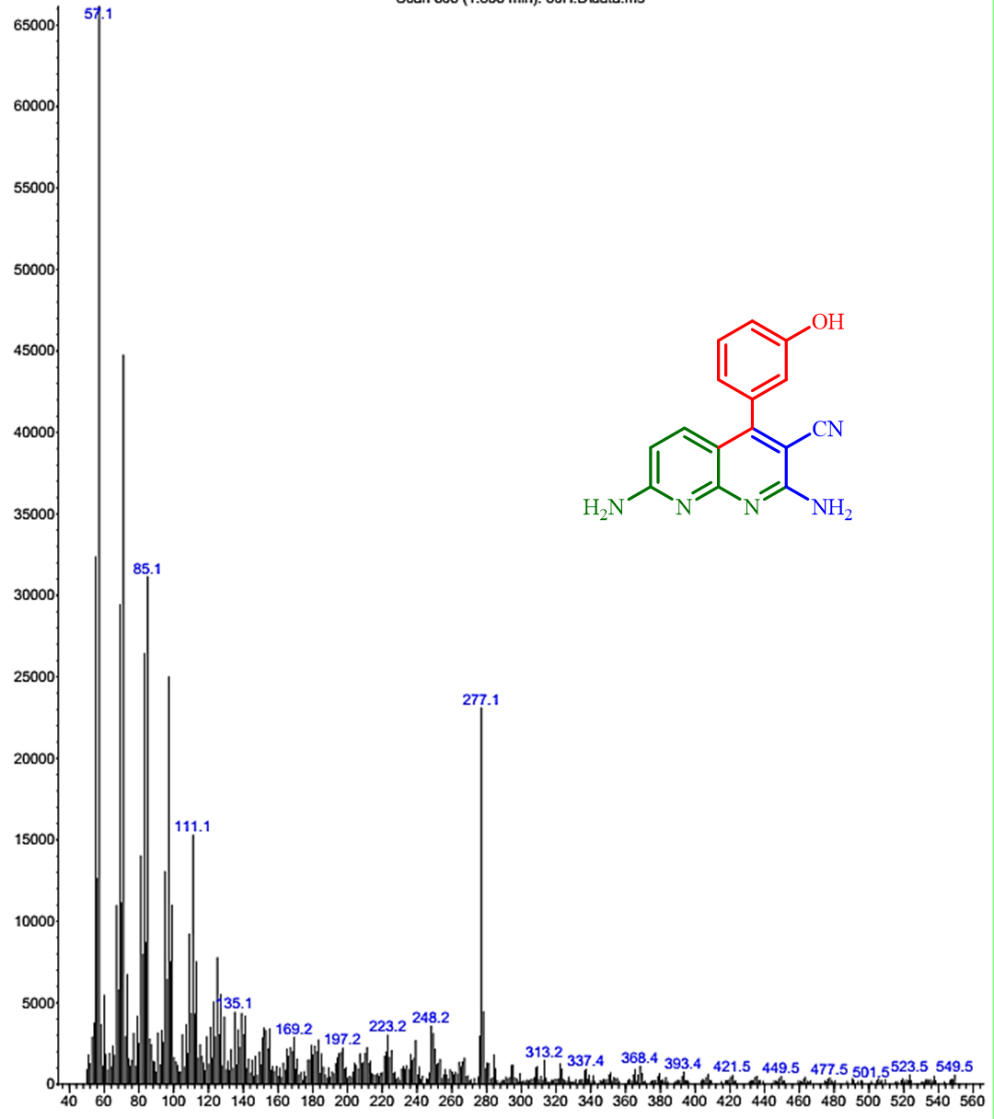


# MS Spectrum of **4a**


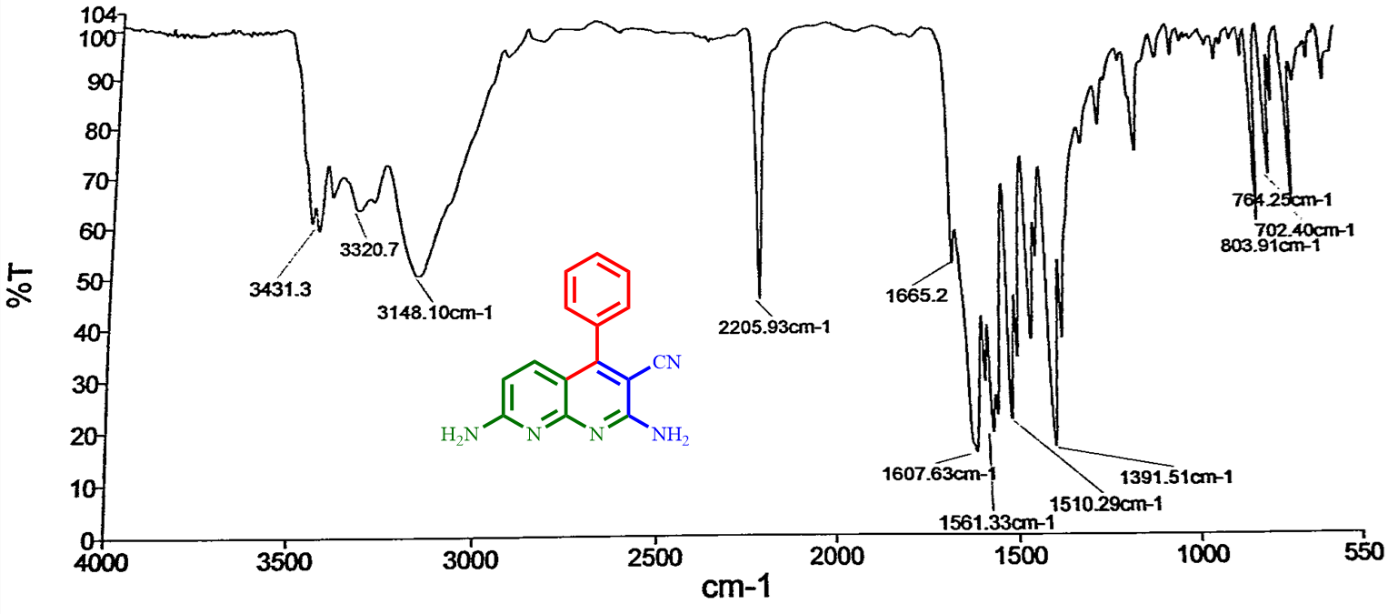


# FT-IR Spectrum of **4b**


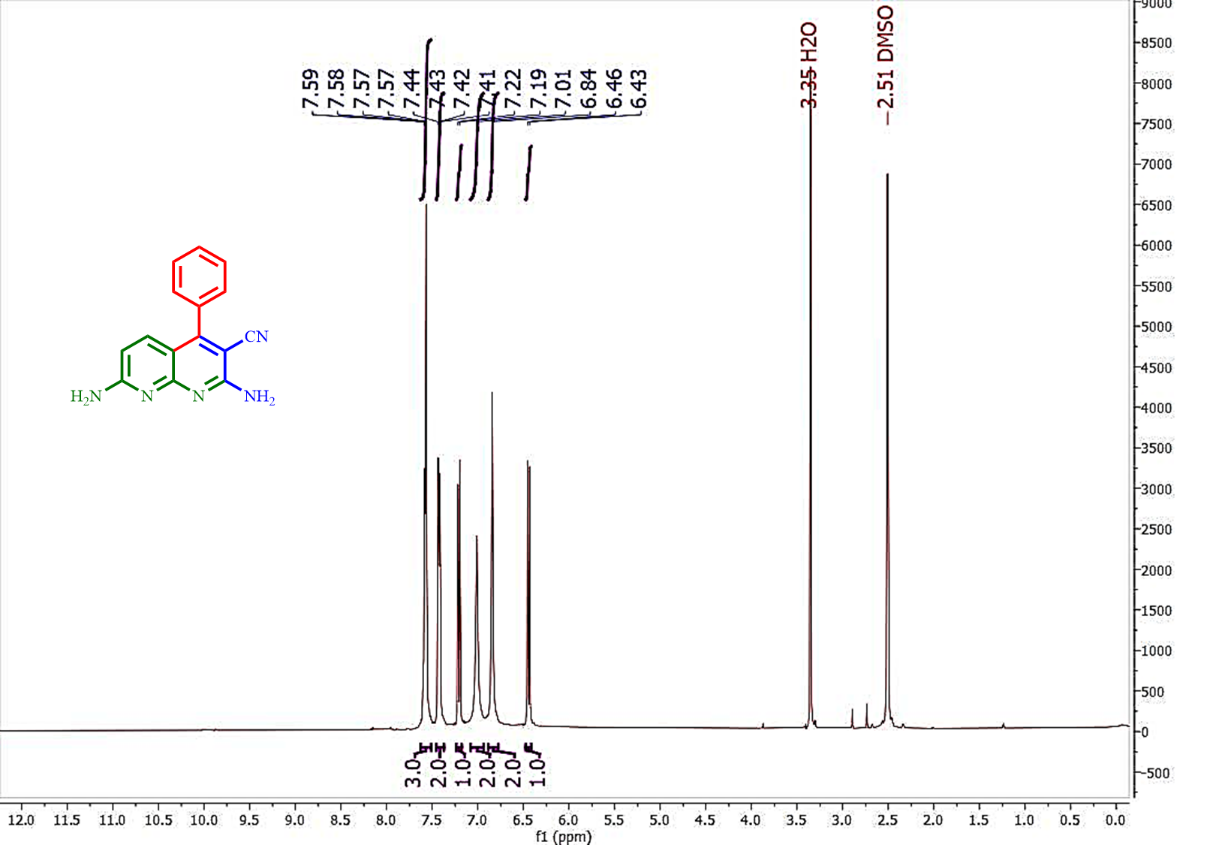


# ^1^H NMR Spectrum of **4b**


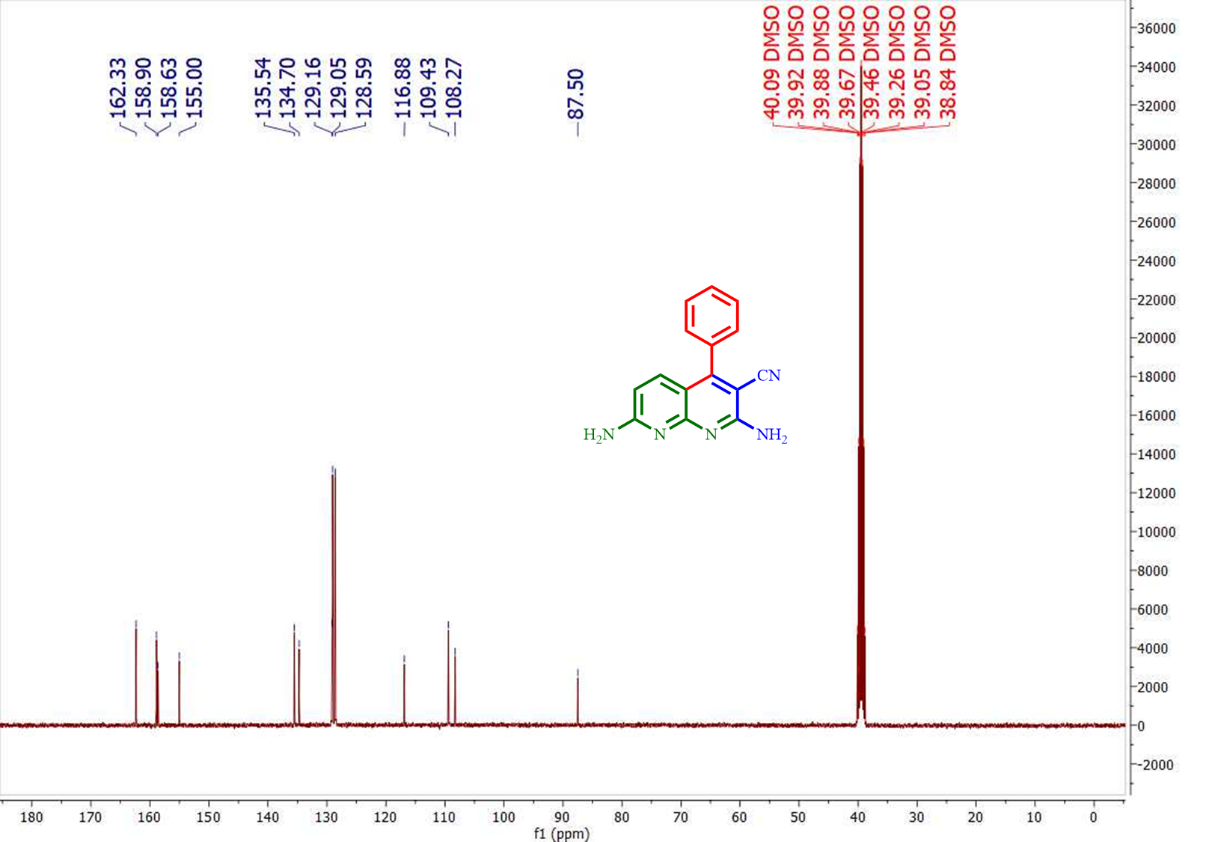


# ^13^C NMR Spectrum of **4b**


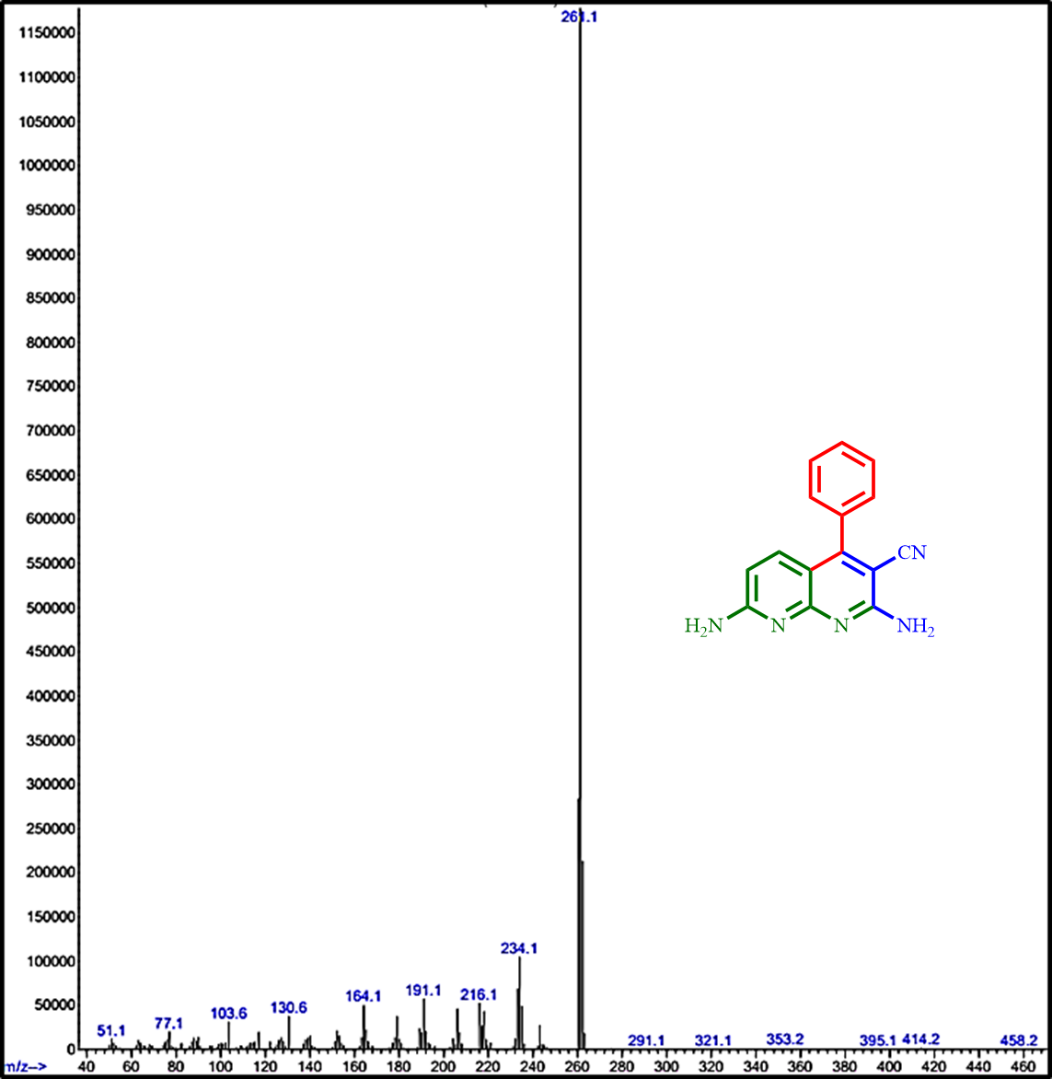


# MS Spectrum of **4b**


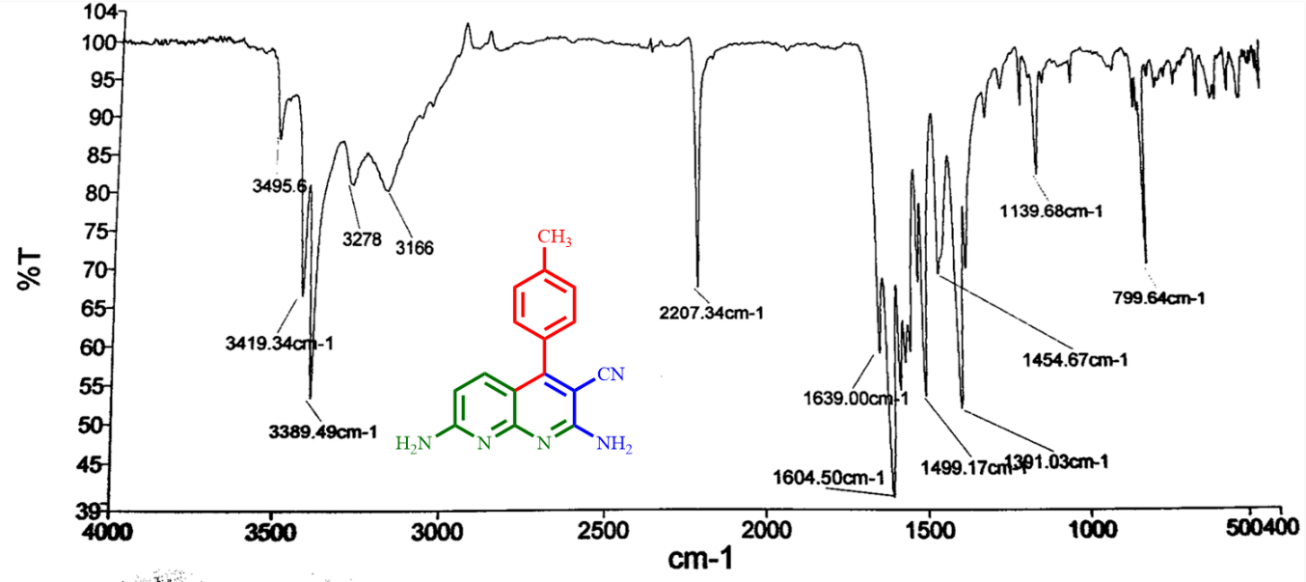


# FT-IR Spectrum of **4c**


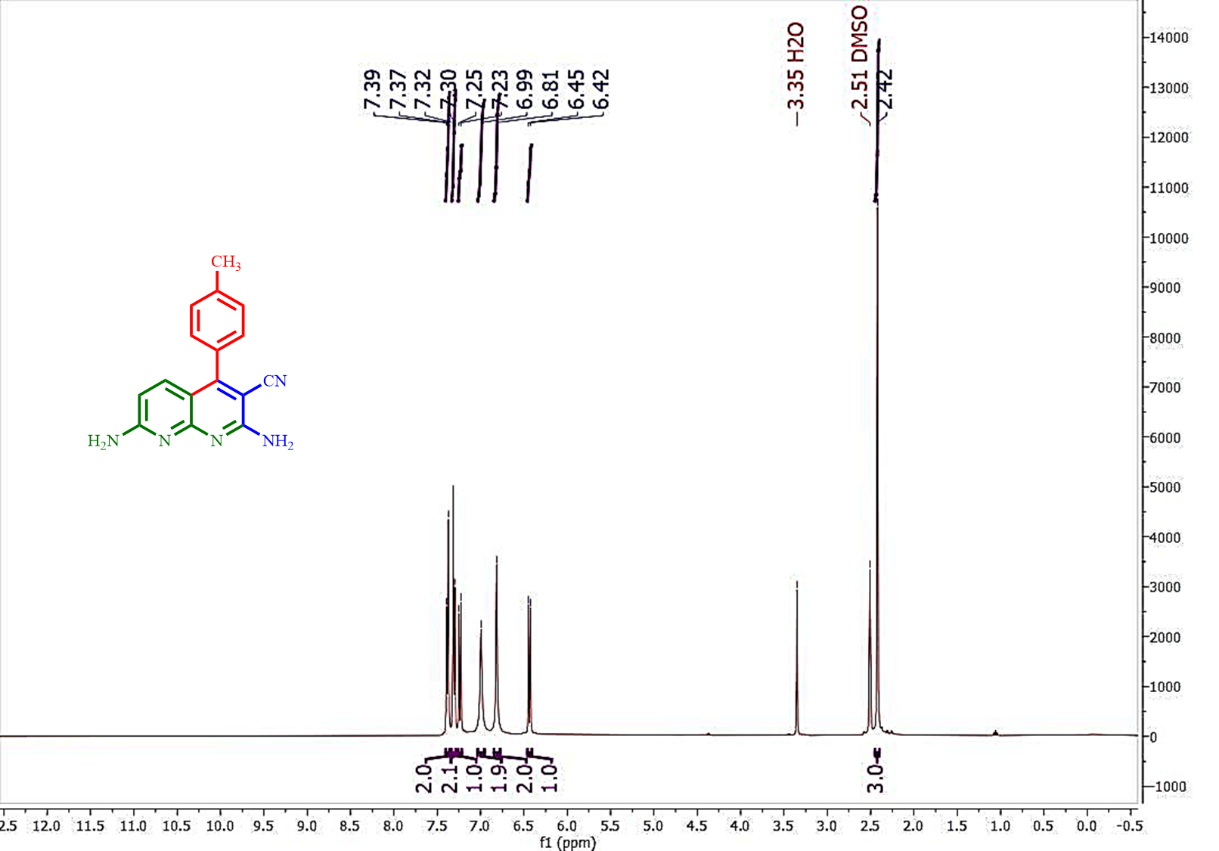


# ^1^H NMR Spectrum of **4c**


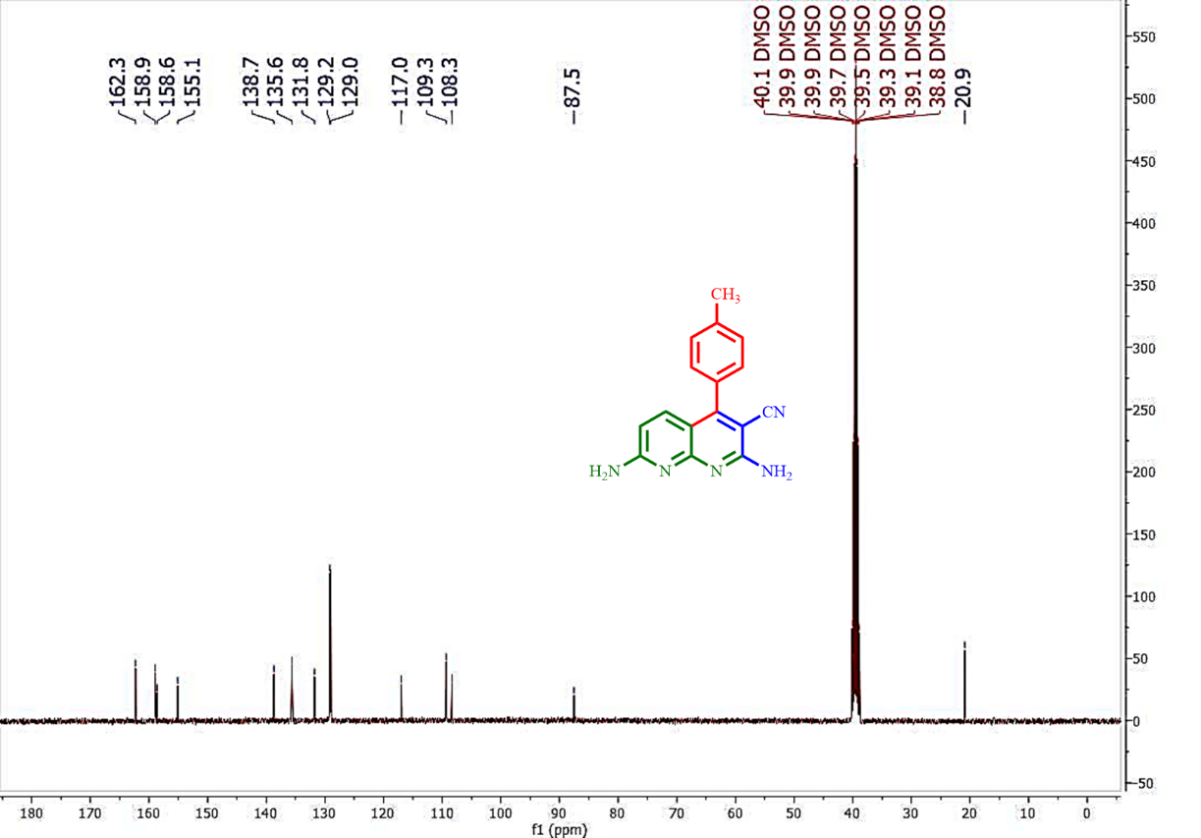


# ^13^C NMR Spectrum of **4c**


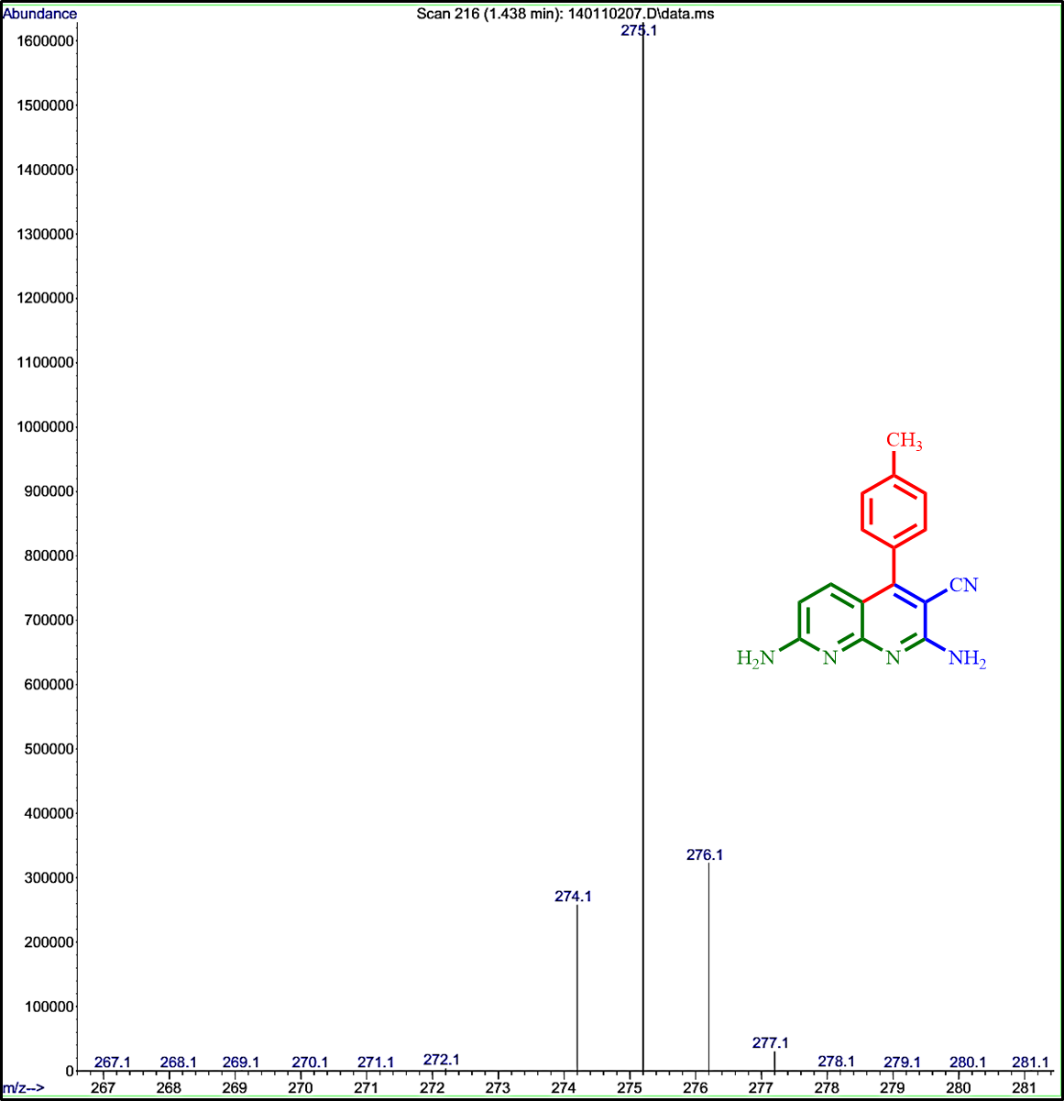


# MS Spectrum of **4c**


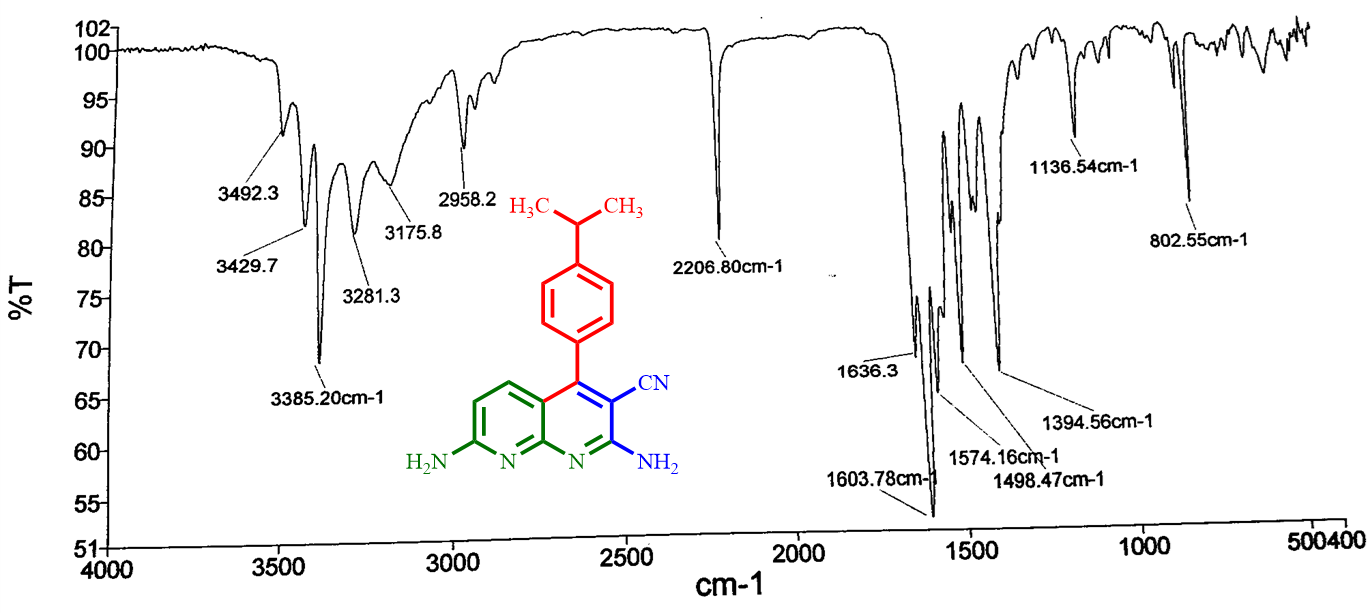


# FT-IR Spectrum of **4d**


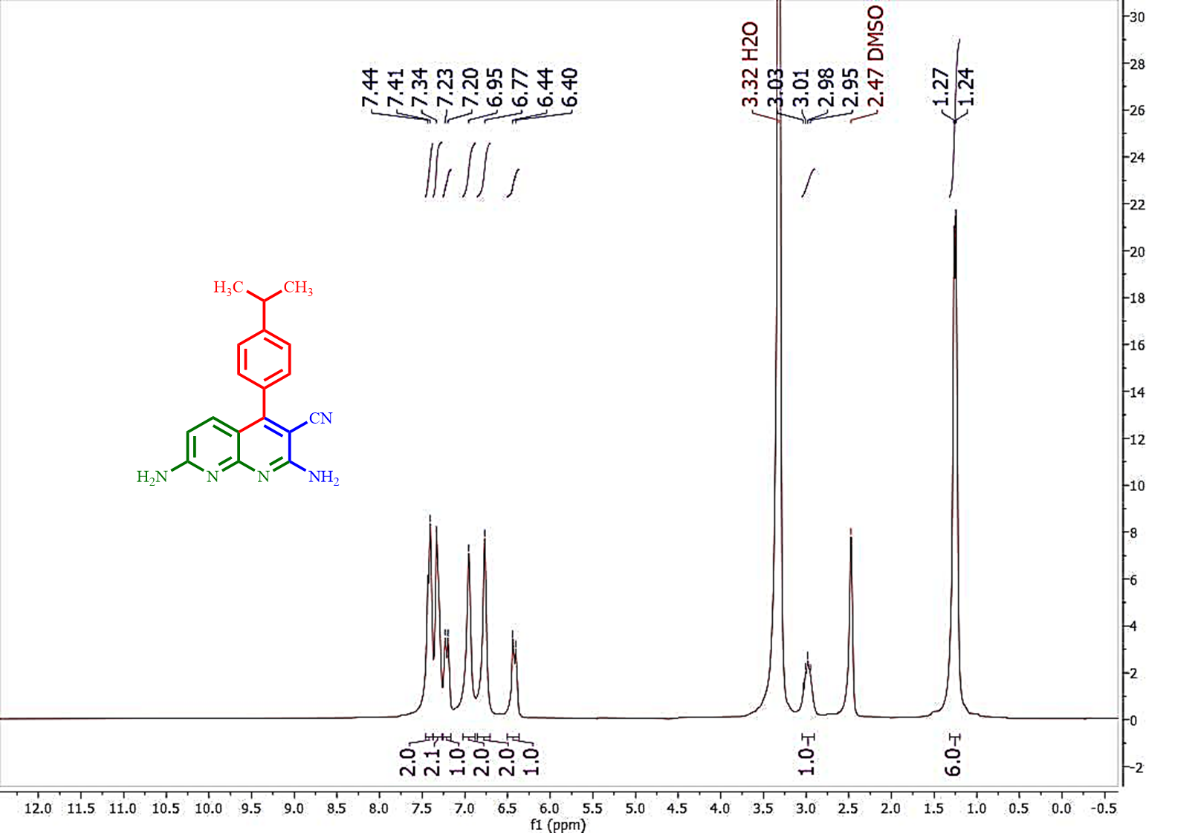


# ^1^H NMR Spectrum of **4d**


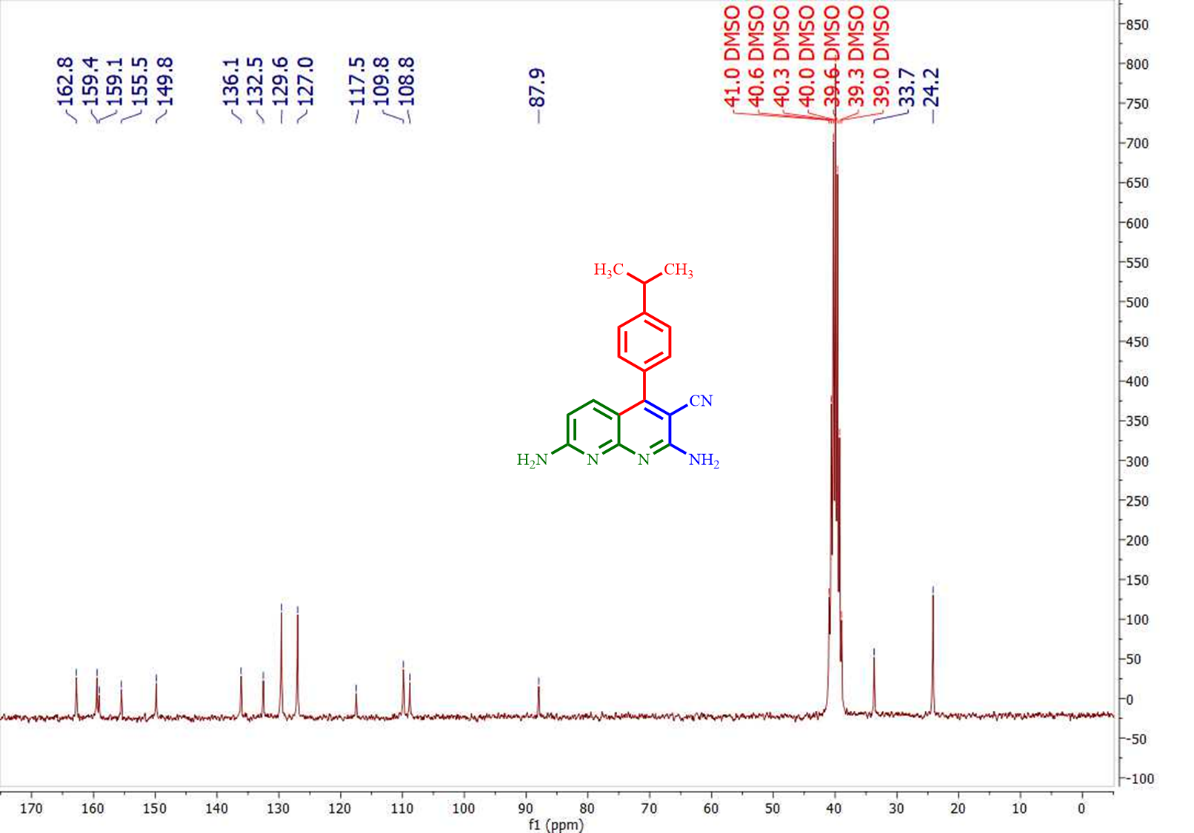


# ^13^C NMR Spectrum of **4d**

**
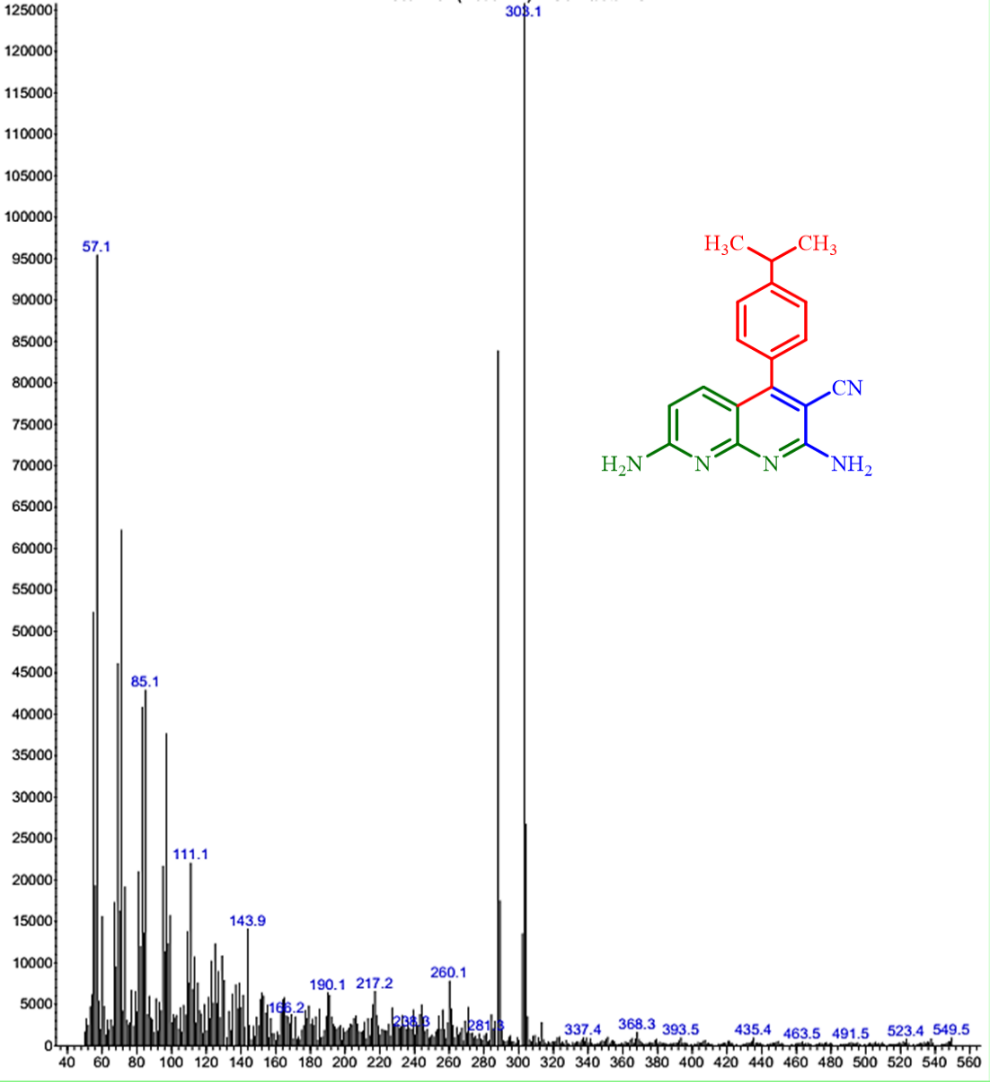
**

# MS Spectrum of **4d**


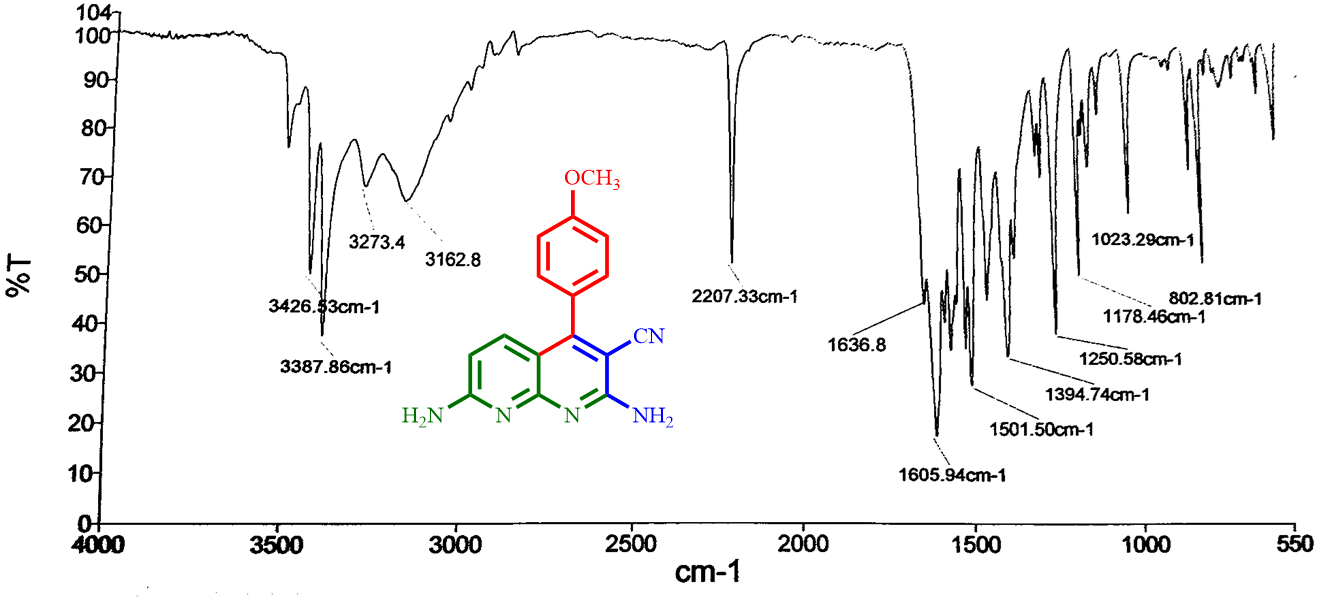


# FT-IR Spectrum of **4e**


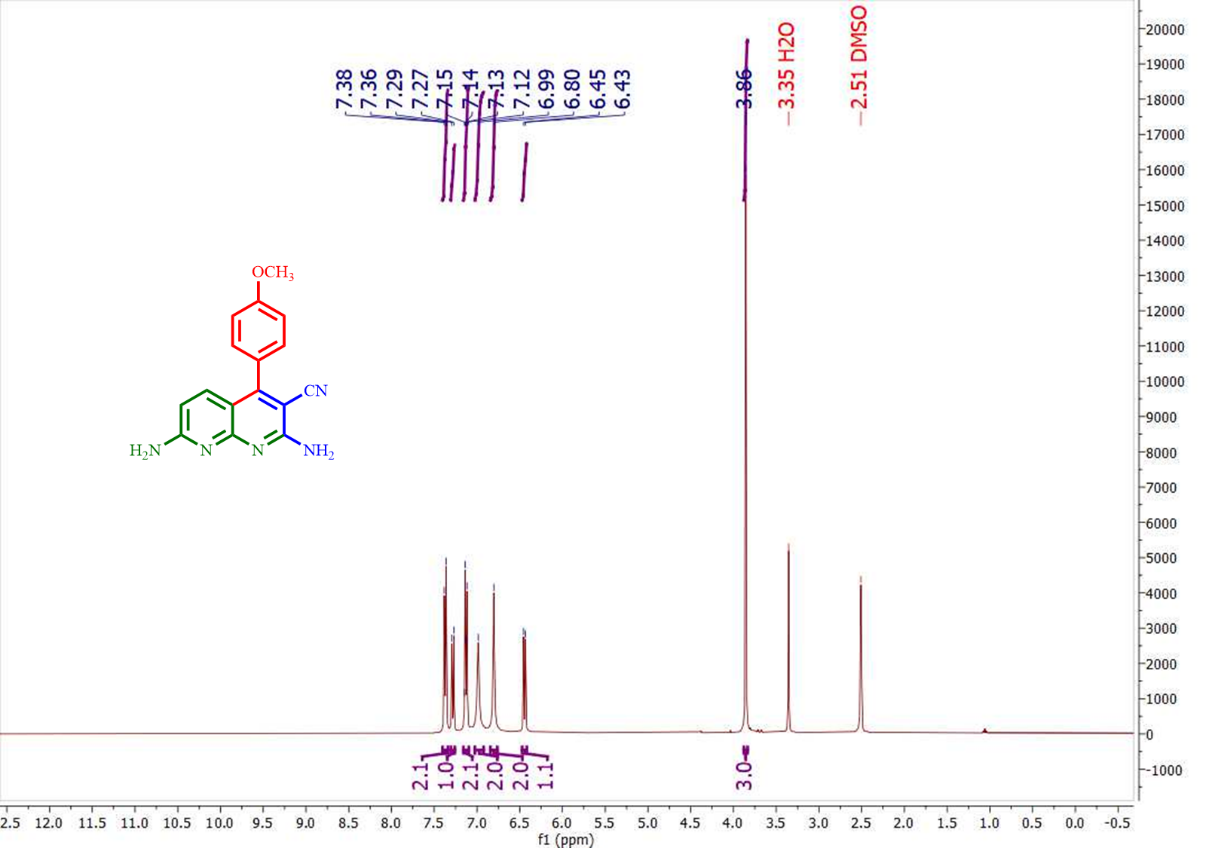


# ^1^H NMR Spectrum of **4e**


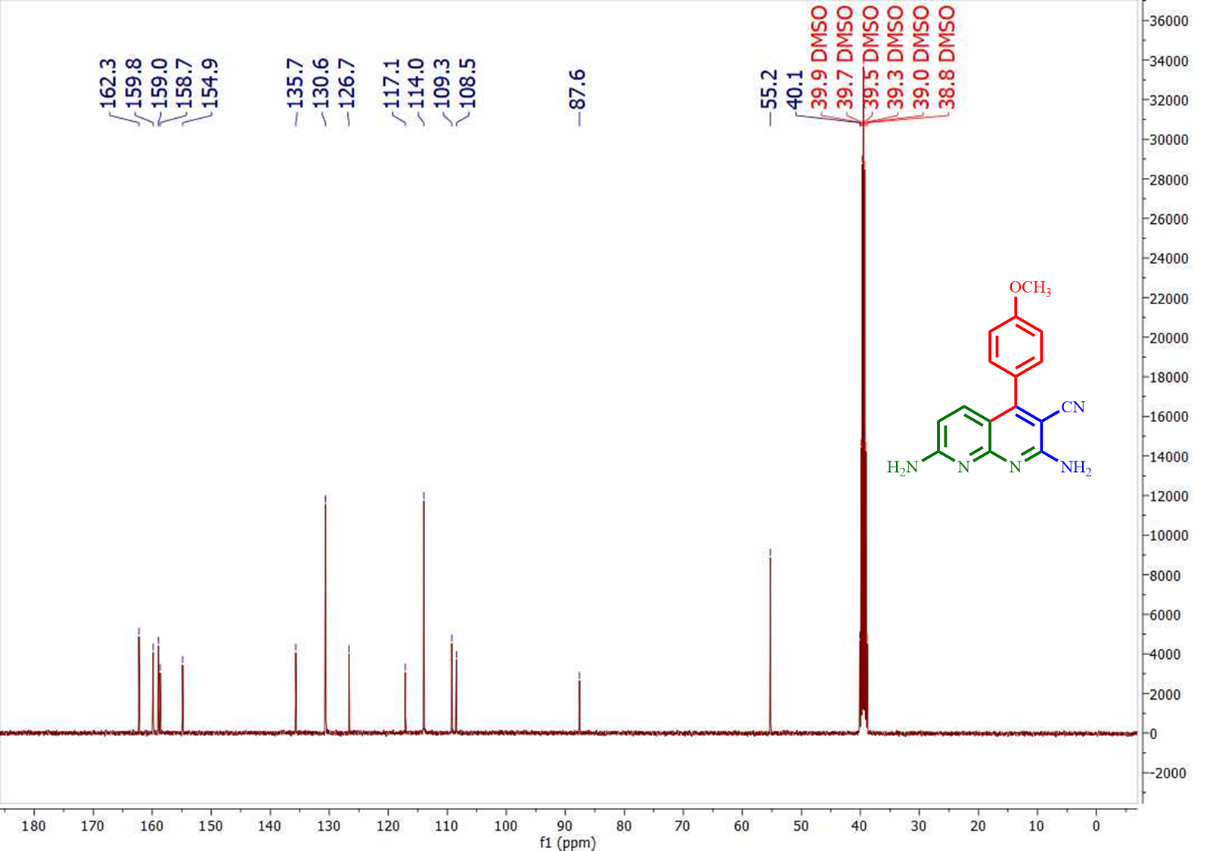


# ^13^C NMR Spectrum of **4e**


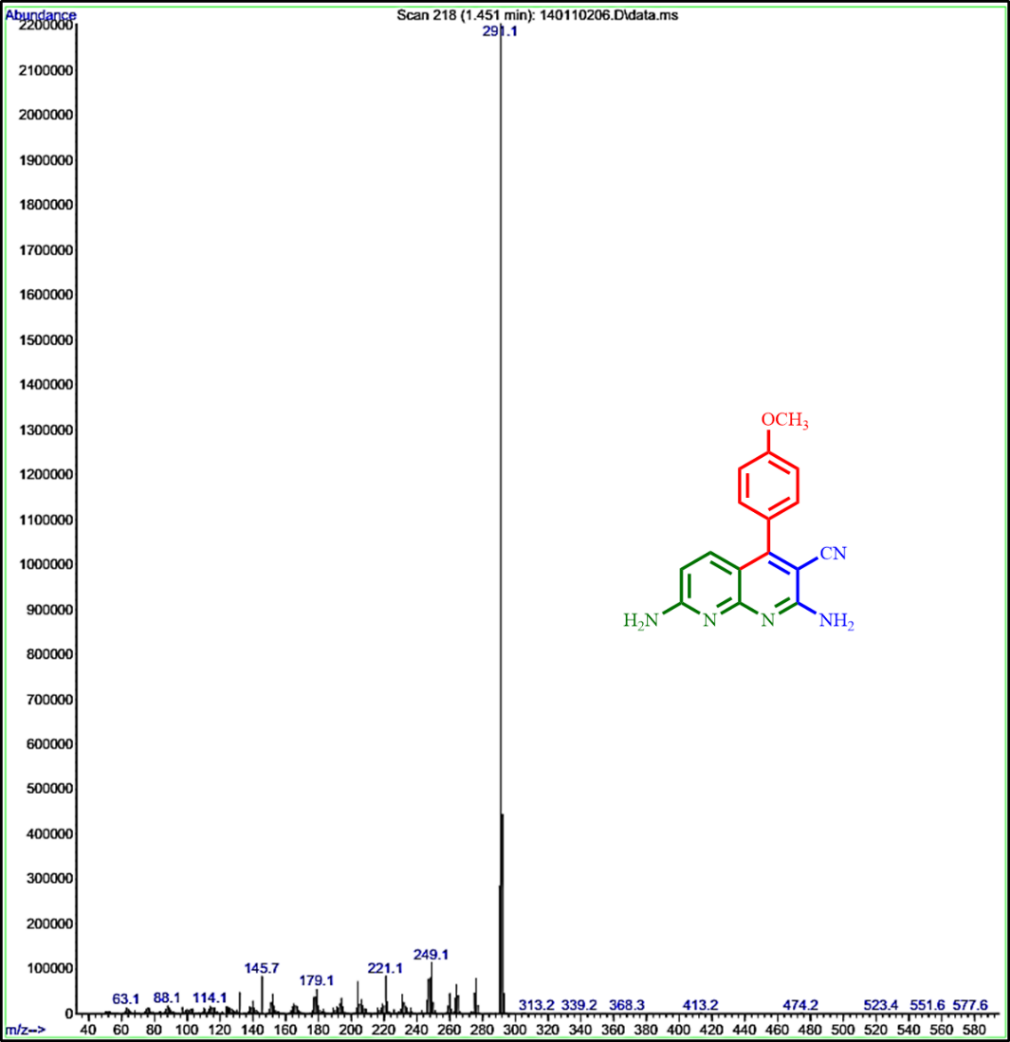


# MS Spectrum of **4e**


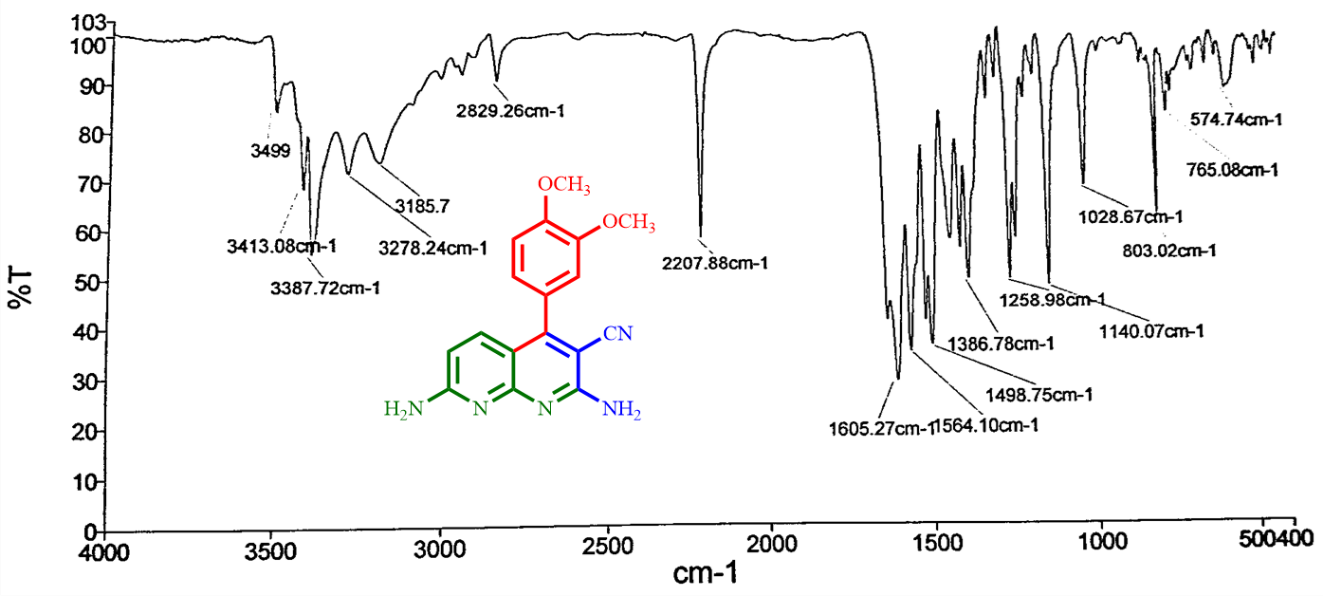


# FT-IR Spectrum of **4f**


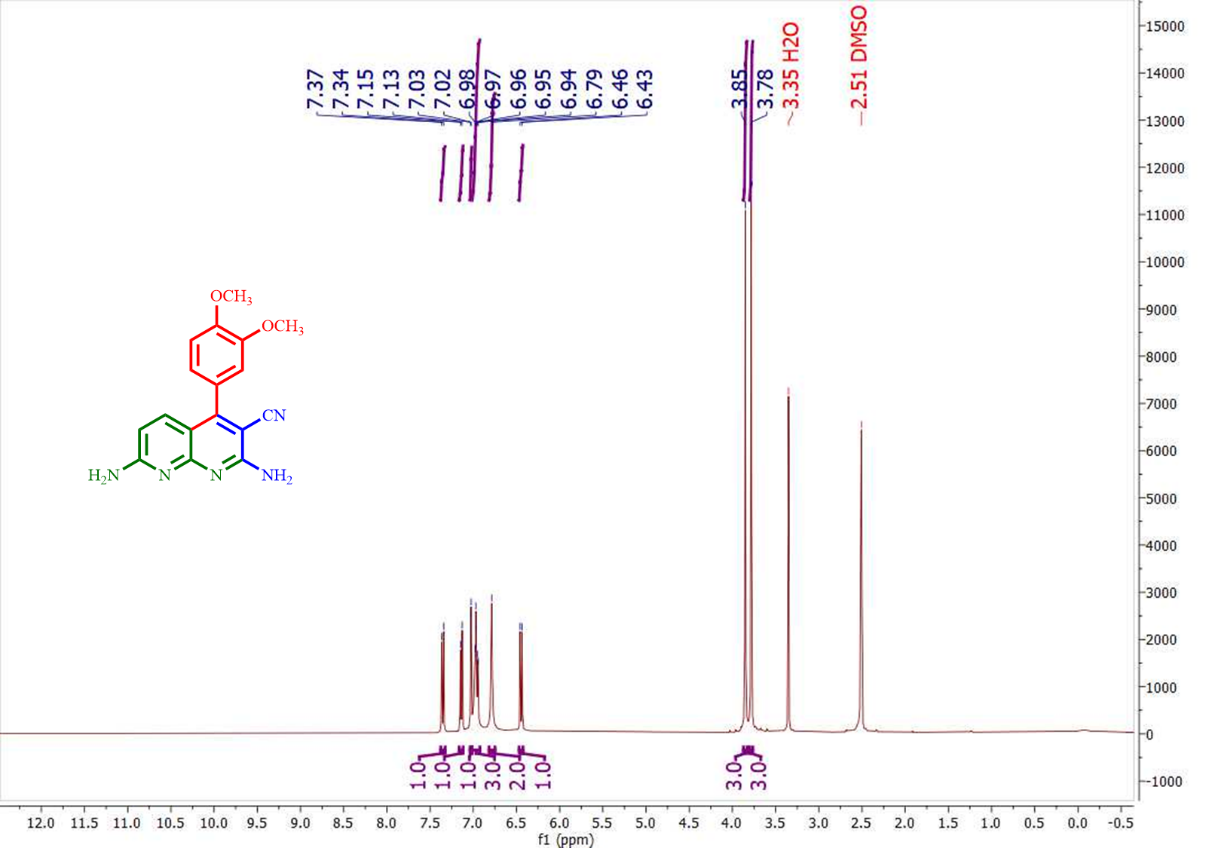


# ^1^H NMR Spectrum of **4f**


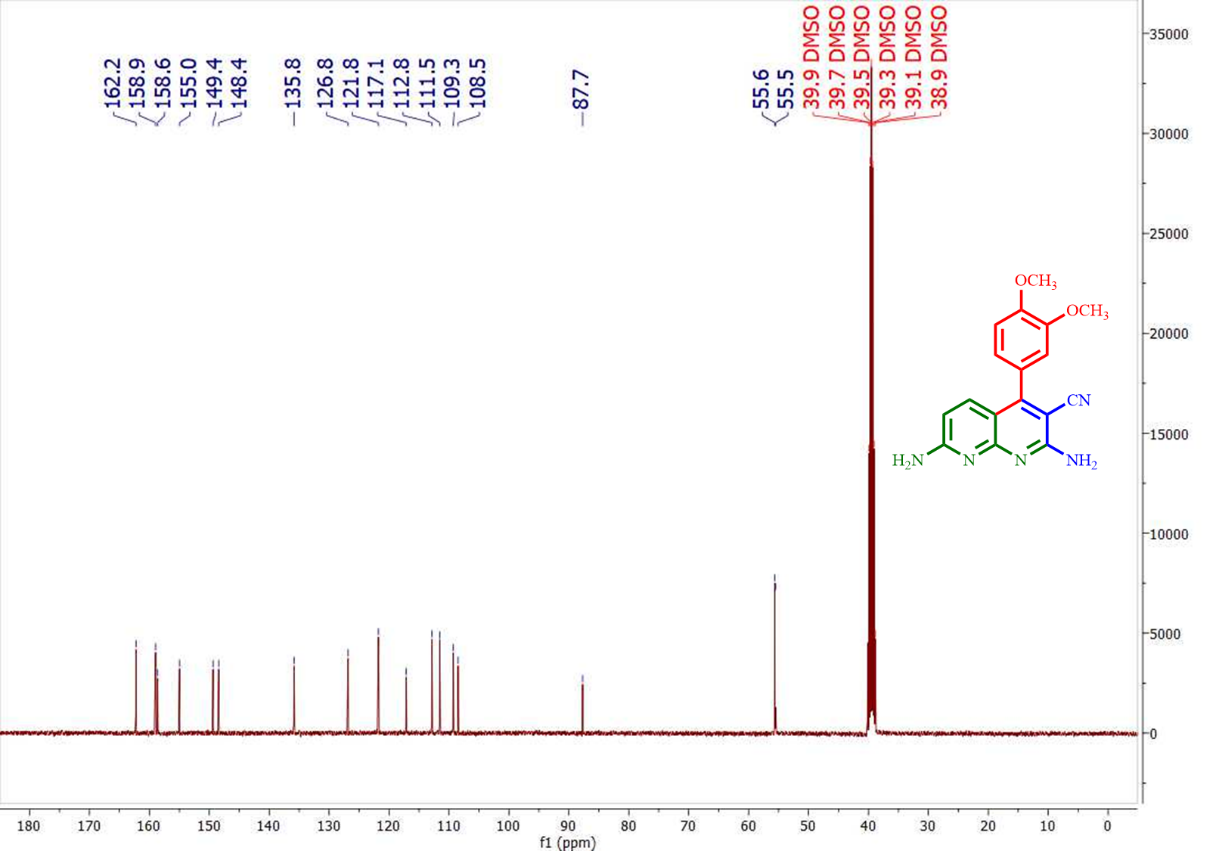


# ^13^C NMR Spectrum of **4f**


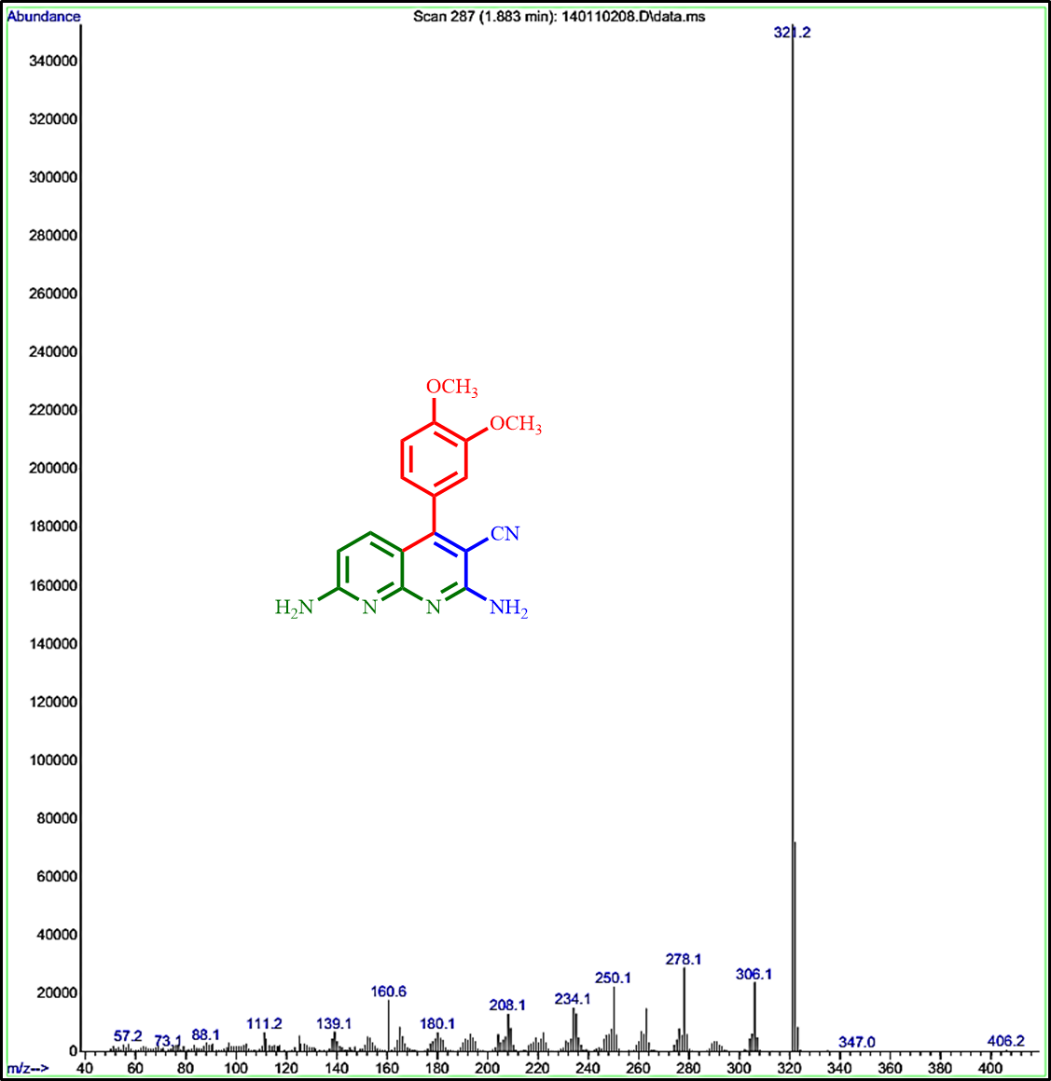


# MS Spectrum of **4f**


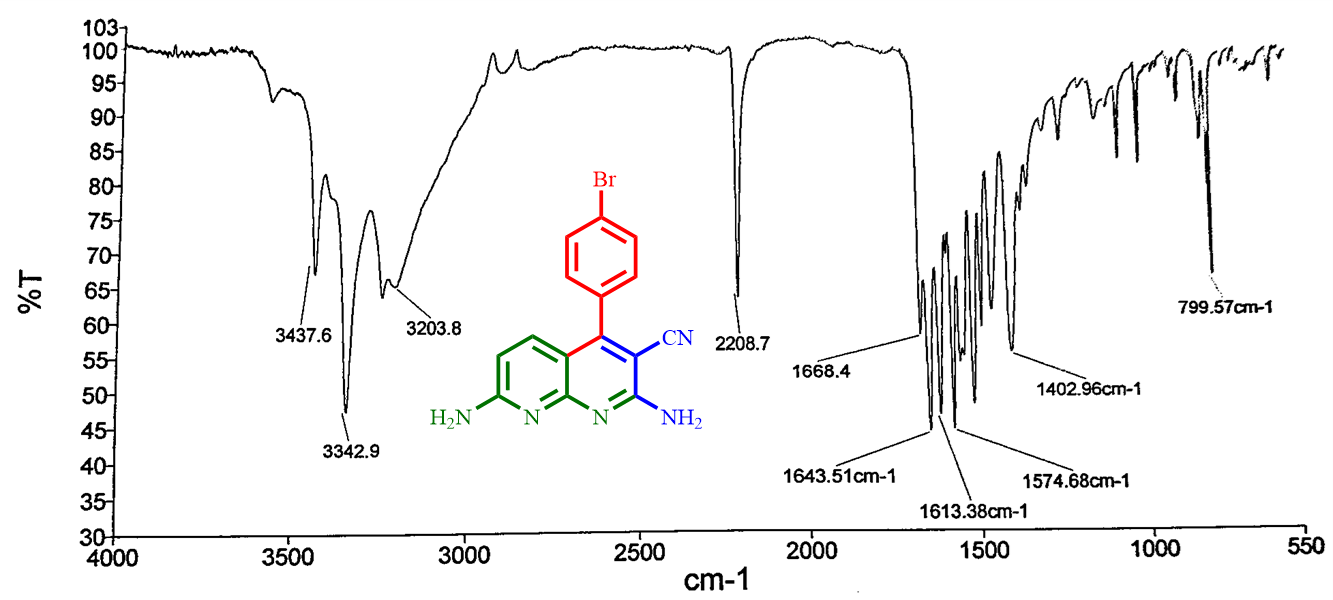


# FT-IR Spectrum of **4g**


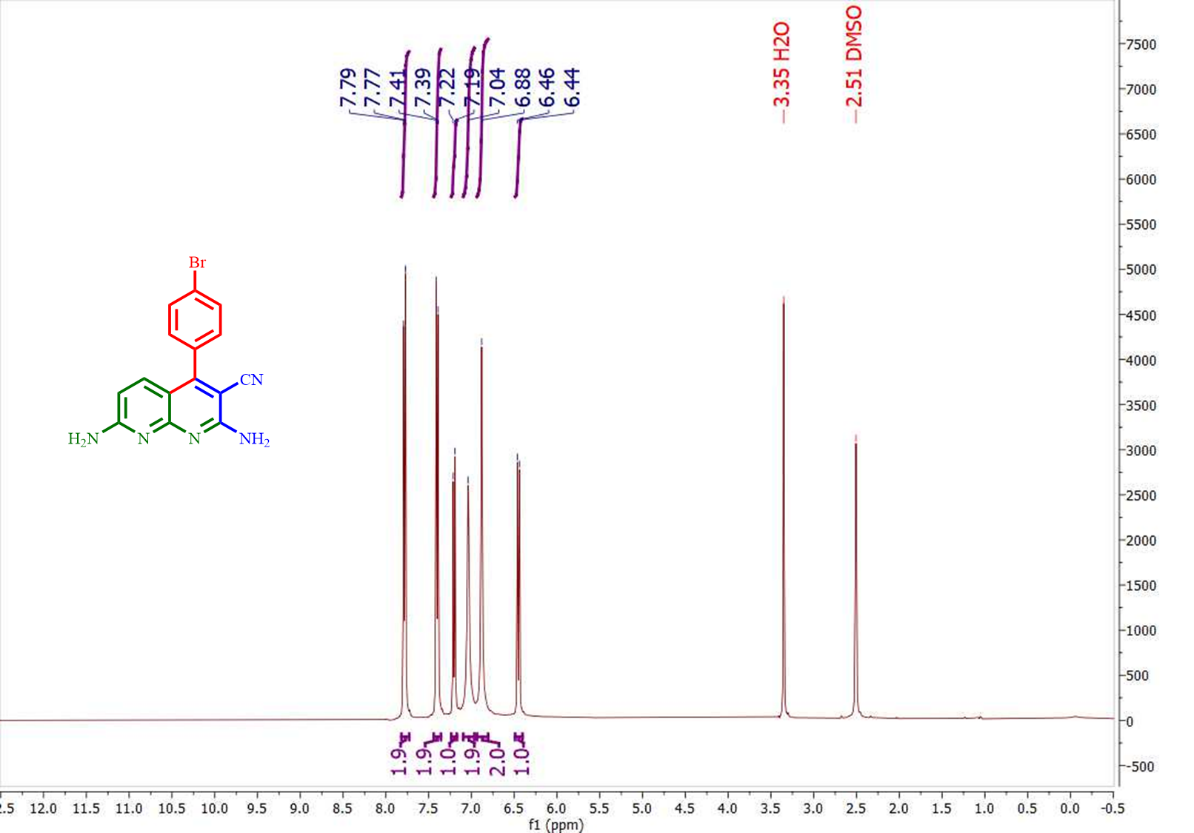


# ^1^H NMR Spectrum of **4g**


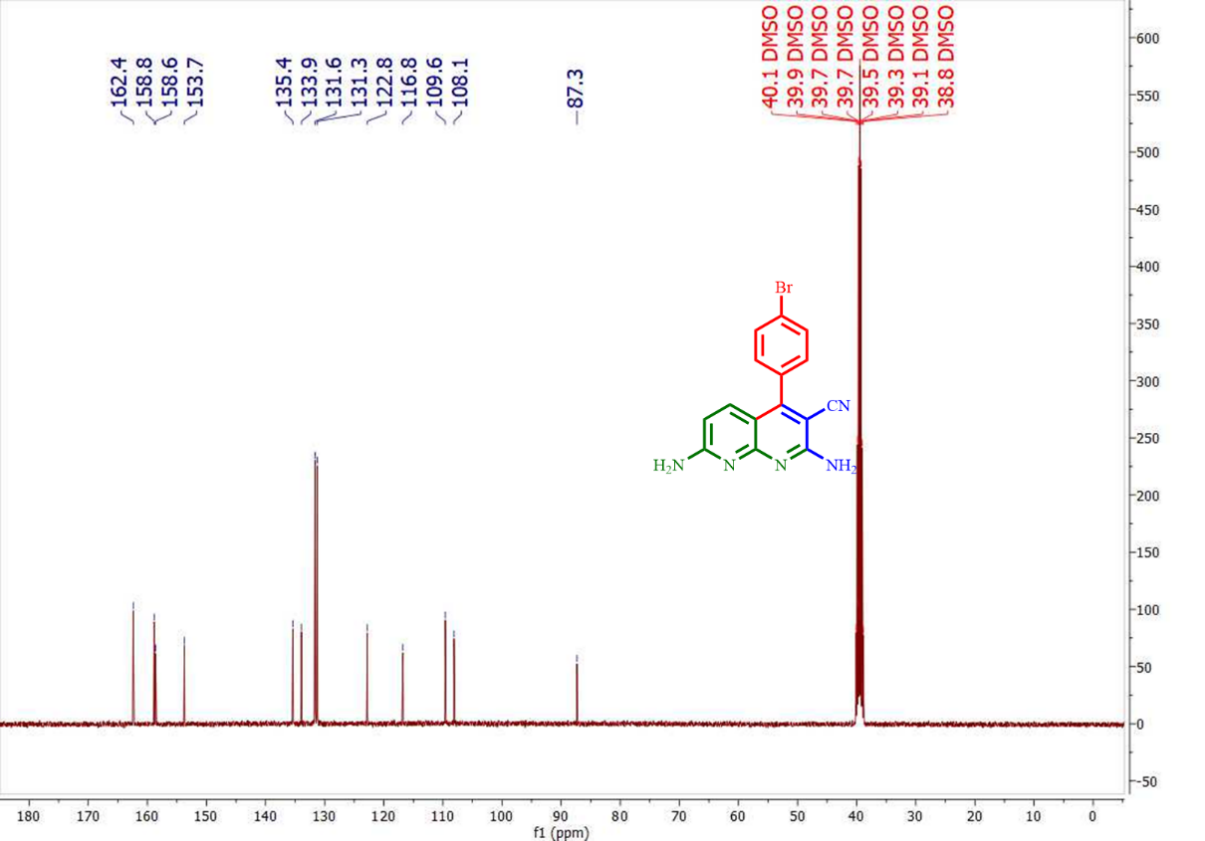


# ^13^C NMR Spectrum of **4g**

**
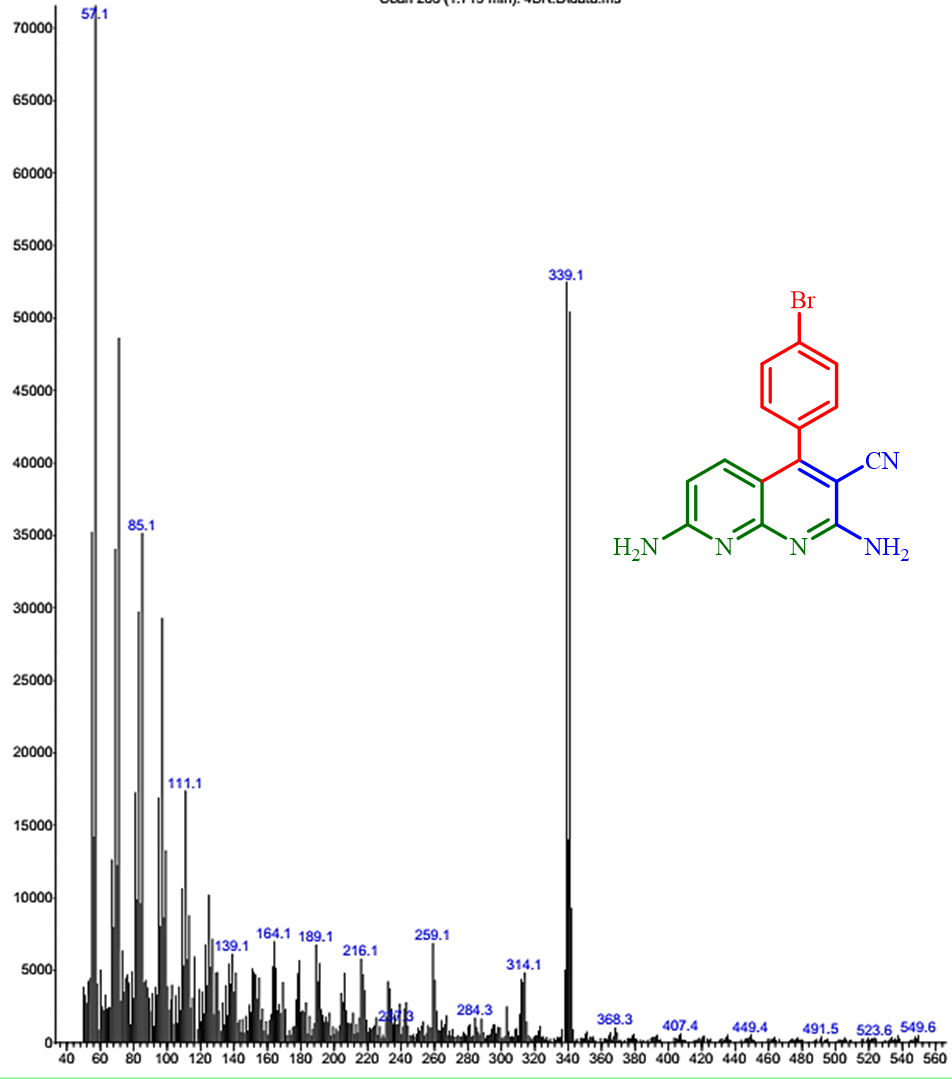
**

# MS Spectrum of **4g**


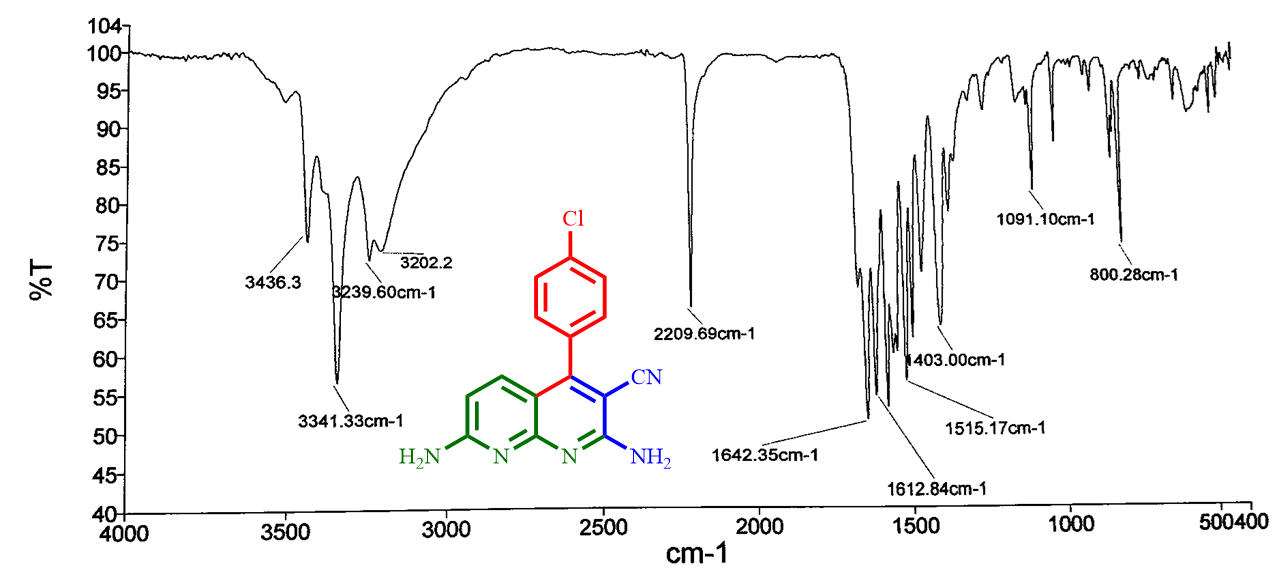


# FT-IR Spectrum of **4h**


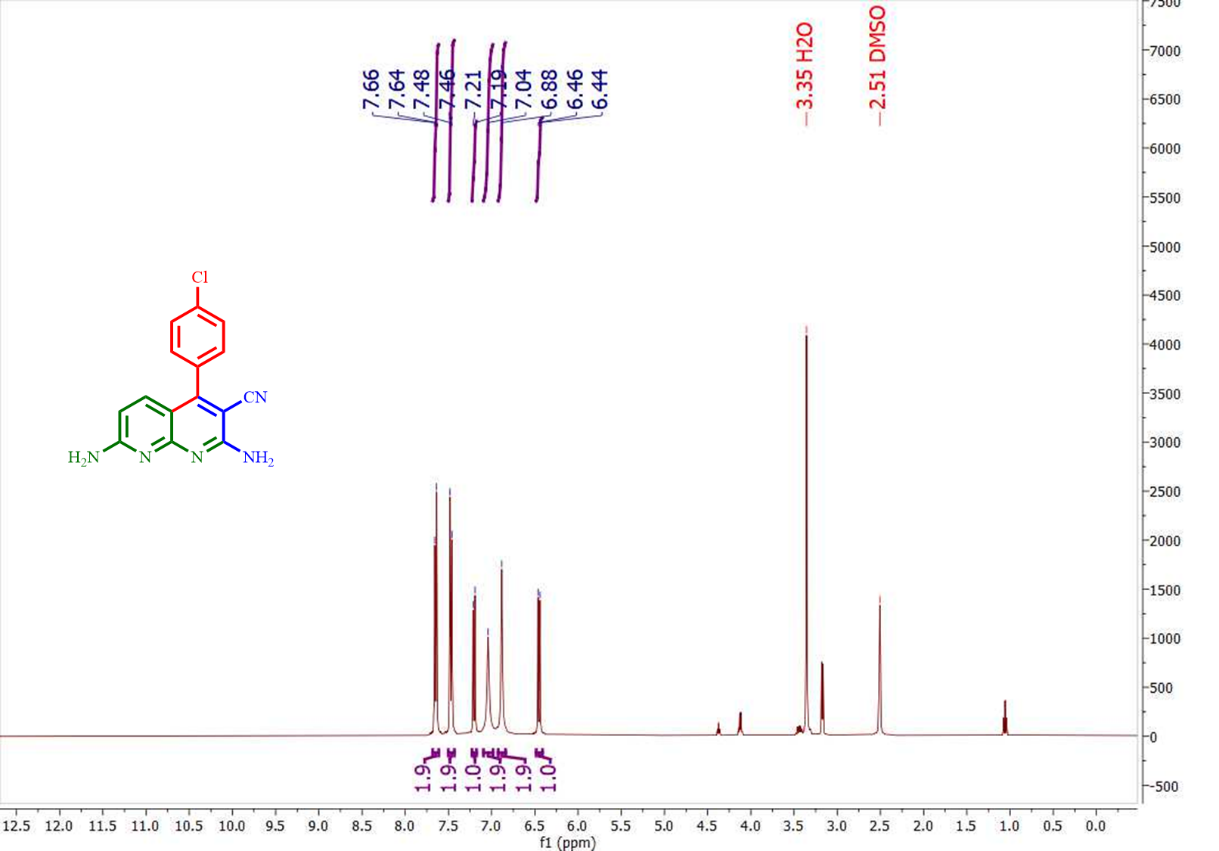


# ^1^H NMR Spectrum of **4h**


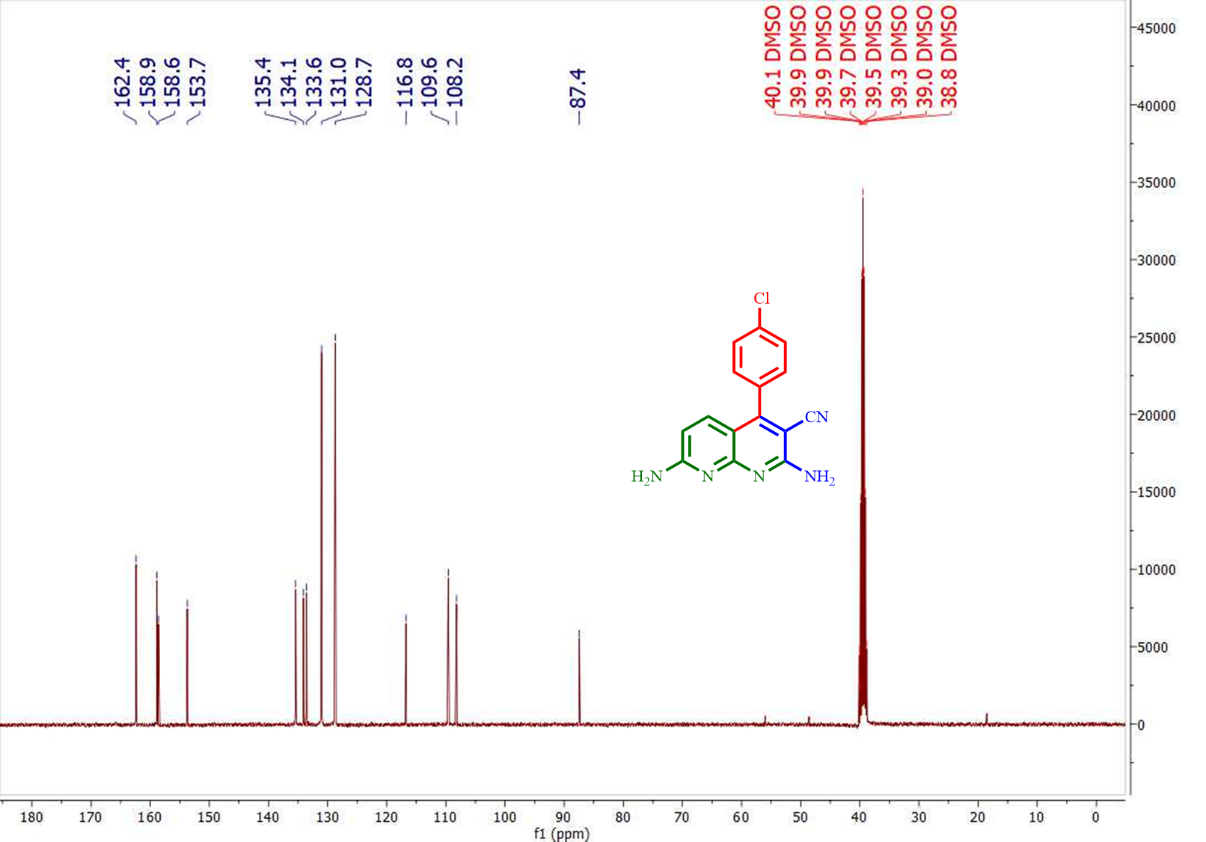


# ^13^C NMR Spectrum of **4h**


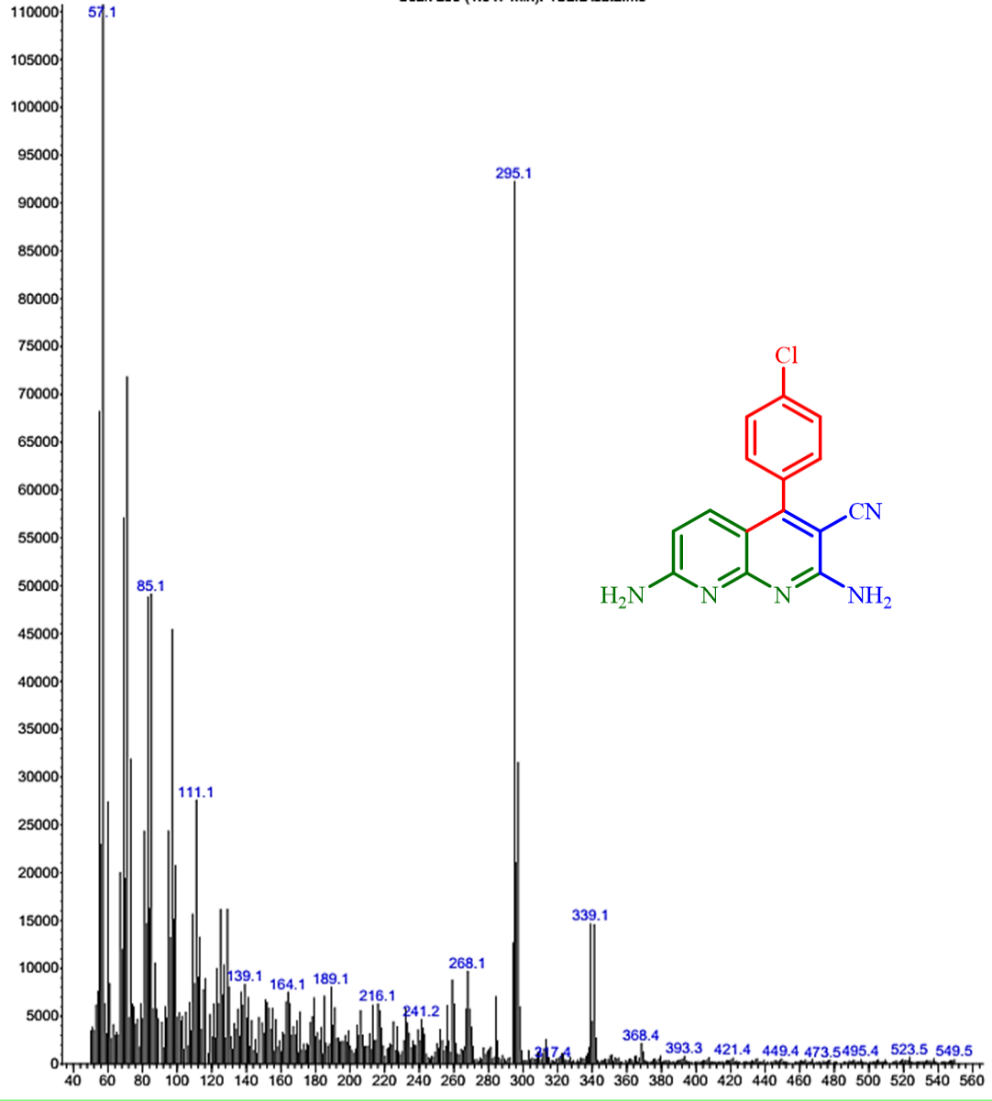


# MS Spectrum of **4h**


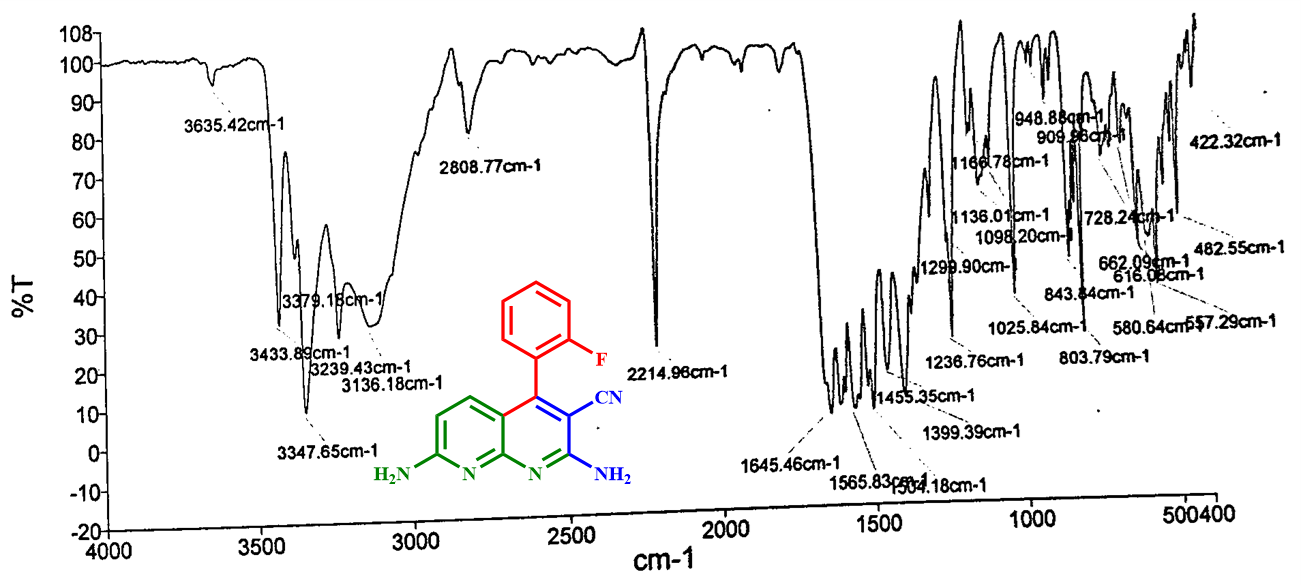


# FT-IR Spectrum of **4i**


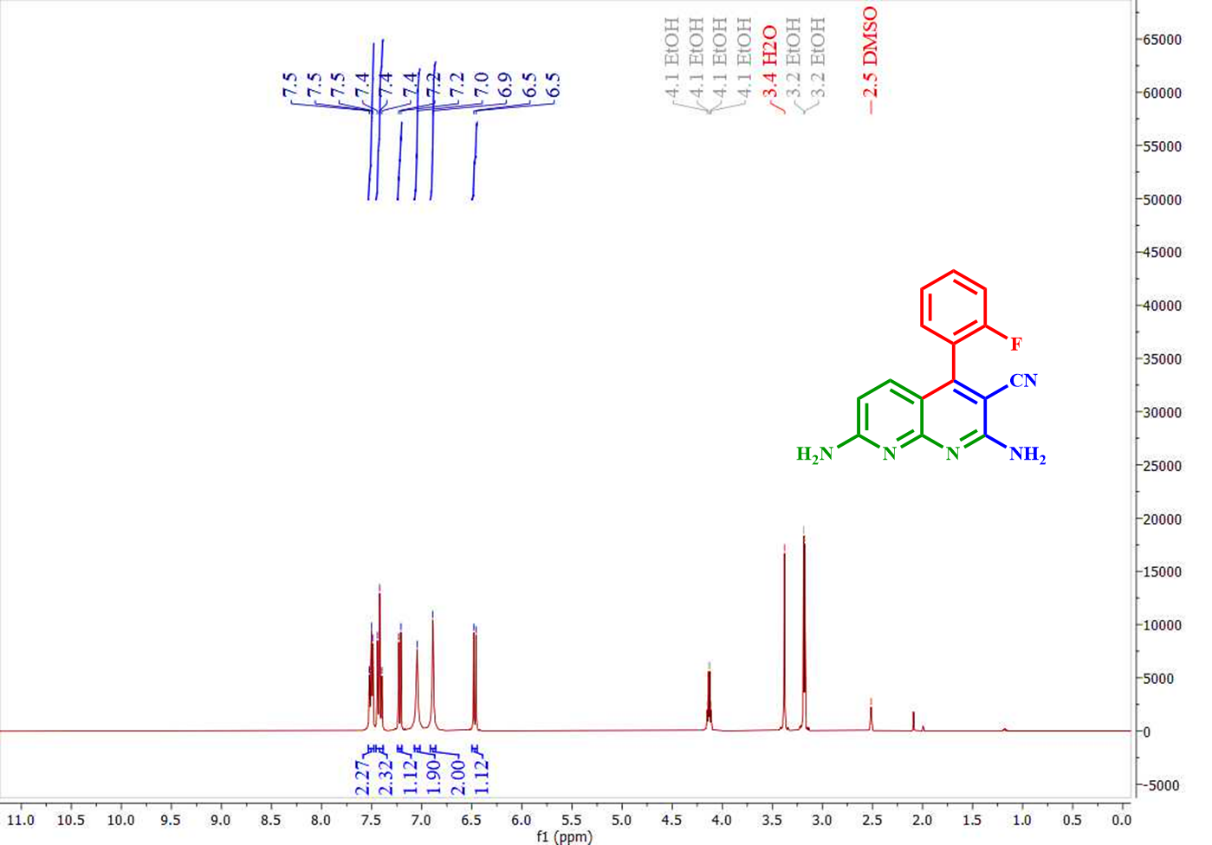


# ^1^H NMR Spectrum of **4i**


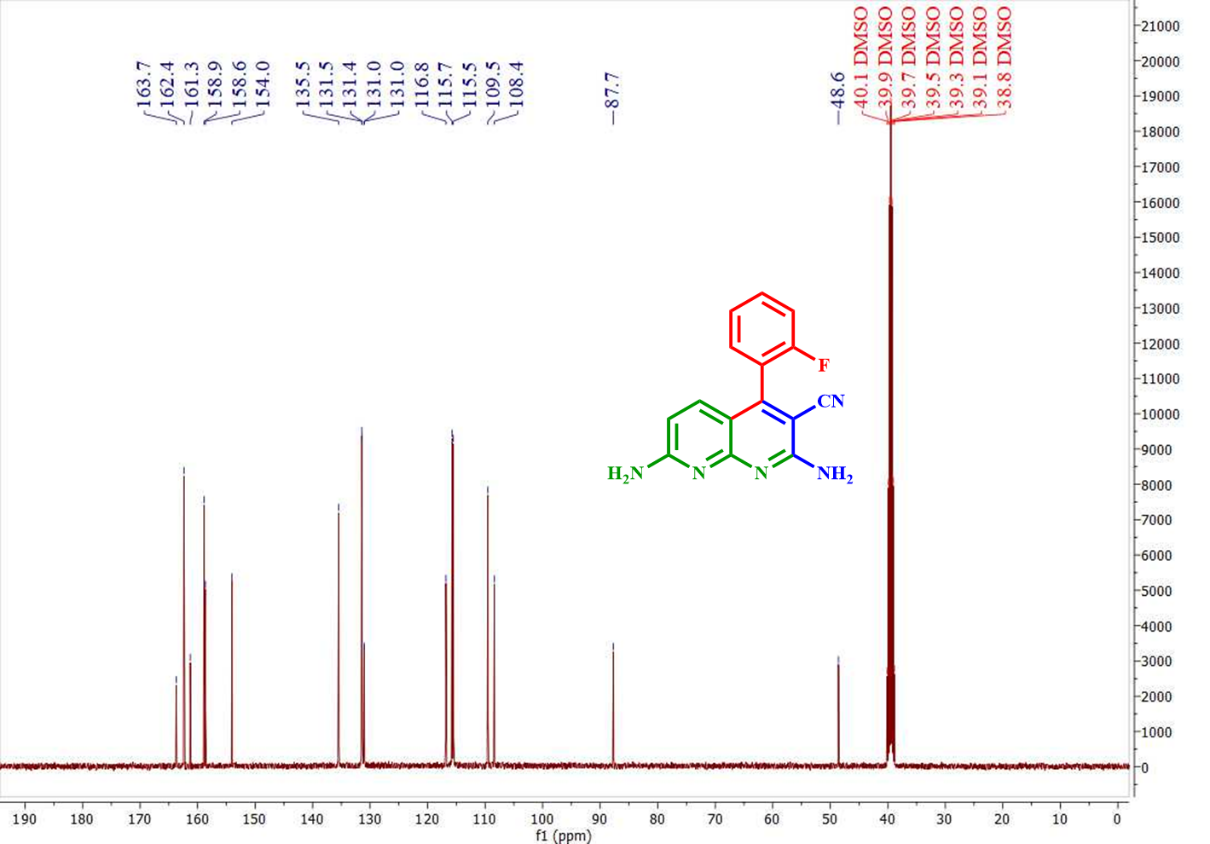


# ^13^C NMR Spectrum of **4i**


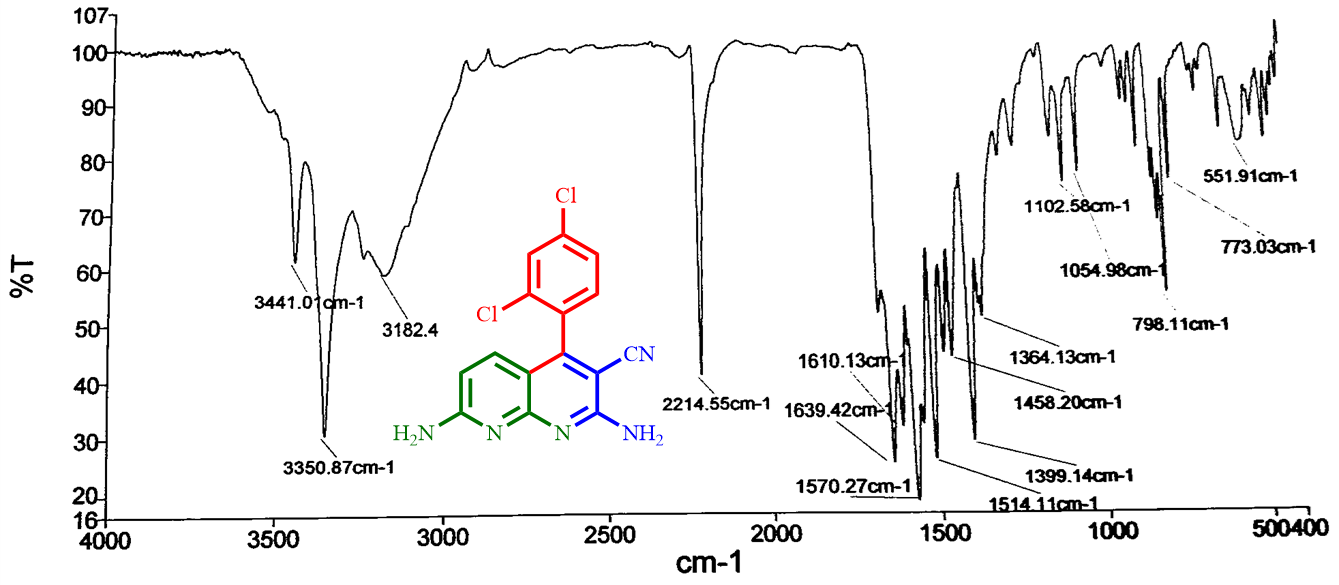


# FT-IR Spectrum of **4j**


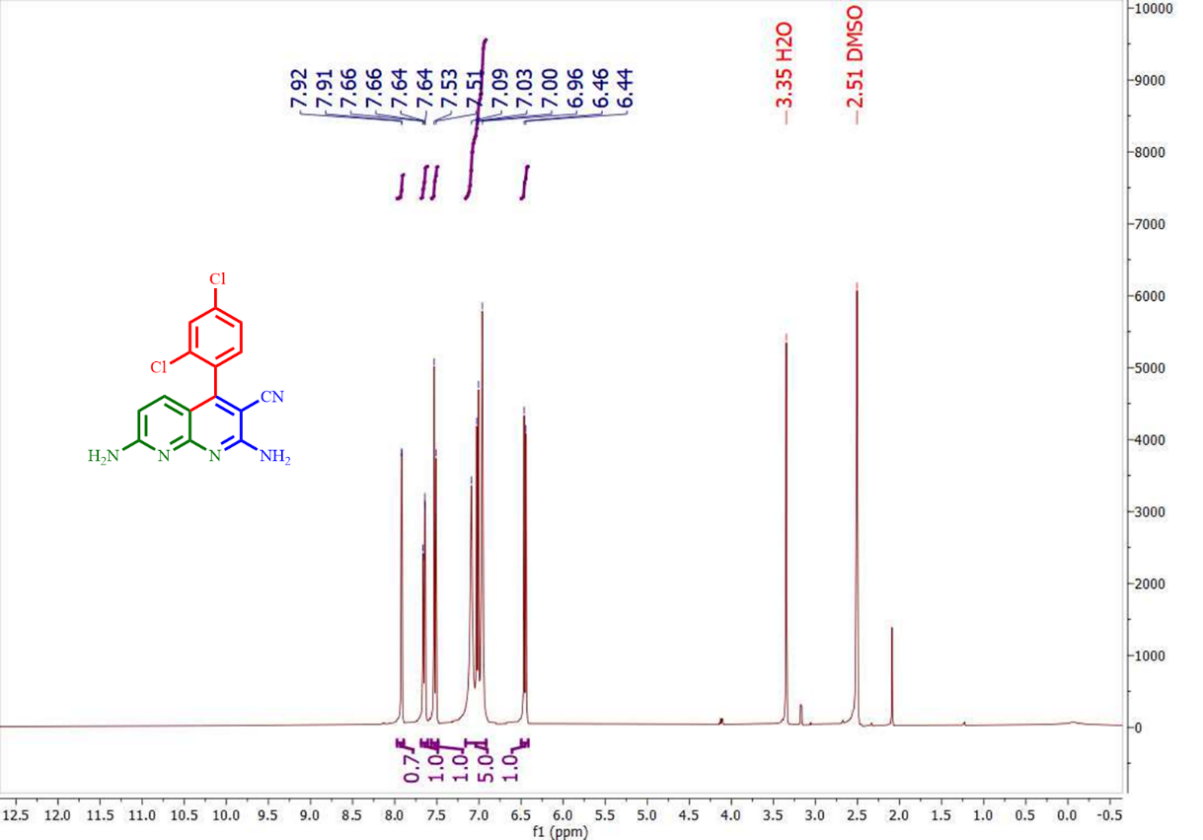


# ^1^H NMR Spectrum of **4j**


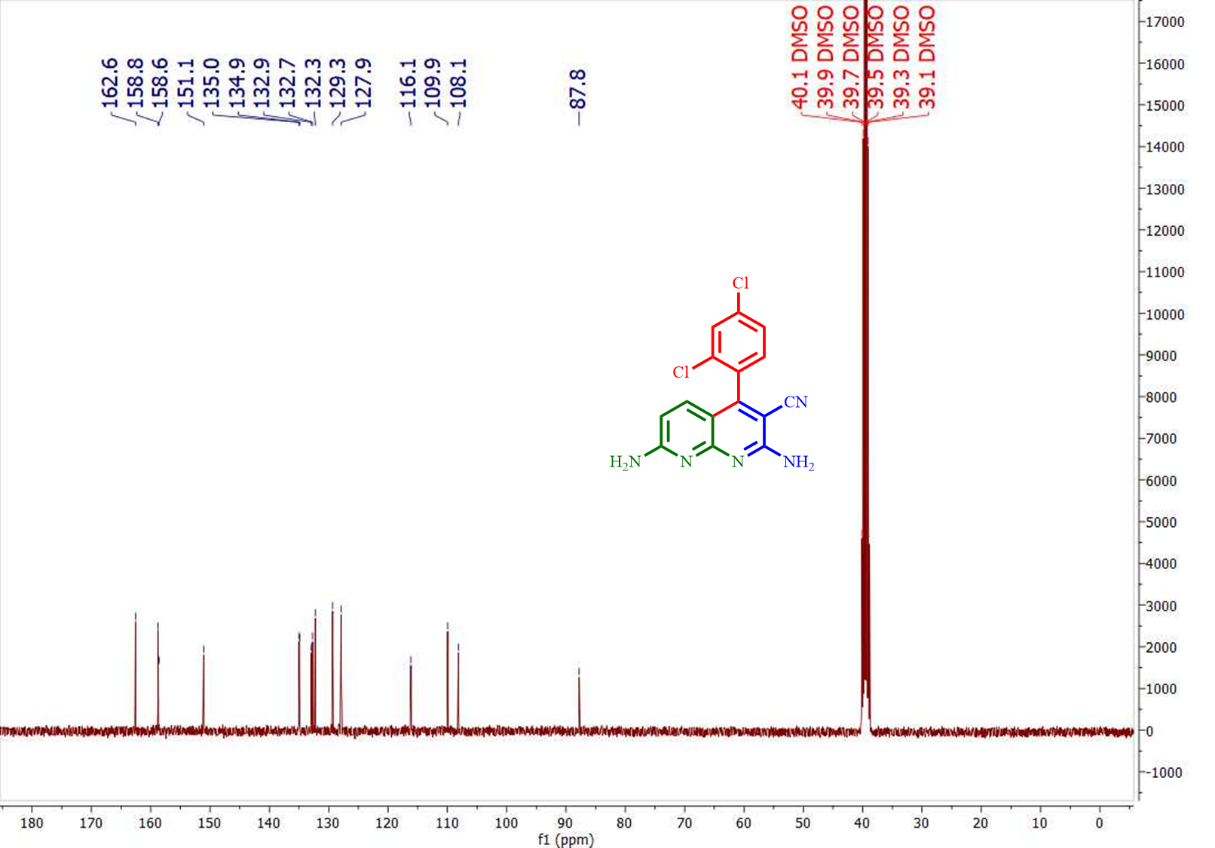


# ^13^C NMR Spectrum of **4j**

**
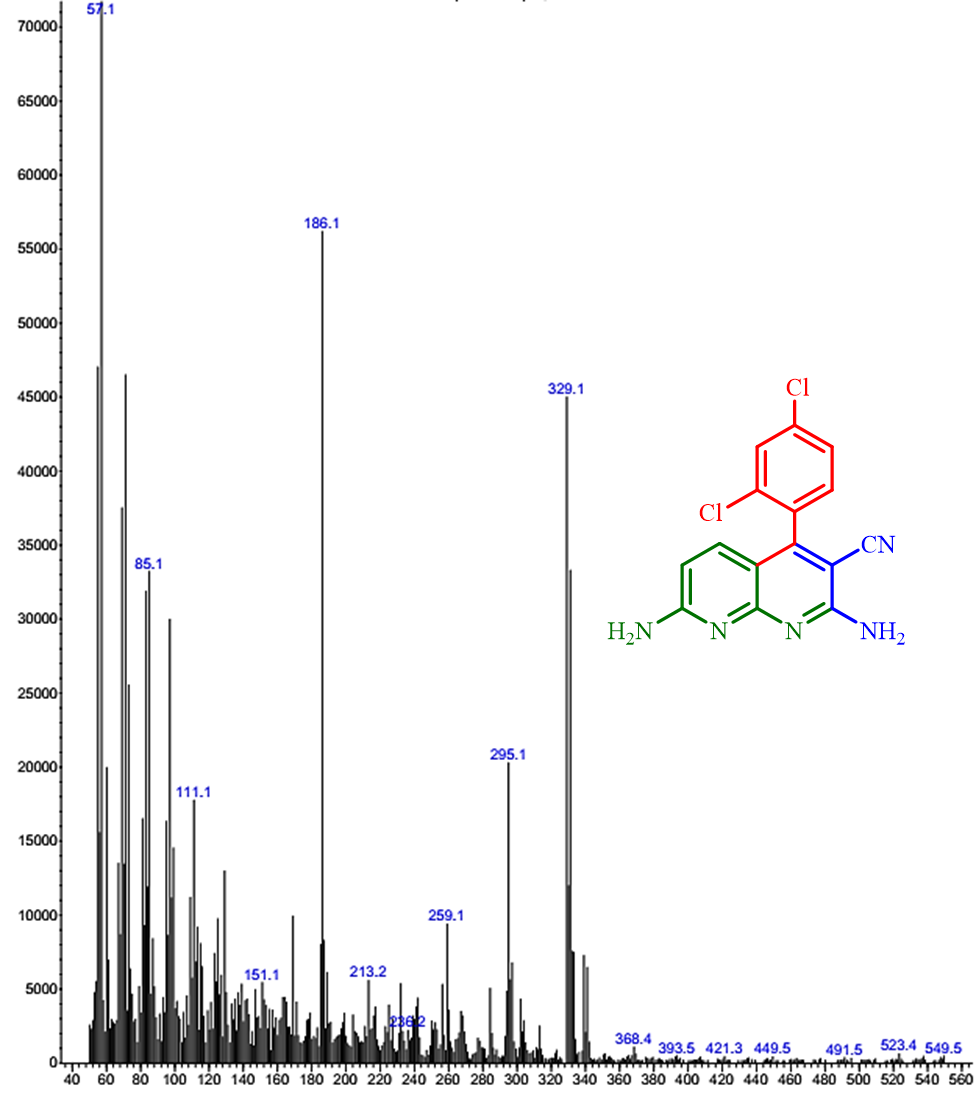
**

# MS Spectrum of **4j**


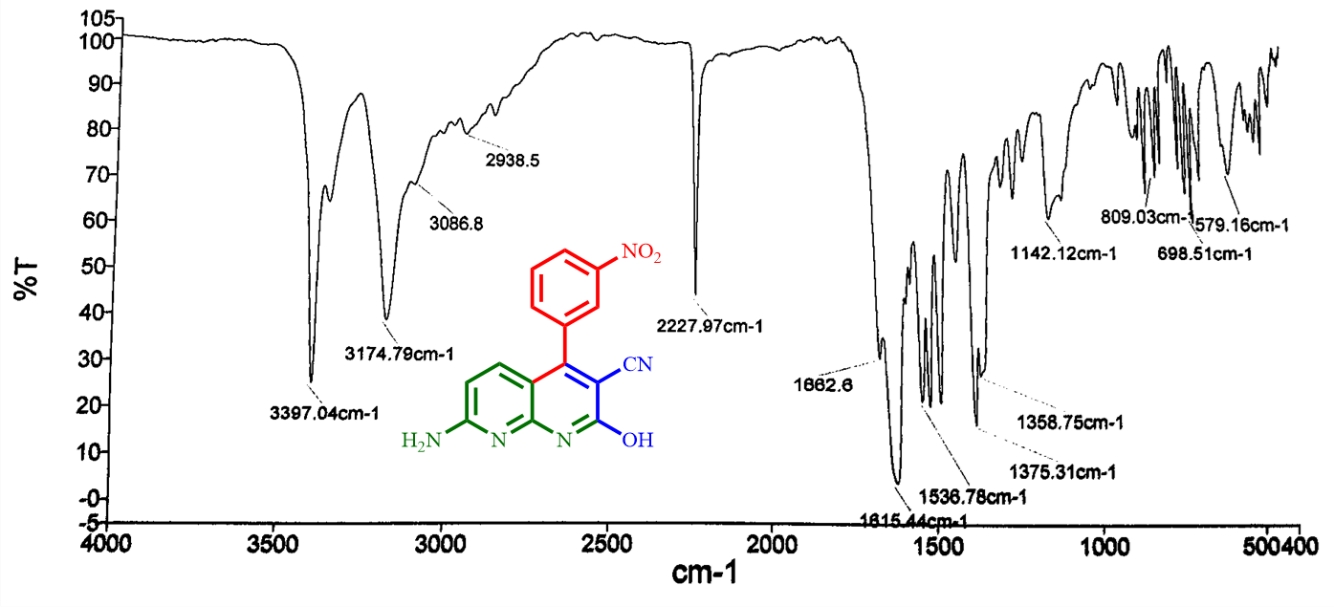


# FT-IR Spectrum of **4k**


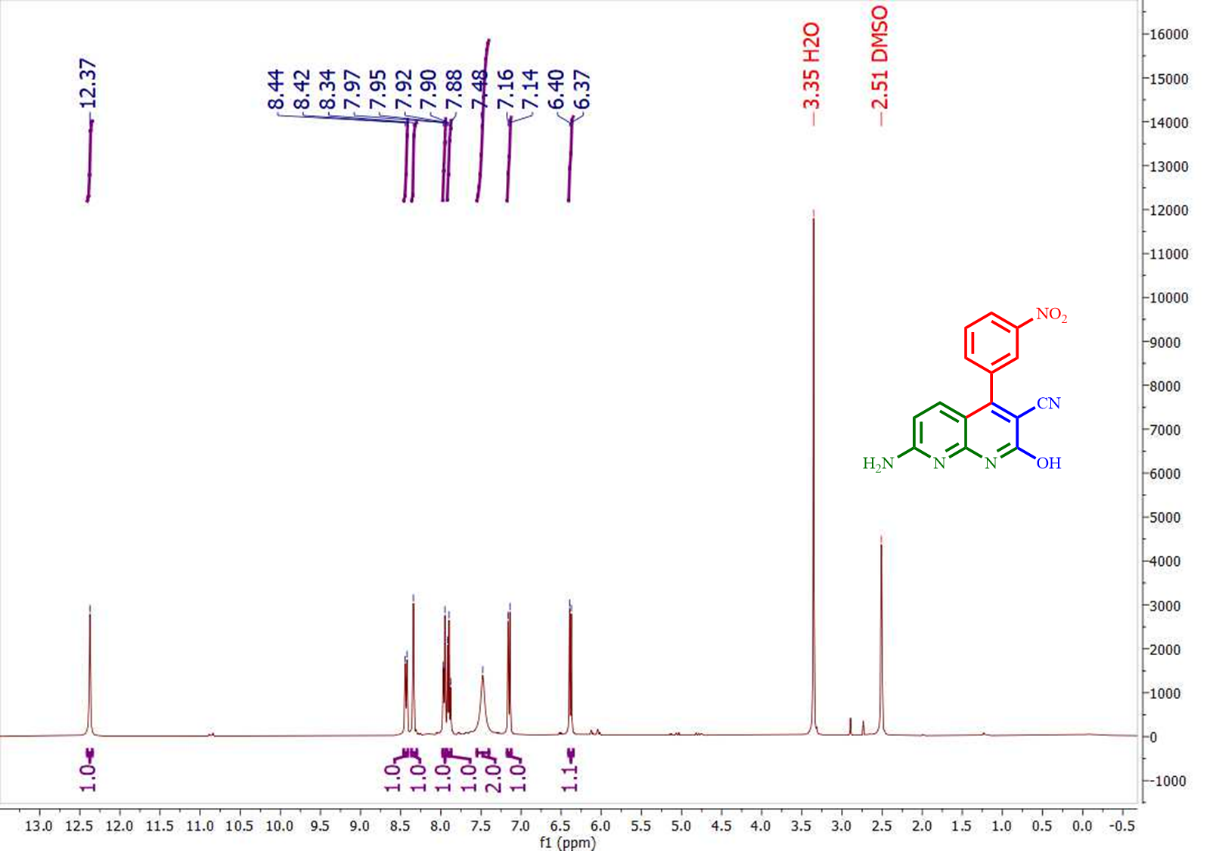


# ^1^H NMR Spectrum of **4k**


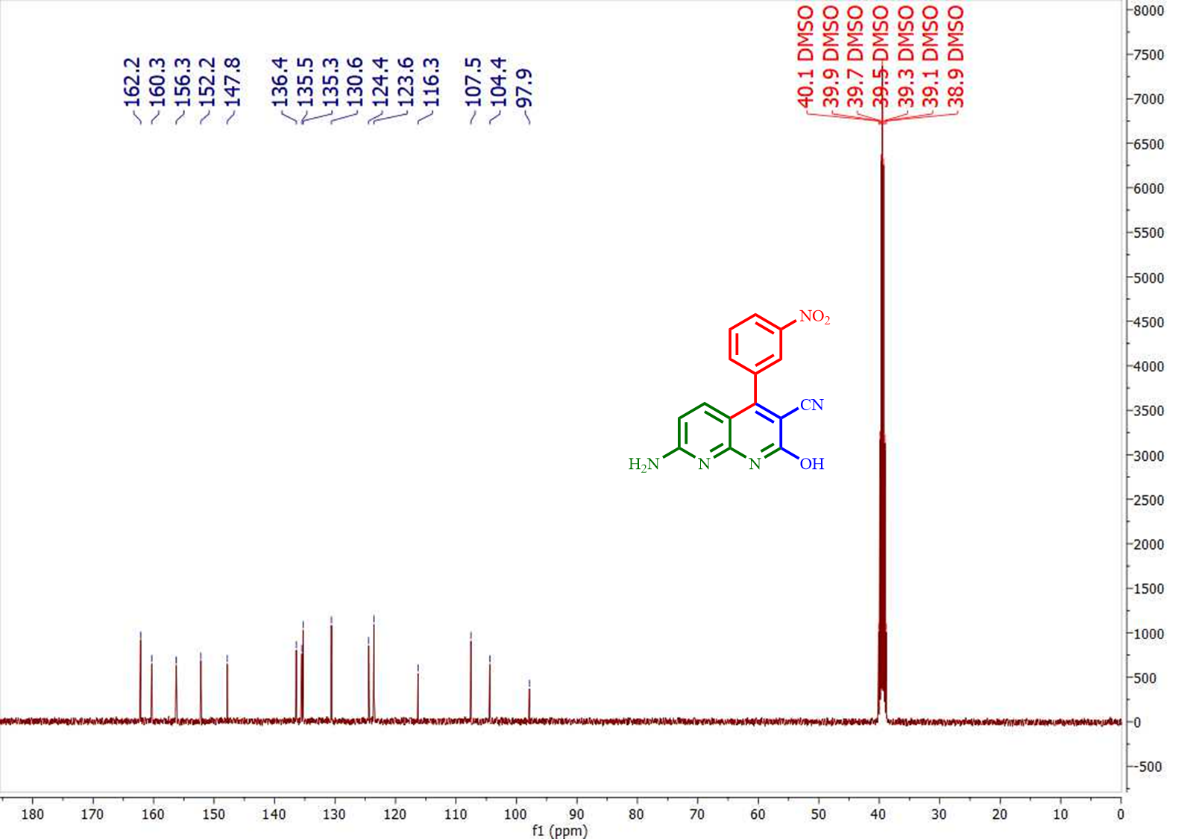


# ^13^C NMR Spectrum of **4k**


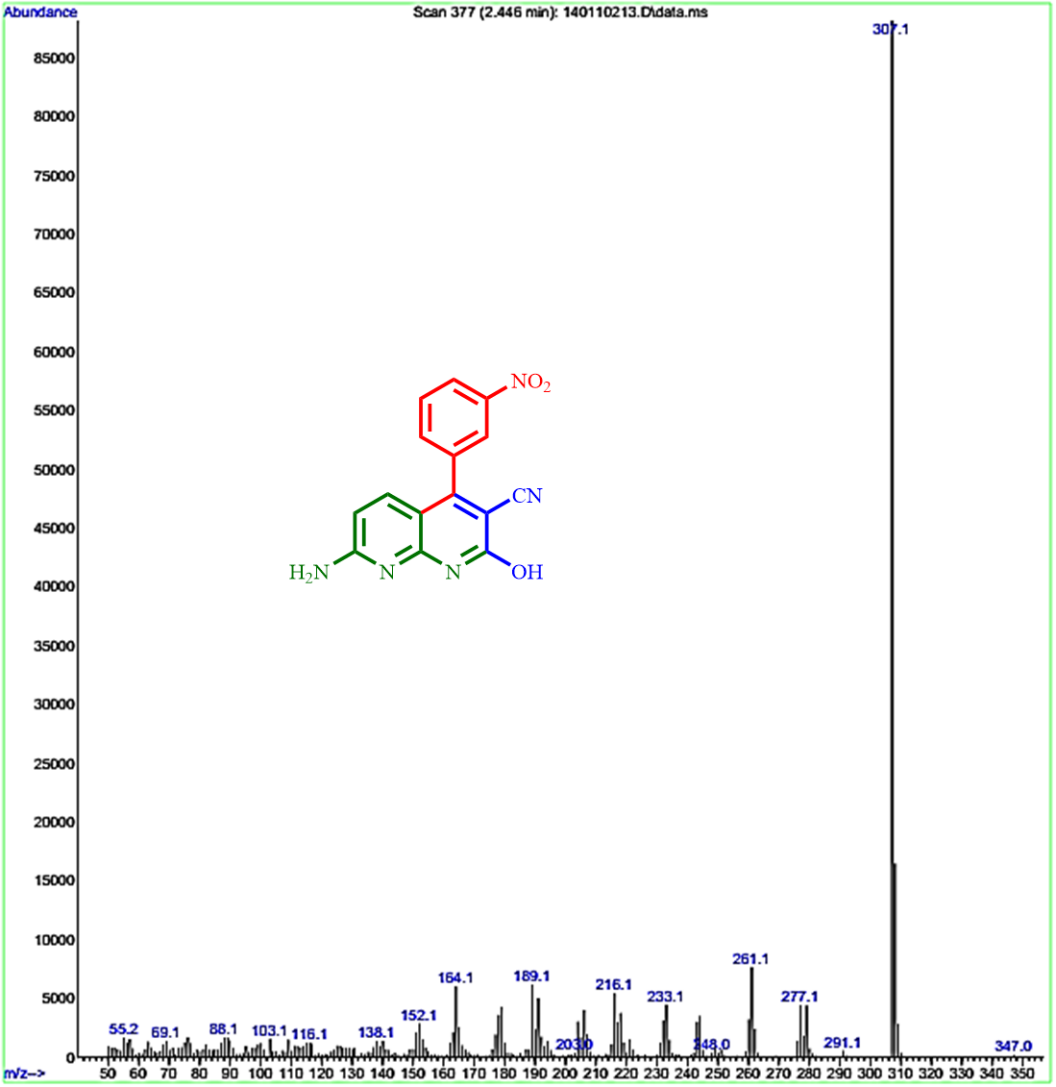


# MS Spectrum of **4k**


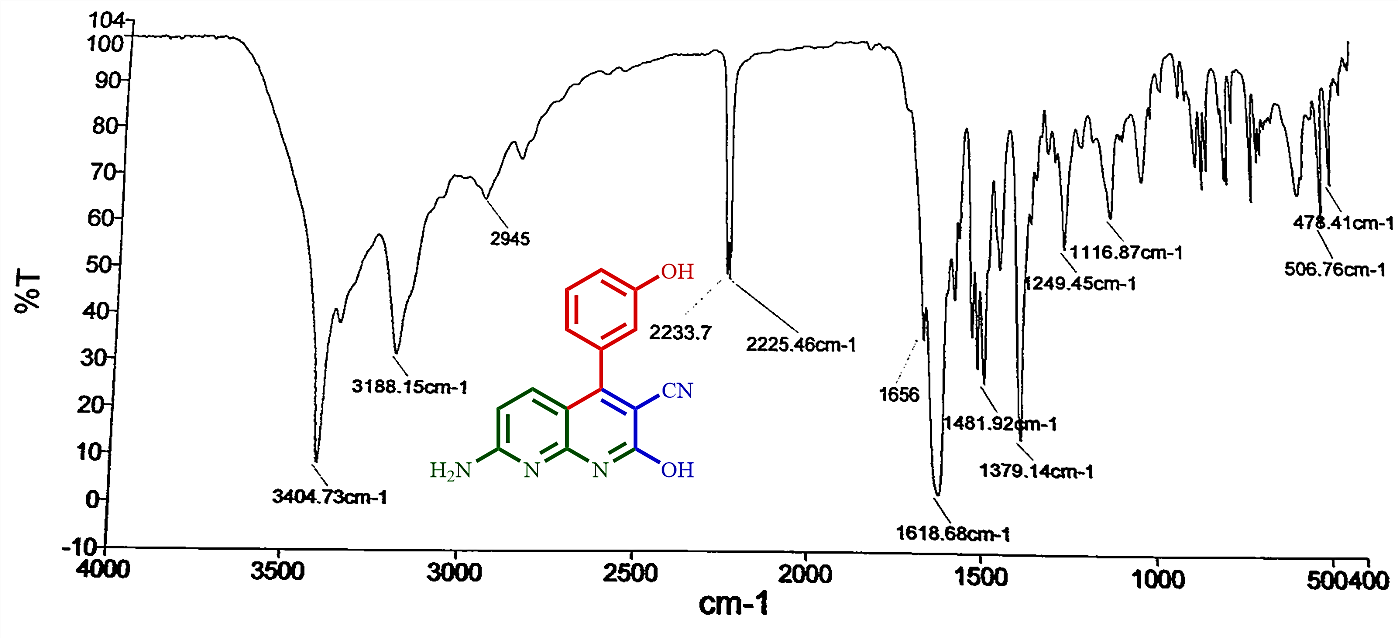


# FT-IR Spectrum of **4l**


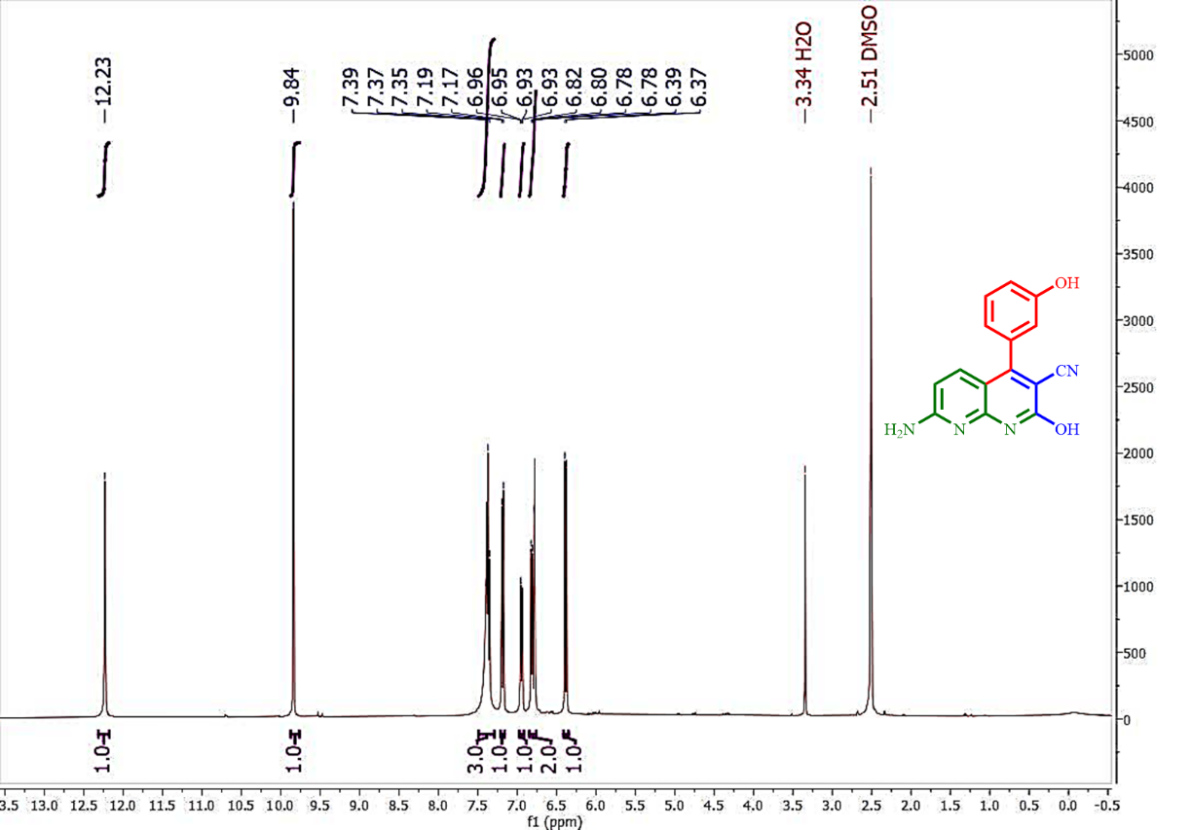


# ^1^H NMR Spectrum of **4l**


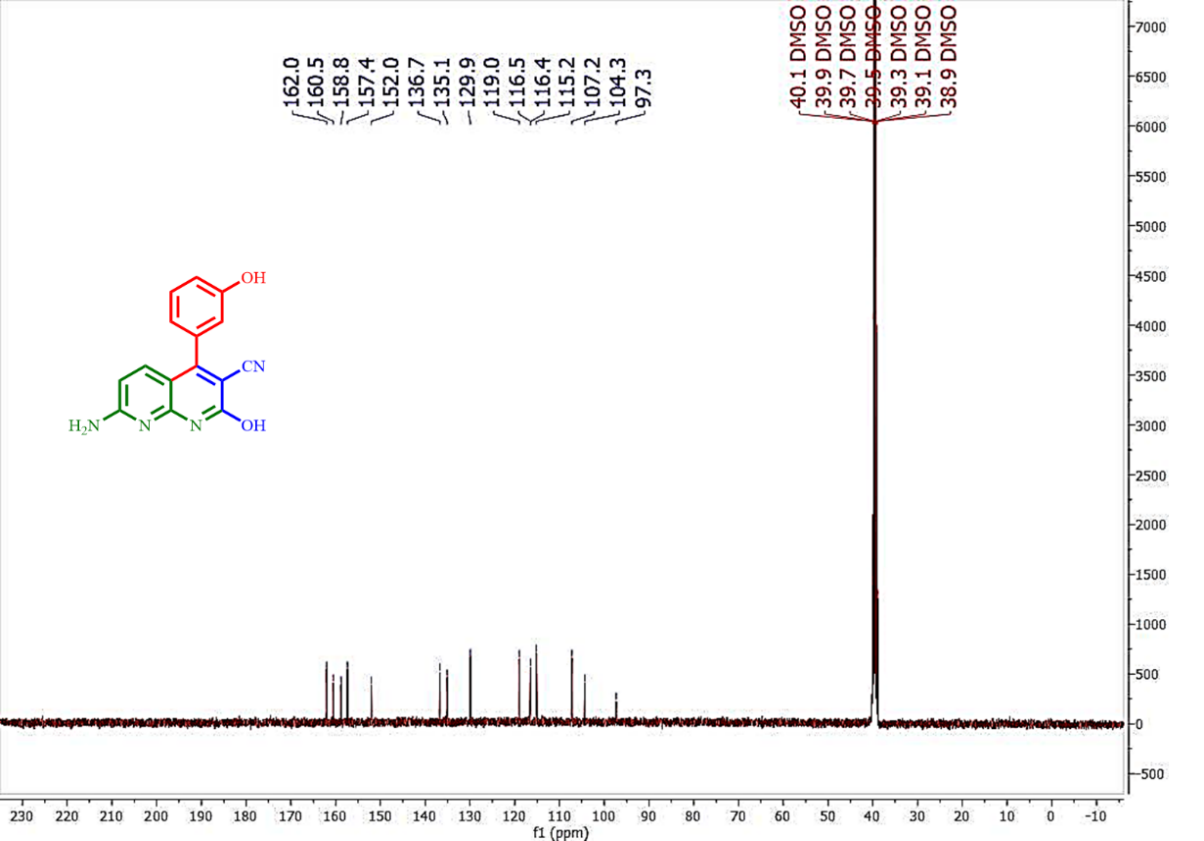


# ^13^C NMR Spectrum of **4l**


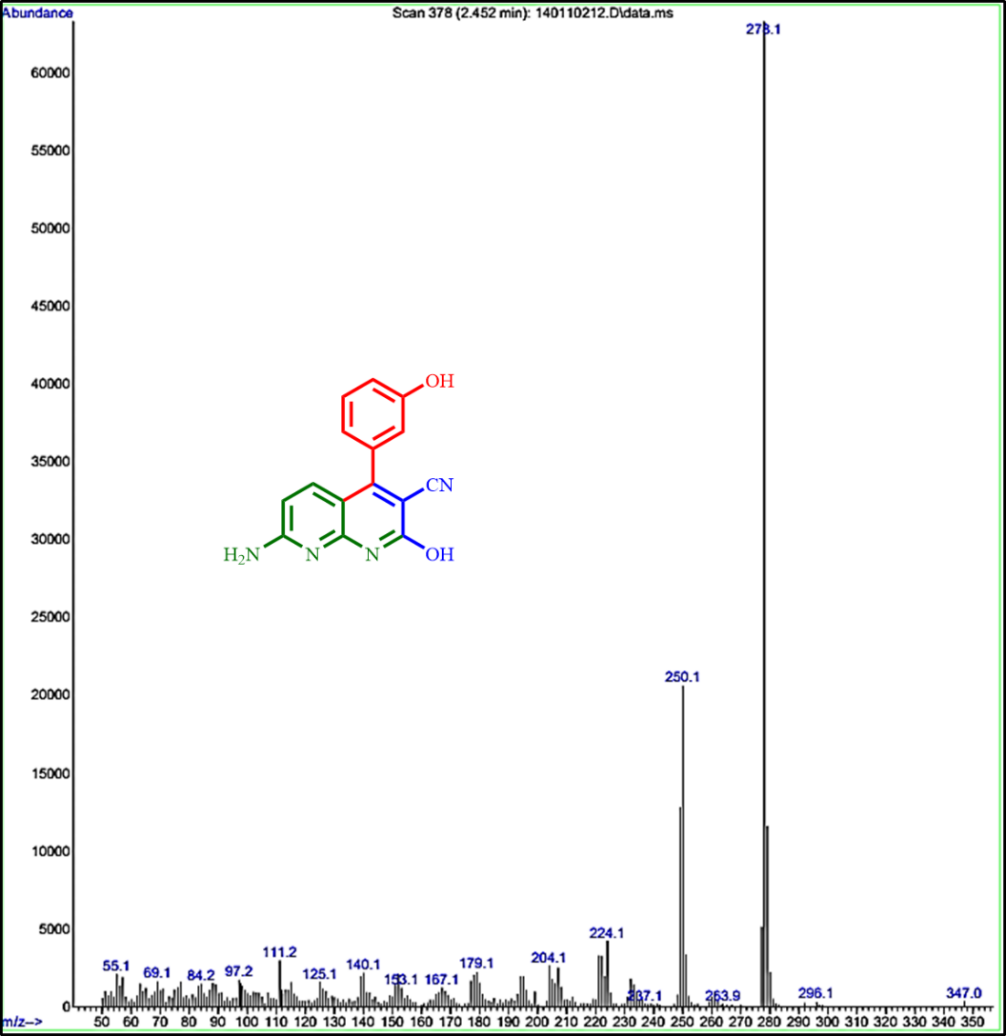


# MS Spectrum of **4l**


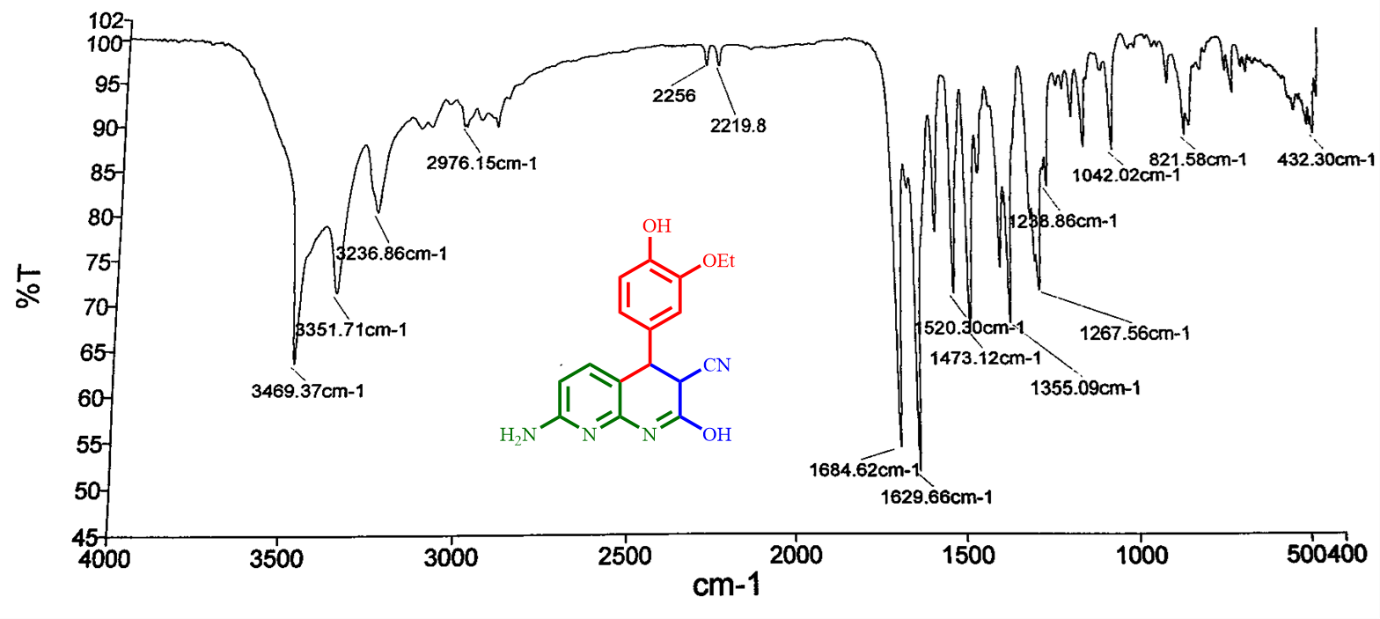


# FT-IR Spectrum of **4m**


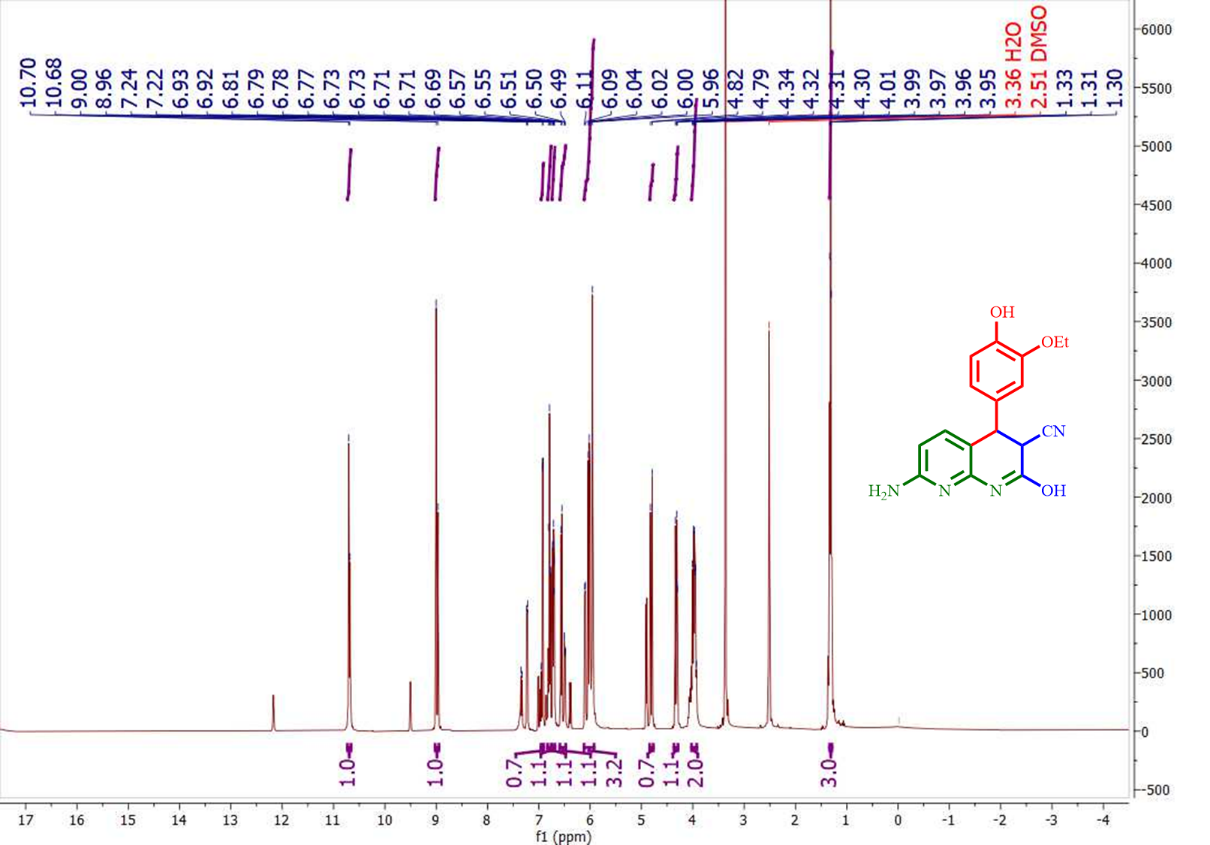


# ^1^H NMR Spectrum of **4m**


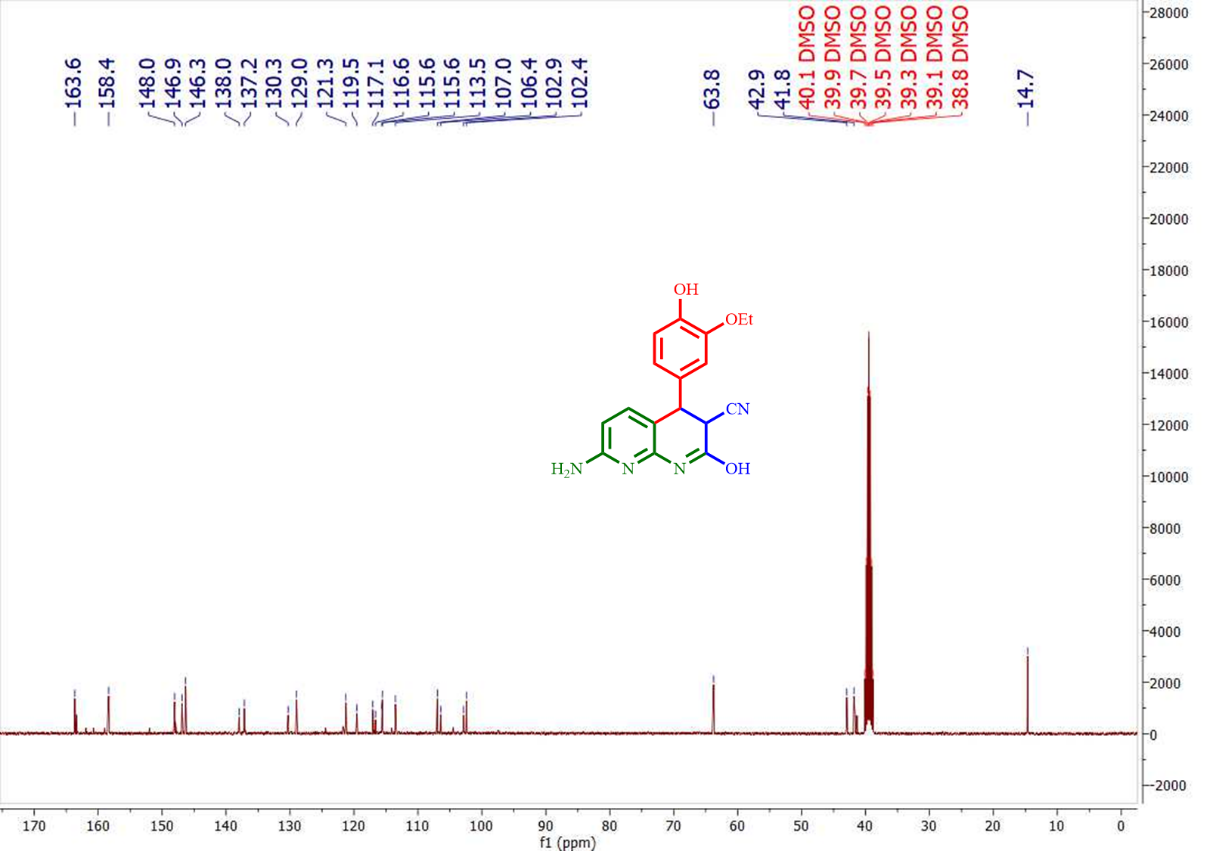


# ^13^C NMR Spectrum of **4m**


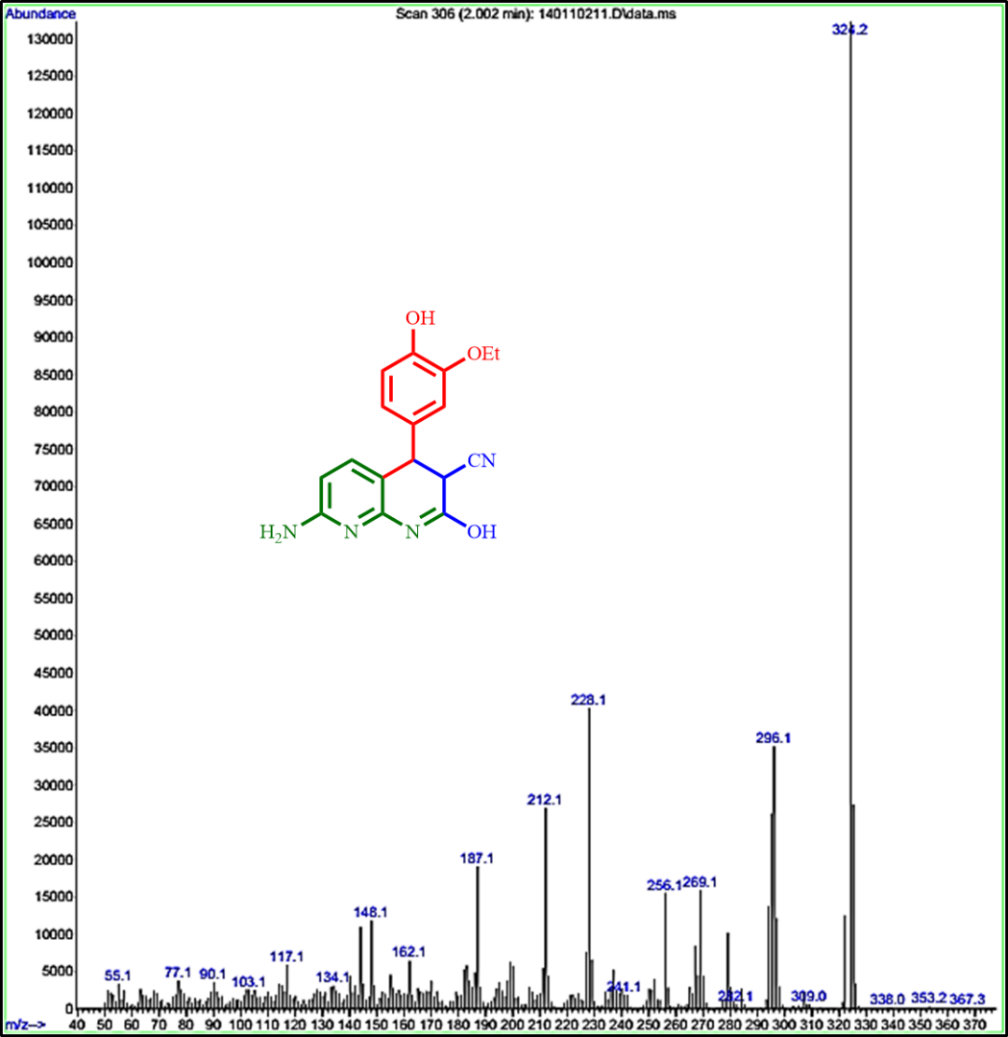


# MS Spectrum of **4m**


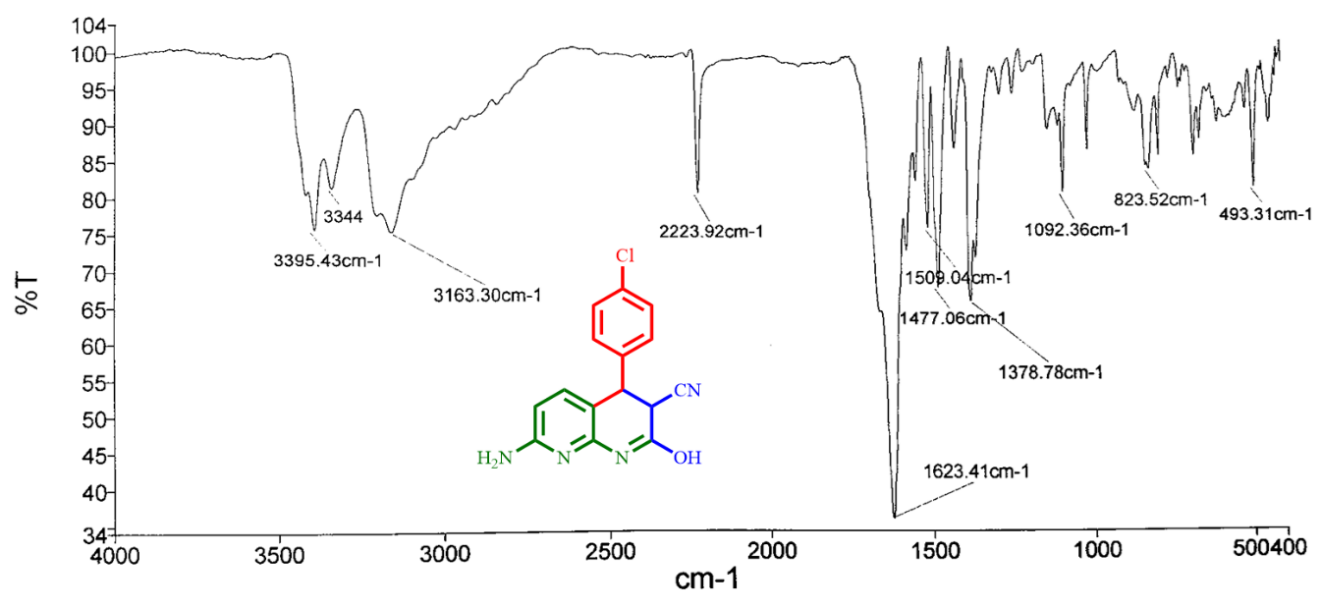


# FT-IR Spectrum of **4n**


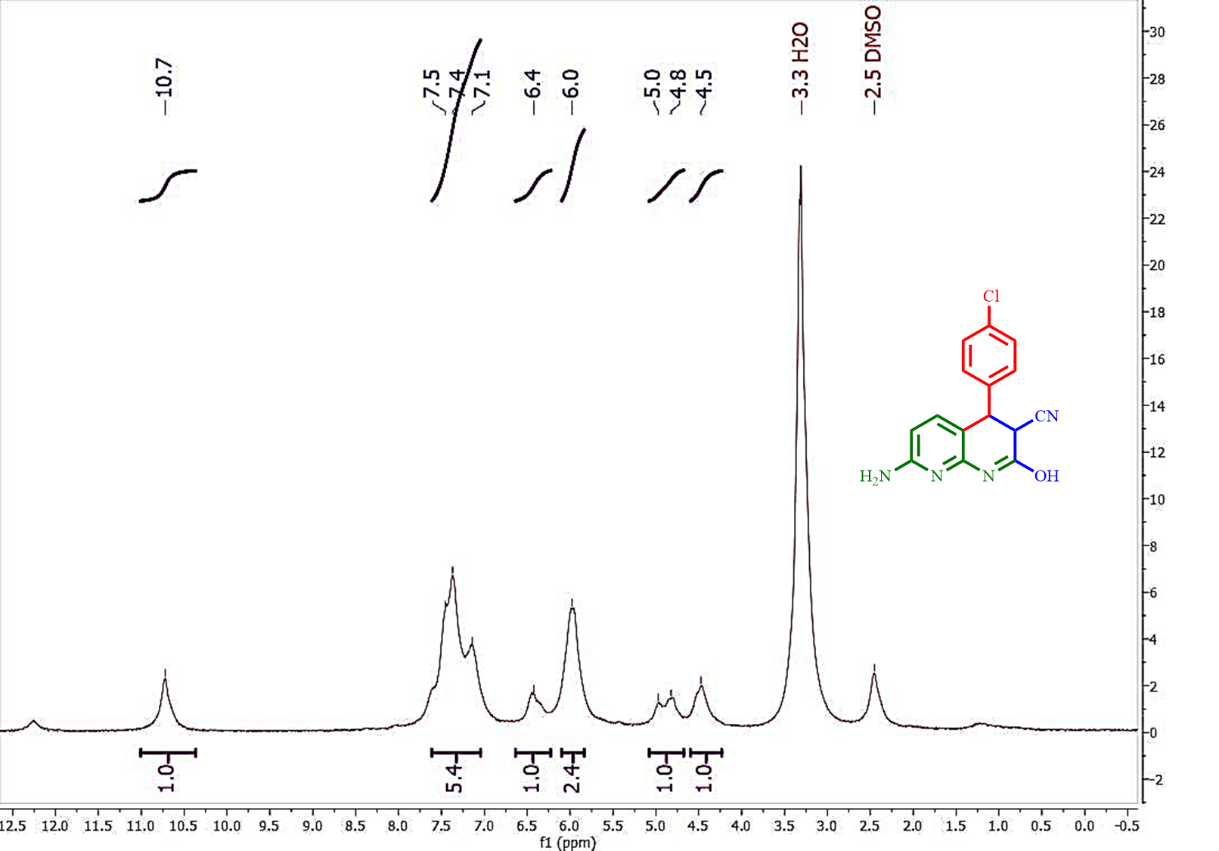


# ^1^H NMR Spectrum of **4n**


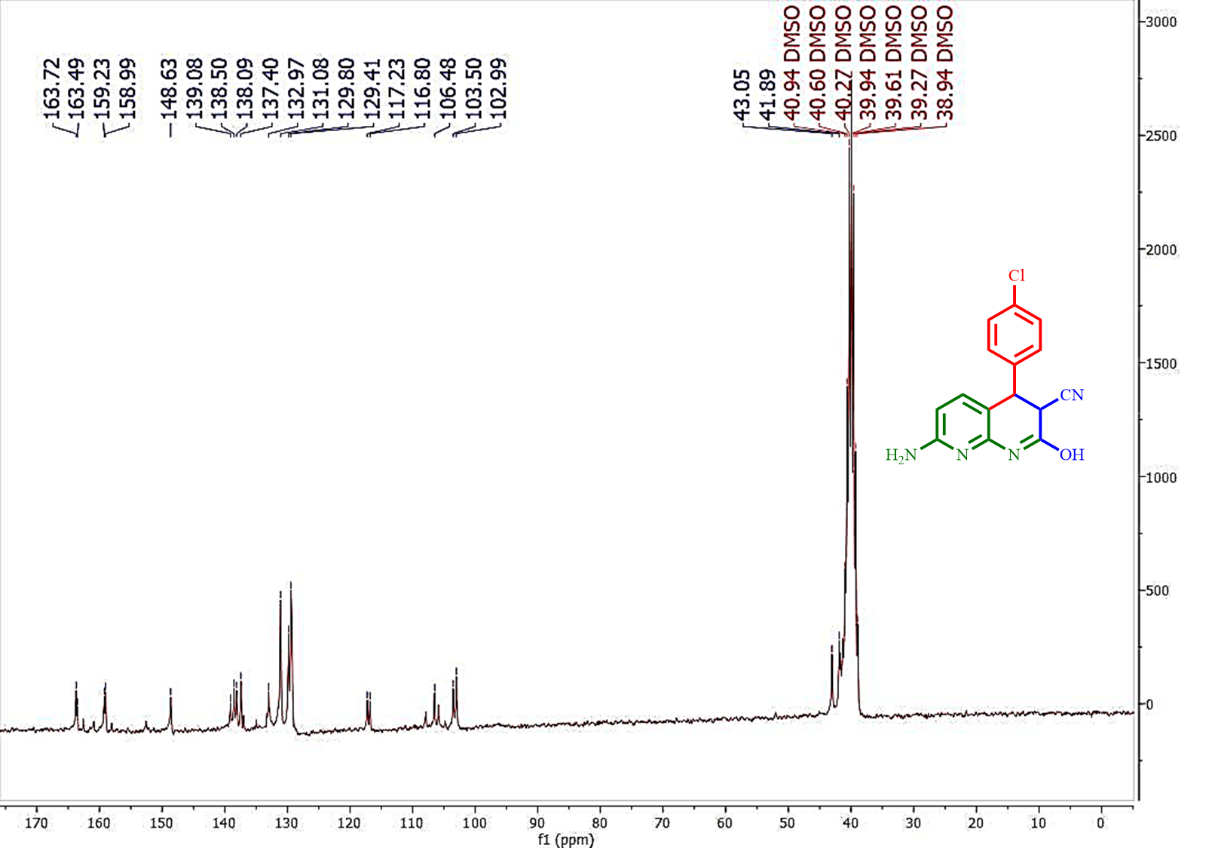


# ^13^C NMR Spectrum of **4n**


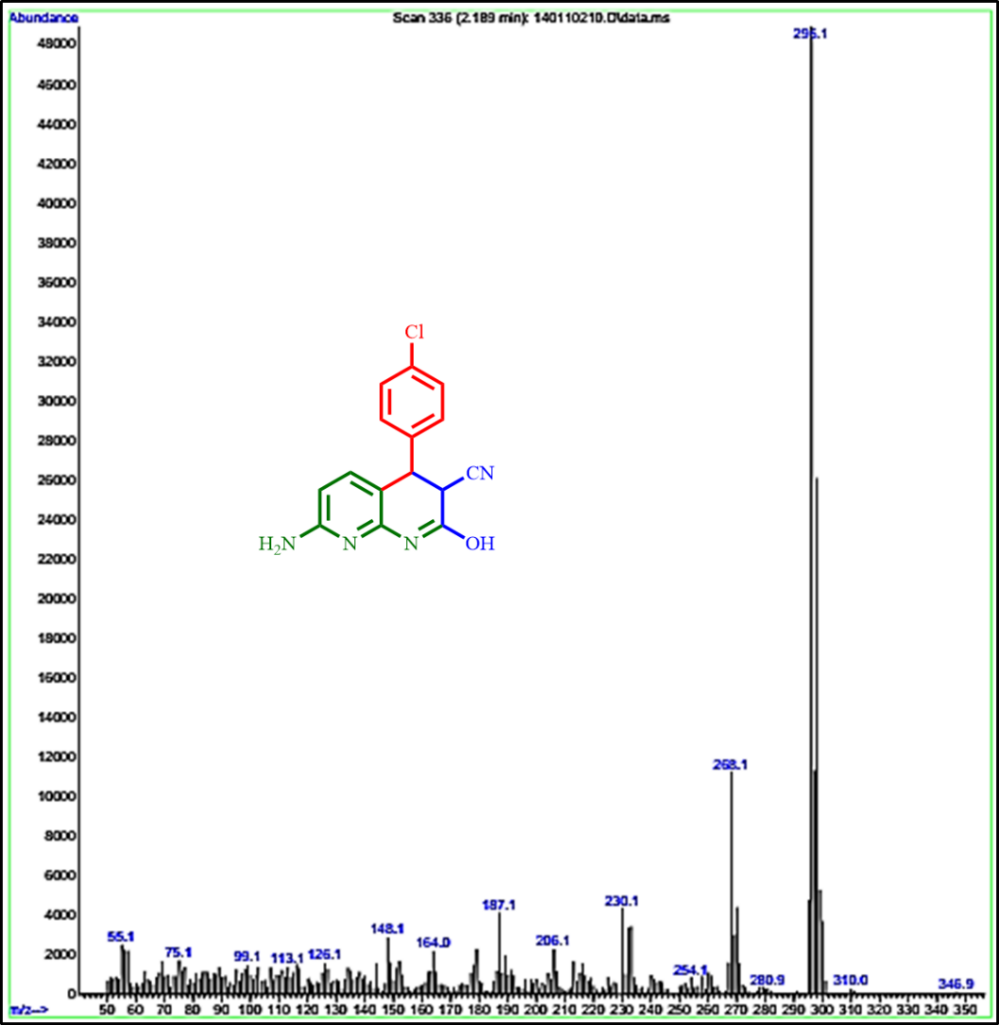


# MS Spectrum of **4n**
